# The Emerging Role of Non-Coding RNAs in Osteogenic Differentiation of Human Bone Marrow Mesenchymal Stem Cells

**DOI:** 10.3389/fcell.2022.903278

**Published:** 2022-05-16

**Authors:** Xiaoying Chen, Wei Xie, Ming Zhang, Yuhan Shi, Shaofen Xu, Haoyu Cheng, Lihong Wu, Janak L. Pathak, Zhichao Zheng

**Affiliations:** ^1^ Affiliated Stomatology Hospital of Guangzhou Medical University, Guangdong Engineering Research Center of Oral Restoration and Reconstruction, Guangzhou Key Laboratory of Basic and Applied Research of Oral Regenerative Medicine, Guangzhou, China; ^2^ Department of Basic Oral Medicine, School and Hospital of Stomatology, Guangzhou Medical University, Guangzhou, China; ^3^ Laboratory for Myology, Department of Human Movement Sciences, Faculty of Behavioural and Movement Sciences, Amsterdam Movement Sciences, Vrije Universiteit Amsterdam, Amsterdam, Netherlands

**Keywords:** BMSCs, ncRNAs, osteogenic differentiation, bone regeneration, bone tissue engineering

## Abstract

Autologous bone marrow-derived mesenchymal stem cells (BMSCs) are more easily available and frequently used for bone regeneration in clinics. Osteogenic differentiation of BMSCs involves complex regulatory networks affecting bone formation phenomena. Non-coding RNAs (ncRNAs) refer to RNAs that do not encode proteins, mainly including microRNAs, long non-coding RNAs, circular RNAs, piwi-interacting RNAs, transfer RNA-derived small RNAs, etc. Recent *in vitro* and *in vivo* studies had revealed the regulatory role of ncRNAs in osteogenic differentiation of BMSCs. NcRNAs had both stimulatory and inhibitory effects on osteogenic differentiation of BMSCs. During the physiological condition, osteo-stimulatory ncRNAs are upregulated and osteo-inhibitory ncRNAs are downregulated. The opposite effects might occur during bone degenerative disease conditions. Intracellular ncRNAs and ncRNAs from neighboring cells delivered via exosomes participate in the regulatory process of osteogenic differentiation of BMSCs. In this review, we summarize the recent advances in the regulatory role of ncRNAs on osteogenic differentiation of BMSCs during physiological and pathological conditions. We also discuss the prospects of the application of modulation of ncRNAs function in BMSCs to promote bone tissue regeneration in clinics.

## 1 Introduction

The bone defect is mainly caused by trauma, severe infection, bone diseases, tumor resection, and various congenital malformations (Gaihre et al., 2017). The number of bone transplantation-related surgery is over two million all over the world (Li et al., 2018). Currently, autologous bone grafts are regarded as the gold standard for bone defect reconstruction (Nicot et al., 2020). The risks of autologous bone grafts such as limited source, infection, pain, loss of sensation, scars, and donor site morbidity limit the clinical applications (Younger and Chapman, 1989; Tessier et al., 2005). Allografts and synthetic bone grafts are used as alternatives to autologous bone grafts (Eppley et al., 2005). However, bone allografts may lead to complications such as fracture, nonunion, and infection (Delloye et al., 2014). While bone substitutes materials such as ceramics have osteoconductivity and weak osteoinductivity. The variable resorption rate and higher brittleness of biomaterial-based bone grafts lead to impaired graft osseointegration (Sohn and Oh, 2019). Stem cell-based approaches for bone tissue engineering have shown promising results in the clinic. The combination of precursor cells, bone grafts, and growth factors have the potential to replace auto-/allo-bone grafts (Steinhardt et al., 2008; El-Rashidy et al., 2017; Zhao et al., 2020). Studies have shown bone marrow-derived mesenchymal stem cells (BMSCs) as a promising source of seed cells for bone tissue engineering applications (Qi et al., 2017; Arthur and Gronthos, 2020; Chen et al., 2021; Jiang et al., 2021). Autologous or human leukocyte antigen matched allogeneic BMSCs are commonly used for bone regeneration in clinics.

**FIGURE 1:**
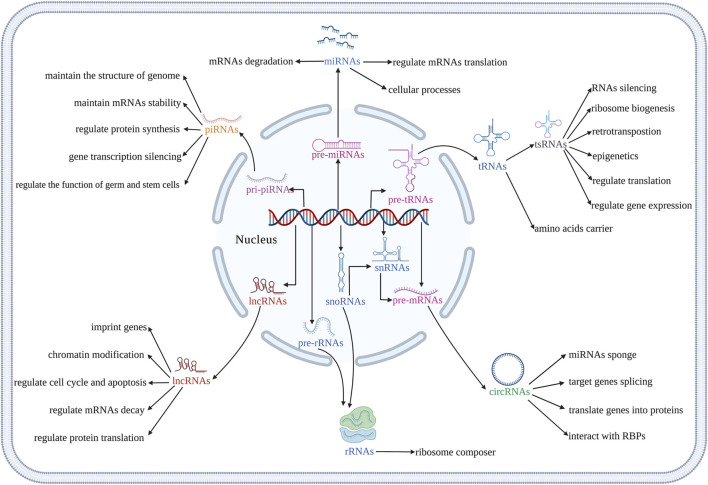
Illustration of biological functions of different kinds of ncRNAs. (Created with BioRender.com).

The osteogenic differentiation of BMSCs is a complex process, which is regulated by multiple signaling pathways. Various non-coding RNAs (ncRNAs) had been reported to regulate the osteogenic differentiation of BMSCs. NcRNAs are transcribed from the genome, do not directly translate into proteins, but participate in the protein translation process of coding mRNAs (Guttman et al., 2013). MicroRNAs (miRNAs), long non-coding RNAs (lncRNAs), circular RNAs (circRNAs), ribosomal RNAs (rRNAs), transfert RNAs (tRNAs), tRNA-derived small RNAs (tsRNAs), small nuclear RNAs (snRNAs), small nucleolar RNAs (snoRNAs) and PIWI-interacting RNAs (piRNAs) are key ncRNAs that regulate basic cellular function such as cell metabolism (Sun et al., 2020a), proliferation (Song et al., 2016), autophagy, apoptosis (Li et al., 2018) as well as various diseases (Peng et al., 2021; Li et al., 2020) (Figure 1). MiRNAs promote mRNAs degradation and regulate mRNAs translation, and participate in various cellular processes (Zealy et al., 2017). LncRNAs have many biological functions, including genes imprinting, chromatin modification, cell cycle, apoptosis, mRNA decay, and protein translation regulation (Zhu et al., 2013). CircRNAs may be by-products of precursor mRNAs. It demonstrated that circRNAs act as a sponge to regulate the function of miRNAs, participate in the splicing of target genes, translate genes into proteins, and interact with RNA-binding proteins (RBPs) (Zang et al., 2020). The report showed that rRNAs are an important part of ribosomes, which are widely involved in cell translation (Sloan et al., 2017). The main function of tRNAs is to carry amino acids and enter ribosomes for protein synthesis with the participation of mRNAs (Liu R. et al., 2021). TsRNAs are produced by tRNAs cleavage, which participate in the processes such as RNAs silencing, ribosome biogenesis, retrotransposition, epigenetics, and regulate translation. tsRNAs also indirectly regulate gene expression by binding RBPs (Chen et al., 2021; Liu et al., 2021a). SnRNAs are the main component of RNA spliceosomes in the post-transcriptional processing of eukaryotes and participate in the processing of precursor RNAs (pre-mRNAs) (Karijolich and Yu, 2010). SnoRNAs modify snRNAs and rRNAs, and participate in the processing of rRNAs during the maturation of ribosomal subunits (Xing and Chen, 2018). PiRNAs maintain the structure of the genome and mRNAs stability, and regulate protein synthesis by binding with members of PIWI protein family (Xu et al., 2020). The piRNA complex formed by the combination of piRNA and PIWI further regulates the function of germ and stem cells by silencing the process of gene transcription (Xu et al., 2020). NcRNAs also mediate osteogenic differentiation of mesenchymal stem cells (MSCs) via regulating various signaling pathways (Yang et al., 2018; Mazziotta et al., 2021). It had been demonstrated that Runt-related transcription factor 2 (RUNX2) is the main regulator responsible for the differentiation of MSCs into preosteoblasts (Bruderer et al., 2014). RUNX2 expression is regulated by several signaling pathways, especially bone morphogenetic protein (BMP) and Wnt (Narayanan et al., 2019). As an important factor in Wnt/β-catenin signaling pathway, β-catenin also regulates genes related to osteogenic differentiation (Zhang and Wang, 2020). Furthermore, Osterix (OSX) is an osteoblast-specific transcription factor, which activates a repertoire of genes during preosteoblasts differentiation into mature osteoblasts (Sinha and Zhou, 2013). Reports from the literature had shown ncRNAs in MSCs as possible targets to induce osteogenic differentiation and bone regeneration (Liu et al., 2018; Peng et al., 20182018; Yang et al., 2019; Hu et al., 2020; Chen et al., 2021). This review focuses on the regulatory role of ncRNAs in the osteogenic differentiation of BMSCs to provide detailed information for the application of ncRNAs in BMSCs-based bone tissue engineering. We also summarize the recent advances, challenges, and prospects of targeting ncRNAs in BMSCs for bone tissue engineering applications in the clinic.

## 2 MiRNAs Involved in the Osteogenic Differentiation of BMSCs

### 2.1 The Biogenesis and Function of miRNAs

MiRNAs are a broad family consisting of single-stranded ncRNAs, ranging in size from 19 to 25 nucleotides (Lu and Rothenberg, 2018). MiRNAs were first discovered in *Caenorhabditis elegans* controlling gene expression in 1993 (Lee et al., 1993). The classical production of miRNAs is a multi-step process that requires the participation of multiple enzymes. The gene encoding miRNA is mainly transcribed by RNA polymerase II in the nucleus to produce a primary miRNA (pri-miRNA). The pri-miRNA is processed into a pre-miRNA by a microprocessor containing the RNase III enzyme Drosha. Exportin-5 is a cytoplasmic transport protein that transports pre-miRNA from the nucleus into the cytoplasm with the assistance of Ran-mediated guanosine triphosphate. Subsequently, the pre-miRNA is further processed by the RNase III enzyme Dicer, and finally, the mature miRNA is released (Lin and Gregory, 2015). Intriguingly, the maturity of some miRNAs bypass one or more steps in the classical pathway. These nonclassical miRNAs are similar to classical miRNAs in structure and function (Divisato et al., 2021). The maturation process of nonclassical miRNAs, derived from introns, snoRNAs, endogenous short hairpin RNAs, and tRNAs, does not depend on the processing of *Drosha*/*Dgcr8*, but only *Dicer* (Abdelfattah et al., 2014). *Dicer* is almost indispensable in the production of both standard and non-standard miRNA. But surprisingly, several miRNAs can also be produced in the absence of *Dicer*, such as miR-451 (Abdelfattah et al., 2014).

Usually, the gene silencing mechanism is determined by the degree and nature of complementarity between the miRNA binding sites and the 3′ untranslated region (3′UTR) of its target genes. The target gene undergoes degradation when the miRNAs and target genes are fully complementary (Huntzinger and Izaurralde, 2011). However, miRNAs inhibit the translation of the target genes while the binding is not complementary (Huntzinger and Izaurralde, 2011). MiRNAs are involved in various cellular processes, such as proliferation, differentiation, apoptosis, etc., (Morgado et al., 2016; Wang et al., 2019; Ding et al., 2019). *Drosha* and *Dicer* are endonucleases involved in miRNA synthesis, which are closely related to the osteogenic differentiation of BMSCs (Macfarlane and Murphy, 2010; Feng et al., 2020). Knockout of *Dicer* or *Drosha* inhibits the osteogenic differentiation of BMSCs (Oskowitz et al., 2008). Furthermore, miRNAs could directly regulate the osteogenic differentiation of BMSCs through complex mechanisms (Mazziotta et al., 2021).

### 2.2 Mechanisms Involved in miRNAs-Induced Osteogenic Differentiation of BMSCs

#### 2.2.1 The Regulation in Physiological Conditions

**FIGURE 2:**
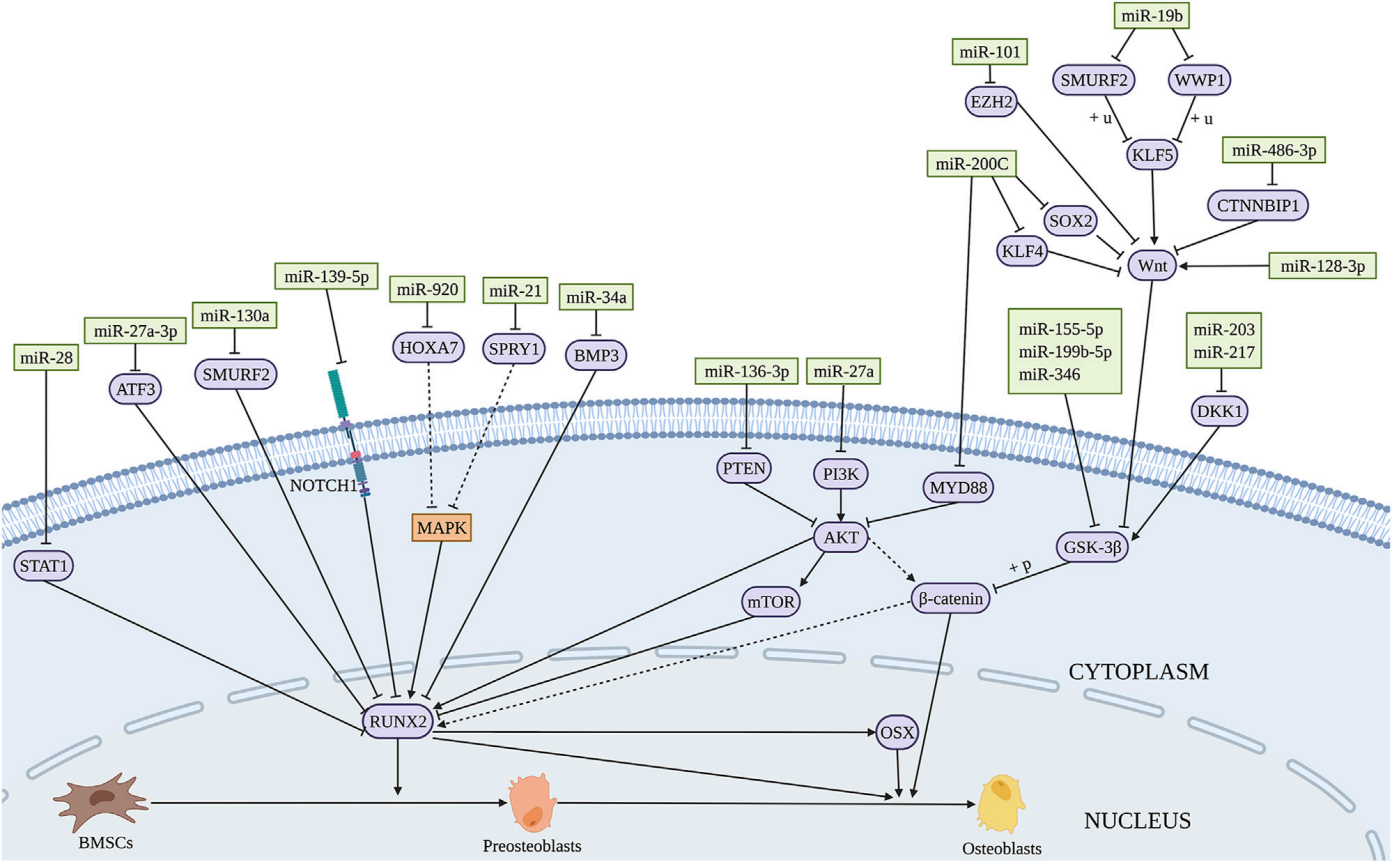
Illustration of the role and regulatory mechanism of miRNAs-induced osteogenic differentiation of BMSCs. Several miRNAs promote osteogenic differentiation of BMSCs by regulating the expression of target genes and related signaling pathways. (Created with BioRender.com).

A range of miRNAs has the potential to promote osteogenic differentiation of BMSCs. BMP3 is the most abundant member of BMP family, accounting for about 65% of the total content (Bahamonde and Lyons, 2001). MiR-34a promotes the osteogenic differentiation of BMSCs by directly targeting BMP3 (Zeng et al., 2021). MiR-19b significantly promotes the osteogenic differentiation of BMSCs by targeting WW domain-containing E3 ubiquitin protein ligase 1 (WWP1) and Samd ubiquitin regulatory factor 2 (SMURF2) through the kruppel like factor (KLF) 5/β-catenin signaling pathway (Huang et al., 2021) (Figure 2 and Table 1). Short-term or intermittent hypoxia is an inducer of osteogenic differentiation of BMSCs (Ciapetti et al., 2016; Sha et al., 2017). Epigallocatechin gallate promotes osteogenic differentiation of BMSCs under hypoxia, in which miR-210 is upregulated and targets to inhibit ephrin-A3 (EFNA3) (Qiu et al., 2016). Overexpression of miR-27b and miR-130a promotes the osteogenic differentiation of BMSCs by directly targeting peroxisome proliferator-activated receptor γ (PPARγ) to increase RUNX2 expression (Seenprachawong et al., 2018). Wang et al. found that miR-28 upregulation inhibits signal transducer and activator of transcription 1 (STAT1) expression, thus promoting the osteogenic differentiation of BMSCs (Wang et al., 2022). The expression level of miR-34c-5p is increased during the osteogenic differentiation of BMSCs (Liu et al., 2021b). B-cell lymphoma 2 (BCL2) is an anti-apoptotic protein (Ebrahim et al., 2016), and miR-34c-5p promotes the osteogenic differentiation of BMSCs via inhibition of BCL2 expression and upregulation of RUNX2 and osteocalcin (OCN) (Liu et al., 2021b). MiR-99a-5p boosts osteogenic differentiation of BMSCs, while downregulation of miR-99a-5p expression inhibits the differentiation, but the regulatory mechanism is unclear (Xu et al., 2018).

**TABLE 1:** MiRNAs that promote osteogenic differentiation of BMSCs and underlying mechanisms.

miRNA	Study model	Signaling pathway	Effect	References
miR-19b	BMSCs from healthy donors	Targets WWP1/SMURF2 to promote KLF5 expression via the Wnt/β-catenin signaling	Promotes the expression of ALP, RUNX2, and COL1	Huang et al. (2021)
miR-21	BMSCs *in vitro*	——	Promotes the expression of COL1, RUNX2, OPN, and OCN, as well as osteogenic differentiation	Zhongshan Whao et al. (2016)
	BMSCs from healthy donors and OP patients and ectopic bone formation in nude mice	Targets SPRY1 to indirectly activate FGF and ERK-MAPK signaling pathways	Promotes the expression of ALP, RUNX2, and OSX, as well as bone formation	Yang et al. (2013)
miR-27a	BMSCs from healthy donors and OP patients	Targets MEF2C	Promotes the expression of ALP, RUNX2, and OCN, as well as bone formation	You et al. (2016)
miR-27a	BMSCs from femoral neck fracture and ONFH patients	Targets PI3K to regualte PI3K/Akt/mTOR signaling pathway	Promotes the expression of ALP, BMP2, COL1A1, OSX, and RUNX2	Tang et al. (2021)
miR-27a-3p	BMSCs from healthy donors and OP patients	Targets ATF3	Promotes the expression of ALP, RUNX2, and OCN	Fu et al. (2019)
miR-27b, miR-130a	BMSCs *in vitro*	Targets PPARγ	Promote the expression of RUNX2, OSX, and COL1A1	Seenprachawong et al. (2018)
miR-28	BMSCs *in vitro*	Targets STAT1	Promotes the expression of ALP and RUNX2	Wang et al. (2022)
miR-34a	BMSCs *in vitro*	Targets BMP3	Promotes the expression of RUNX2, ALP, OSX, COL1, and OCN, as well as alleviates OP progression	Zeng et al. (2021)
miR-34c-5p	BMSCs *in vitro*	Targets BCL2	Promotes the expression of RUNX2 and OCN	Bin Liu et al. (2021)
miR- 99a-5p	BMSCs *in vitro*	——	Increases calcium salt deposition	Xu et al. (2018)
miR-101	BMSCs from *in vitro* and skull defects model of nude mice	Targets EZH2 to activate Wnt/β-catenin signaling pathway	Promotes the expression of RUNX2, ALP, OPN, and OCN, as well as bone repair	Hongrui Wang et al. (2016)
miR-128–3p	BMSCs from patients with open fractures and iliac bone grafts	Targets WNT3A to activate Wnt signaling	Promotes the expression of OCN, RUNX2, and BMP2	Lin et al. (2021)
miR-130a	BMSCs *in vitro*	Targets SMURF2	Promotes the expression of ALP, OCN, RUNX2, and OSX	Lin et al. (2019)
miR-136–3p	BMSCs *in vitro*	Targets PTEN	Promotes the expression of OCN, as well as rescues ethanol-mitigated bone formation ability	Yixuan Chen et al. (2020)
miR-146a	BMSCs *in vitro*	——	The inhibition of miR-146a decreases the expression of RUNX2, COL1, ALP and OCN	Xianfeng Zhou et al. (2016)
miR-148b-3p	BMSCs *in vitro*	——	Promotes the expression of ALP and COL1	Mollazadeh et al. (2019)
miR-155–5p	BMSCs from femoral neck fracture and ONFH patients	Targets GSK-3β to activate β-catenin signaling	Promotes the expression of RUNX2, COL1A1, ALP, OCN, and OSX	Fei Wu et al. (2021)
miR-199b-5p	BMSCs *in vitro*	Targets GSK-3β to activate GSK-3β/β-catenin signaling pathway	Promotes the expression of ALP and RUNX2	Ruibo Zhao et al. (2016)
miR-200c	BMSCs *in vitro*	Targets MYD88 to activate AKT/β-catenin signaling pathway	Promotes the expression of BMP2, RUNX2, RANKL, OSX, OCN, OPN, and COL1	Xia et al. (2019)
miR-200c	BMSCs *in vitro*	Targets SOX2 and KLF4 to activate Wnt/β-catenin signaling	Promotes the expression of RUNX2 and OCN, as well as bone formation and bone regeneration	Akkouch et al. (2019)
miR-200c	BMSCs *in vitro*	——	Promotes the expression of ALP and RUNX2, as well as calcium content	Hong et al. (2016)
miR-203	BMSCs from healthy donors and OP patients	Targets DKK1	Promotes the expression of ALP, OCN and RUNX2	Qiao et al. (2018)
miR-210	BMSCs *in vitro*	Targets EFNA3	Promotes the expression of ALP, BMP2, and RUNX2	Qiu et al. (2016)
miR-217	BMSCs from femoral neck fracture and ONFH patients	Targets DKK1	Promotes the expression of RUNX2 and COL1A1	Dai et al. (2019)
miR-335–5p	BMSCs *in vitro*	——	Promotes the expression of BMP2, OCN, OPN, and RUNX2	Zhenming Huang et al. (2020)
miR-346	BMSCs *in vitro*	Targets GSK-3β to activate Wnt/β-catenin pathway	Promotes the expression of RUNX2, ALP, and OPN	Wang et al. (2013)
miR-486–3p	BMSCs from healthy donors and OP patients	Targets CTNNBIP1 to activate Wnt/β-catenin signaling	Promotes the expression of RUNX2, ALP, COL1A1, and OCN	Zheng Zhang et al. (2021)
miR-548d-5p	BMSCs *in vitro*	Targets PPARγ	Promotes the expression of RUNX2 and OCN	Sun et al. (2014)
miR-664a-5p	BMSCs *in vitro*	Targets HMGA2	Promotes the expression of RUNX2, ALP, and OCN	Yan Zhang et al. (2020)
miR-920	BMSCs from healthy donors and OP patients	Targets HOXA7 through MAPK signaling pathway	Promotes the expression of ALP and OSX	Zha et al. (2020)

#### 2.2.2 The Regulation of miRNAs in Pathological Conditions

MiRNAs participate in the BMSCs differentiation in several diseases such as osteoporosis (OP), osteonecrosis, etc. OP is one common disease in the elderly and menopausal women (Tella and Gallagher, 2014). Postmenopausal osteoporosis (PMOP) is a common type of OP caused by estrogen deficiency. The osteogenic differentiation potential of BMSCs is compromised in OP patients (Zeng et al., 2021). The expression of miR-486-3p is significantly downregulated in the bone marrow of OP patients. Catenin beta interacting protein 1 (CTNNBIP1) is an inhibitor of Wnt/β-catenin signaling and mechanistically, miR-486-3p promotes the osteogenic differentiation of BMSCs by targeting CTNNBIP1 to active the Wnt/β-catenin pathway (Zhang et al., 2021). MiR-27a-3p shows lower serum level in OP patients compared with the control group. Overexpression of miR-27a-3p promotes the osteogenic differentiation of BMSCs by directly targeting activating transcription factor (ATF) 3 (Fu et al., 2019). MiR-27a is significantly decreased in the serum of PMOP patients. And miR-27a promotes the expression of osteogenesis-related markers such as alkaline phosphatase (ALP), RUNX2, and OCN by targeting myocyte enhancer factor 2C (MEF2C) (You et al., 2016). Similarly, miR-203 which is downregulated in the serum of OP patients increases the levels of osteogenic genes by targeting dickkopf 1 (DKK1) (Qiao et al., 2018). DKK1 is an important molecule in the development of embryo and adult bone, and is involved in the occurrence of OP (Glinka et al., 1998). Sprouty 1 (SPRY1) is a negative regulator of fibroblast growth factor (FGF) and extracellular signal-regulated kinase-mitogen-activated protein kinase (ERK-MAPK) signaling pathways, which is considered to be related to promoting MSCs osteogenesis (Ge et al., 2007; Ng et al., 2008). Yang et al. (2013) found that miR-21 is downregulated in BMSCs from estrogen deficiency-induced OP and promotes the osteogenic differentiation of BMSCs by targeting SPRY1.

Exogenous usage of glucocorticoids is the main risk factor for nontraumatic osteonecrosis of the femoral head (ONFH), which is termed as glucocorticoids associated ONFH and belongs to one type of steroid-associated osteonecrosis of the femoral head (SONFH). It had been demonstrated that miR-155-5p promotes osteogenic differentiation of BMSCs from SONFH by targeting glycogen synthetase kinase 3 beta (GSK-3β) and activating β-catenin signaling (Wu et al., 2021). Dai et al. (2019) reported that the expression level of miR-217 in BMSCs from patients with SONFH is decreased significantly, and miR-217 promotes the osteogenic differentiation of BMSCs by targeting DKK1. While miR-27a is downregulated in BMSCs from patients with SONFH. MiR-27a impairs the activation of phosphoinositide 3-kinase/protein kinase B/mammalian target of rapamycin (PI3K/AKT/mTOR) pathway by targeting PI3K, thereby reversing the inhibitory effect of glucocorticoids on osteogenic differentiation of BMSCs (Tang et al., 2021).

#### 2.2.3 The Regulation of miRNAs in Bone Regeneration and Bone Tissue Engineering

Si(OH)_4_ inhibits nuclear factor kappa B (NF-κB) by inducing the expression of miR-146a that activates RUNX2 expression to promote osteogenic differentiation of BMSCs (Zhou et al., 2016), suggesting miR-146a upregulation as a possible approach to promote the bone regenerative potential of BMSCs. Overexpression of miR-200c activates the AKT/β-catenin signaling pathway by targeting myeloid differentiation factor 88 (MYD88), which promotes the osteogenic differentiation of BMSCs (Xia et al., 2019). Similarly, miR-200c was found to promote the osteogenic differentiation of BMSCs *in vitro* by targeting sex-determining region Y-box 2 (SOX2)-mediated Wnt signaling and KLF4 (Akkouch et al., 2019). SOX2 is a major transcription factor affecting stem cell differentiation (Ma et al., 2014). Moreover, Hong et al. (2016) showed that polyethylenimine nanoparticle-based delivery of miR-200c improves the osteogenic differentiation of BMSCs and promotes bone regeneration. MiR-21 delivered by chitosan/hyaluronic acid nanoparticles promotes the osteogenesis of BMSC sheets (Zhao et al., 2016). These findings indicate the possible applications of nanomaterial-based exogenous miRNAs delivery for bone regeneration. Thus, the increased expression of osteogenesis promoting miRNAs has the potential application in bone repair. The regulatory function and mechanism of miRNAs-induced osteogenesis in BMSCs are summarized in Figure 2 and Table 1.

### 2.3 Mechanisms Involved in miRNAs-Inhibited Osteogenic Differentiation of BMSCs

#### 2.3.1 The Regulation of miRNAs in Physiological Conditions

**FIGURE 3:**
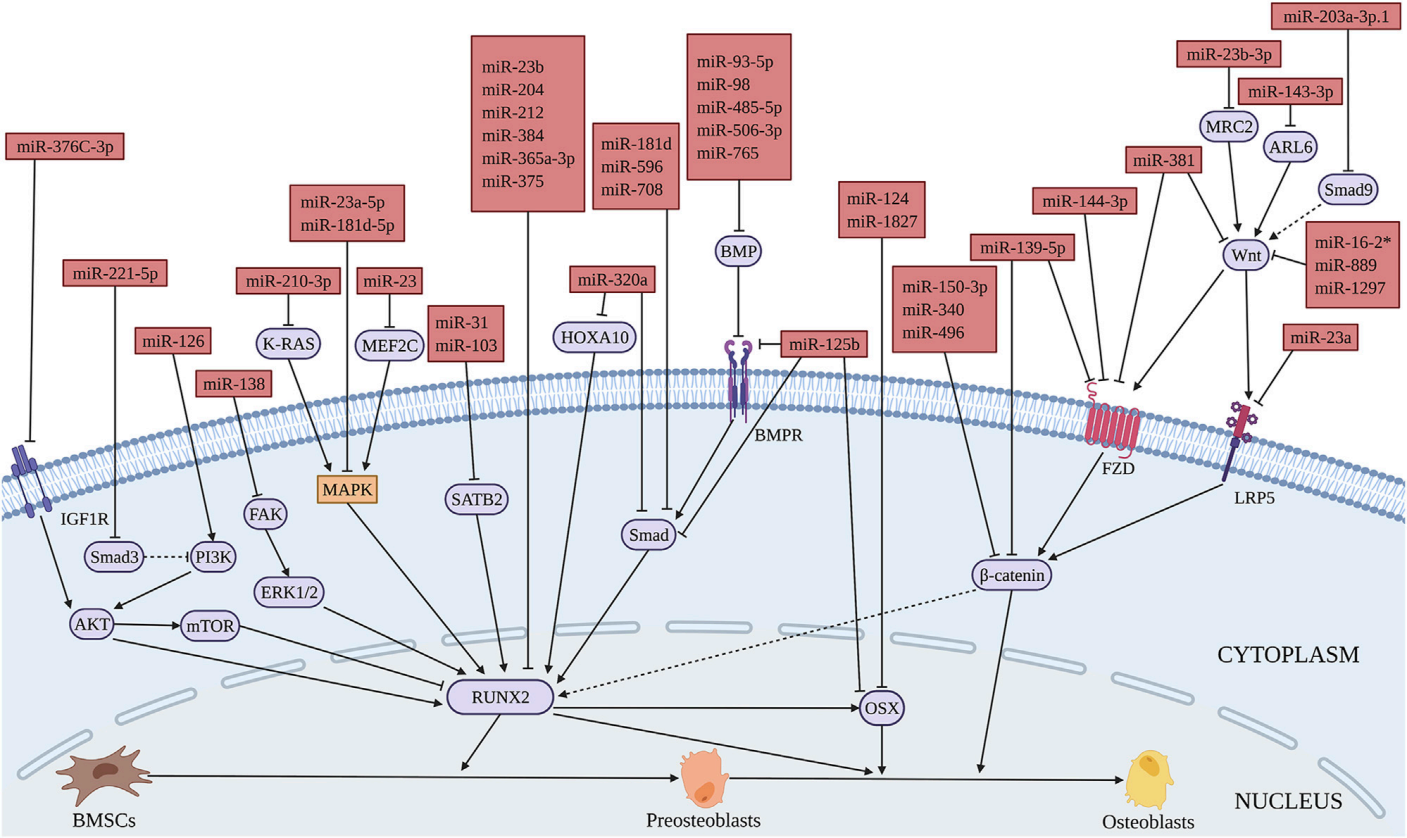
Illustration of the role and regulatory mechanism of miRNAs-inhibited osteogenic differentiation of BMSCs. (Created with BioRender.com).

Reports from literature had shown the inhibitory role of various miRNAs in the osteogenic differentiation of BMSCs. Low-density lipoprotein receptor-related protein 5 (LRP5) is an important Wnt receptor and plays an important role in Wnt/β-catenin signaling pathway. Li et al. found that miR-23a decreases the osteogenic differentiation of BMSCs by targeting LRP5 (Wang et al., 2016) (Figure 3 and Table 2). MiR-98 inhibits the osteogenic differentiation of BMSCs by targeting BMP2 (Zhang et al., 2017). Furthermore, miR-145 inhibits the osteogenic differentiation of BMSCs by targeting semaphorin 3A (SEMA3A) (Jin et al., 2020). Retinol (vitamin A) is a micronutrient essential for cell proliferation and differentiation. Its metabolite, retinoic acid, can promote osteoblast differentiation together with BMP2 (Skillington et al., 2002). MiR-223 regulates retinol metabolism by directly inhibiting the expression of retinoic acid-inducible dehydrogenase reductase 3 (DHRS3), reducing the osteogenic differentiation of BMSCs (Zhang et al., 2018). MiRNAs mediate the osteogenic differentiation processes of drugs, factors, etc. Overexpression of miR-625-5p reverses the promoting effect of quercetin on osteogenic differentiation of BMSCs (Bian et al., 2021). Wang et al. (2016) showed that miR-150-3p targets β-catenin and inhibits tumor necrosis factor-α (TNF-α) induced osteogenic differentiation of BMSCs, which inhibits the inflammation response during bone formation. Lin et al. showed that interleukin-1β (IL−1β) inhibits osteogenic differentiation of BMSCs *via* miR-496-mediated inhibition of β-catenin signaling. This study claimed miR-496 as a possible target to treat inflammation-related bone loss (Huang and Chen, 2017). MiR-143-3p is involved in cadmium suppression of the Wnt/β-catenin pathway and inhibits osteogenic differentiation of BMSCs by targeting adenosine diphosphate-ribosylation factor-like protein 6 (ARL6) (Wu et al., 2020). Therefore, miR-143-3p could be targeted to treat cadmium poisoning-related bone loss. MiR-153 is a mechanosensitive miRNA that inhibits the osteogenic differentiation of BMSCs by directly targeting BMP receptor (BMPR) 2 (Cao et al., 2015).

**TABLE 2:** MiRNAs that inhibit the osteogenic differentiation in BMSCs and underlying mechanisms.

miRNA	Study model	Signaling pathway	Effect	References
miR-9-5p	BMSCs *in vitro*	Targets DDX17	Involves in high glucose-mediated downregulation of COL1, OCN, OPN, and RUNX2	He et al. (2021)
miR-10a-5p	BMSCs *in vitro*	——	Inhibits the expression of ALP and RUNX2, as well as bone formation	Yingjie Zhang et al. (2020)
miR-16–2*	BMSCs from OP patients	Targets WNT5A to interfere Wnt signaling	Inhibits the expression of RUNX2, OSX, ALP, OCN, OPN, and COL1A1	Duan et al. (2018)
miR-23	BMSCs from healthy donors and OP patients	Targets MEF2C to regulate p38/MAPK signaling pathway	Inhibits the expression of RUNX2, OSX, ALP, and OCN	Jiang et al. (2020)
miR-23a	BMSCs from human iliac bone with jaw cysts	Targets CXCL12	Inhibits the expression of ALP, COL1, and RUNX2	Zhuang and Zhou, (2020)
	BMSCs *in vitro*	Targets LRP5 to inhibit Wnt/β-catenin signaling pathway	Inhibits the expression of RUNX2, ALP, and OPN	Nan Wang et al. (2016)
	BMSCs *in vitro*	——	Inhibits the expression of ALP, OPN, and RUNX2	Li et al. (2014)
miR-23a‐5p	BMSCs *in vitro*	Targets MAPK13 to regulate p38MAPK signaling pathway	Inhibits the expression of RUNX2, ALP, and OPN	Ren et al. (2018)
miR-23b	BMSCs *in vitro*	Targets RUNX2	Reduces ALP activity and calcium deposition leads to bone loss and inhibits bone formation	Deng et al. (2018)
miR-23b-3p	BMSCs from healthy donors and PMOP patients	Targets MRC2 to inhibit Wnt/β-catenin signaling	Inhibition of miR-23b-3p promotes expression of RUNX2, OSX, and OCN	Ran Li et al. (2021)
miR-29b-1-5p	BMSCs *in vitro*	Regulate SDF-1/CXCR4 axis	Inhibits the expression of COL1A1, RUNX2, OCN, and BMP2	Eisa et al. (2021)
miR-30d-5p	BMSCs from healthy donors and PMOP patients	——	Inhibits the expression of RUNX2	Zhi-Hao Wu et al. (2018)
miR-31	BMSCs from patients with ethanol-induced osteonecrosis	Targets SATB2	Inhibits the expression of BMP2, RUNX2, OSX, OCN, and OPN	Yu et al. (2019)
miR-93–5p	BMSCs from femoral neck fracture and ONFH patients	Targets BMP2	Inhibits the expression of ALP, OPN, RUNX2, and OSX	Ying Zhang et al. (2017)
miR-98	BMSCs *in vitro*	Targets BMP2	Inhibits the expression of RUNX2, ALP, and OCN	Guo-Ping Zhang et al. (2017)
miR-103	BMSCs *in vitro*	Targets SATB2	Inhibits the expression of RUNX2 and OCN	Lv et al. (2020)
miR-124	BMSCs *in vitro*	Targets OSX	Inhibits the expression of RUNX2 and OCN, and ALP activity	Jia-Zhen Tang et al. (2019)
miR-124	BMSCs *in vitro* and ectopic bone formation model	Targets DLX2, DLX3 and DLX5	Inhibits bone formation *in vivo*	Qadir et al. (2015)
miR-125b	BMSCs *in vitro* and femoral defect in nude mice	Targets BMPR1b	Inhibits the expression of RUNX2, OSX, and OCN, miR-125b inhibitor promotes bone formation *in vivo*	Huaqing Wang et al. (2017)
miR-125b	BMSCs from healthy donors and OP patients	Targets OSX	Inhibits the expression of RUNX2, ALP, COL1A1, and OCN	Chen et al. (2014)
miR-125b	BMSCs *in vitro*	Targets Smad4	Inhibits the expression of Smad4	Lu et al. (2013)
miR-126	BMSCs *in vitro*	Regulates PI3K/AKT and MEK1/ERK1 signaling pathways	Inhibits the expression of ALP, OPN, and RUNX2	Kong et al. (2020)
miR-133	BMSCs from healthy donors and OP patients	Targets SLC39A1	Inhibits the expression of ALP, RUNX2, and OSX	Zhang et al. (2015)
miR-135b	BMSCs from healthy donors and MM patients	Targets Smad5	Inhibits the ALP activity	Xu et al. (2013)
miR-138	BMSCs *in vitro* and ectopic bone formation in NOD/SCID mice	Targets FAK and regulates FAK downstream signaling	Inhibits the expression of RUNX2, OSX, ALP, and OCN, and bone formation *in vivo*	Eskildsen et al. (2011)
miR-138–5p	BMSCs *in vitro*	Targets FOXC1	——	Lan Zhang et al. (2021)
miR-139–5p	BMSCs *in vitro*	Targets CNNB1 and FZD4 to regulate Wnt/β-catenin pathway	Inhibits the expression of ALP, RUNX2, COL1, and OCN	Long et al. (2017)
miR-143–3p	BMSCs *in vitro*	Targets ARL6 to down-regulate Wnt/β-catenin pathway	Inhibits the expression of ALP and RUNX2	Lu Wu et al. (2020)
miR-144–3p	BMSCs from healthy controls and aplastic anemia patients	Targets TET2	Inhibits the expression of ALP and OCN	Haojie Wi et al. (2020)
miR-144–3p	BMSCs from healthy donors and ONFH patients	Targets FZD4	Inhibits the expression of RUNX2 and COL1A1	Sun et al. (2020b)
miR-145	BMSCs *in vitro*	Targets SEMA3A	——	Yucui Jin et al. (2020)
miR-150–3p	BMSCs *in vitro*	Targets β-catenin	Inhibits the activity of ALP, calcium contents, and the expression of RUNX2 and OSX	Nan Wang et al. (2016)
miR-153	BMSCs from young donors and elderly OP patients	Targets BMPR2	Inhibits the expression of ALP, OCN, and COL1A1	Cao et al. (2015)
miR-181d	BMSCs from femoral neck fracture and ONFH patients	Targets Smad3	Inhibits the expression of RUNX2 and Smad3	Xie et al. (2018)
miR-181d-5p	BMSCs *in vitro*	Targets MAPK1	Inhibits RUNX2 and OSX expression as well as ALP activity, and bone formation on a rough titaum surface	Yanping Liu et al. (2021)
miR-200a-3p	Blood from healthy donors and OP patients	Targets GLS	Inhibits the expression of OCN, RUNX2 and OPN	Lv et al. (2019)
miR-203a-3p.1	BMSCs from healthy donors and multiple MM patients	Targets Smad9 to inhibit WNT3A/β-catenin signaling pathway	Inhibits expression of ALP, OPN and OCN	Fan et al. (2019a)
miR-204	BMSCs *in vitro*	Targets RUNX2	Inhibits the expression of BMP2	Zhao et al. (2014)
miR-206	BMSCs *in vitro*	Targets GLS	Inhibits the expression of RUNX2 and OPN	Ying Chen et al. (2019)
miR-210–3p	BMSCs from healthy donors and OP patients	Targets K-RAS and downstream MAPK signaling activation	Inhibits the expression of ALP, OCN, RUNX2, and OSX	Hu et al. (2021)
miR-212 and miR-384	BMSCs *in vitro*	Targets RUNX2 and regulate OPG/RANKL pathway	Inhibit OSX expression and ALP activity	Yun Zhang et al. (2020)
miR-221–5p	BMSCs from healthy donors and MBD patients	Targets Smad3 and inhibits PI3K/AKT/mTOR pathway	Inhibits the expression of ALP, OPN, and OCN	Fan et al. (2019b)
miR-223	BMSCs *in vitro*	Targets DHRS3	miR-223 antagomir upregulates the expression of RUNX2, OPN and OCN	Shijie Zhang et al. (2018)
miR-223–3p	BMSCs *in vitro*	Targets FOXO3	Inhibits the expression of ALP, RUNX2, OCN, and Smad4	Long et al. (2021)
miR-320a	BMSCs *in vitro*	Targets HOXA10	Inhibits the expression of RUNX2, ALP, and OCN	Huang et al. (2016)
miR-320a	BMSCs *in vitro*	Targets Smad5	Inhibits the expression of OCN, OPN, and RUNX2	J-L Wang et al. (2020)
miR-337	BMSCs *in vitro*	Targets RAP1A	Inhibits the expression of RUNX2, ALP, OCN, OPN, and BMP2	Shuai Liu et al. (2021)
miR-340	BMSCs *in vitro*	Targets β-catenin	Inhibits the expression of OSX and RUNX2, and ALP	Du et al. (2017)
miR-346–5p	BMSCs *in vitro*	Targets transmembrane protein 9	Inhibits the expression of OSX and RUNX2, and decreases ALP activity and calcium deposition	Yicai Zhang et al. (2020)
miR-365a-3p	BMSCs *in vitro* and blood from healthy donors and OP patients	Targets RUNX2	Inhibits the expression of OCN, OPN, and COL1	Cheng et al. (2019)
miR-375	BMSCs *in vitro* and blood from healthy donors and OP patients	Targets RUNX2	Inhibits the expression of ALP, OCN, and RUNX2	Lei et al. (2019)
miR-376c-3p	BMSCs *in vitro*	Targets IGF1R and negatively regulate IGF1R/AKT signaling	Inhibits the expression of RUNX2, OPN, and OCN	Camp et al. (2018)
miR-381	BMSCs *in vitro*	Targets WNT5A and FZD3 to inhibit Wnt signaling pathway	Inhibits the expression of RUNX2, ALP, and COL1	Long et al. (2019)
miR-496	BMSCs *in vitro*	——	Inhibits the expression of OSX and RUNX2, and ALP activities	Huang and Chen, (2017)
miR-506–3p	BMSCs *in vitro* and bone tissues from healthy donors and OP patients	Targets BMP7	Inhibits the expression of OCN, OPN, and RUNX2	Jun Li et al. (2021)
miR-579–3p	Blood from healthy donors and OP patients	Targets SIRT1	Inhibits the expression of ALP, and RUNX2	B Luo et al. (2019)
miR-596	BMSCs from femoral neck fracture and SONFH patients	Targets Smad3	Inhibits the expression of ALP, OPN, RUNX2, and OSX	Fu et al. (2020)
miR-625–5p	BMSCs *in vitro*	——	Inhibits the expression of BMP2, OCN, and RUNX2, as well as ALP activity	Bian et al. (2021)
*miR-708*	BMSCs from GC-induced ONFH patients and ONFH patients after a previous fracture of the femoral neck	Targets Smad3 to regulate TGF-β signaling pathway	Inhibits the expression of Smad3 and RUNX2	Hao et al. (2016)
miR-765	BMSCs *in vitro*	Targets BMP6 to inhibit BMP6/Smad1/5/9 signaling	Inhibits the expression of RUNX2 and OCN	J-L Wangang et al. (2020)
miR-889	BMSCs from healthy donors and OP patients	Targets WNT7A and inhibit Wnt/β-catenin signaling pathway	Inhibits the expression of ALP, BMP2, RUNX2, OPN, and OCN	Xu et al. (2019)
miR-1271–5p	BMSCs *in vitro*	Targets FOXO1	Inhibits the expression of RUNX2, ALP and OCN	Qining Yang et al. (2021)
miR-1297	BMSCs and blood from healthy donors and OP patients	Targets WNT5A and affect Wnt signaling pathway	Inhibits the expression of RUNX2, OSX, ALP, OCN, OPN, and COL1A1	Q Wang et al. (2019)
miR-1827	BMSCs *in vitro*	Targets OSX	Inhibits the expression of OSX, OPN, COL1A, and OCN	Liu Liu et al. (2020)

#### 2.3.2 The Regulation of miRNAs in Pathological Conditions

MiR-23, miR-16-2*, miR-210-3p, miR-889 were found to be upregulated in bone tissues or BMSCs from OP patients. MiR-23 overexpression significantly inhibits the osteogenic differentiation of BMSCs by targeting MEF2C through the MEF2C/MAPK signaling pathway, thus accelerating OP development (Jiang et al., 2020). MiR-16-2* could interfere with Wnt signal transduction by targeting WNT5A to inhibit osteogenic differentiation of BMSCs (Duan et al., 2018). Furthermore, miR-210-3p inhibits the osteogenic differentiation of BMSCs by targeting Kirsten rat sarcoma viral oncogene (K-RAS) and the downstream MAPK signal (Hu et al., 2021). MiR-889 reduces the osteogenic capability of BMSCs by targeting WNT7A through the Wnt/β-catenin signaling pathway (Xu et al., 2019). In BMSCs of age-associated OP, miR-29b-1-5p significantly downregulates the expression of stromal cell-derived factor 1 (CXCL12)/C-X-C chemokine receptor type 4 (SDF-1(CXCL12)/CXCR4) axis as well as BMP2 and RUNX2, thus negatively regulating the osteogenic differentiation of BMSCs (Eisa et al., 2021). The levels of miR-200a-3p, miR-365a-3p, miR-579-3p, and miR-1297 are increased significantly in the serum of OP patients (Cheng et al., 2019; Luo et al., 2019; Lv et al., 2019; Wang et al., 2019). MiR-365a-3p decreases the osteogenic differentiation of BMSCs by targeting RUNX2 and promotes the progress of OP (Cheng et al., 2019). Sirtuin1 (SIRT1) is an important regulator of Wnt signaling pathway, which promotes the expression of downstream differentiation related factors by de-acetylating β-catenin, thus regulating the differentiation of MSCs (Simic et al., 2013). MiR-579-3p inhibits the osteogenic differentiation of BMSCs by targeting SIRT1 (Luo et al., 2019). MiR-1297 overexpression interferes with the regulation of the Wnt signaling pathway by targeting WNT5A, thereby inhibiting the osteogenic differentiation of BMSCs (Wang et al., 2019). MiR-375 was shown to be increased in the serum of OP patients. Polypeptide drug teriparatide promotes osteogenic differentiation of BMSCs through decreasing miR-375, while the increased expression of miR-375 reverses this process (Lei et al., 2019). Forkhead box (FOX) O1 belongs to the forkhead family and is a key transcription factor regulating cell physiological function, including osteoblasts (Kitamura et al., 2005; Kim et al., 2012). Yang et al. (2021). reported a higher expression of miR-1271-5p, which is higher in osteoporotic trabecular bone tissues, and inhibits the osteogenic differentiation of BMSCs by downregulating its target FOXO1 as well as the expression of RUNX2, ALP, and OCN. In addition, miR-133 expression is significantly enhanced in BMSCs from PMOP patients. Solute carrier family 39 member one (SLC39A1) encodes zinc transporter 1, which plays an important role in the initiation of MSCs osteogenic lineage (Tang et al., 2006). MiR-133 regulates the osteogenic differentiation of BMSCs by inhibiting SLC39A1 expression (106). It has been demonstrated that diabetes increases the risk of OP (Chau et al., 2003). MiR-337 negatively regulates osteogenic differentiation of BMSCs by targeting ras-related protein 1A (RAP1A) under hyperglycemic conditions (Liu et al., 2021). Dead-box helicase 17 (DDX17) regulates the RUNX2 expression in osteoblast differentiation (Fuller-Pace and Ali, 2008), and miR-9-5p knockout promotes the osteogenic differentiation of BMSCs through targeting DDX17 under hyperglycemic conditions (He et al., 2021).

The role of miRNAs in ONFH-related diseases is also reported. Zhang et al. (2017) found that miR-93-5p is upregulated in the peripheral blood of trauma-induced ONFH patients, which inhibits osteogenic differentiation of BMSCs by targeting BMP2. MiR-181d and miR-596 are upregulated in the bone marrow of SONFH patients, while miR-708 is increased in BMSCs. These miRNAs inhibit the osteogenic differentiation of BMSCs by targeting Smad3, thereby promoting the progression of SONFH (Hao et al., 2016; Xie et al., 2018; Fu et al., 2020). Furthermore, miR-144-3p was found to be downregulated in BMSCs from patients with SONFH, and inhibit the osteogenic differentiation of BMSCs by targeting frizzled (FZD) 4 (Sun et al., 2020b). Special AT-rich sequence-binding protein 2 (SATB2) is a key regulator involved in gene expression and chromatin remodeling. SATB2 overexpression can induce the differentiation of pluripotent stem cells *in vitro* and significantly enhance bone regeneration and bone repair *in vivo* (Zhou. et al., 2016). In BMSCs from ethanol-induced osteonecrosis, the expression of miR-31 is increased. MiR-31 inhibits the osteogenesis of BMSCs by targeting SATB2 (Yu et al., 2019). Thus, the expression of these miRNAs could be inhibited in BMSCs to treat ONFH.

In addition, miRNAs play an important role in hematologic diseases. The expression of miR-203a-3p.1 is significantly decreased in BMSCs from patients with multiple myeloma (MM). MiR-203a-3p.1 inhibits the osteogenic differentiation of BMSCs by directly targeting Smad9 through the WNT3A/β-catenin signaling pathway (Fan et al., 2019a). MiR-135b is abnormally upregulated in BMSCs from MM patients. Mechanistically, miR-135b directly targets Smad5 and negatively regulates its expression, finally inhibiting the osteogenic differentiation of BMSCs (Xu et al., 2013). Myeloma bone disease (MBD) is one of the clinical features of MM. Aggressive osteolysis and low bone mass phenotype are frequently observed in MBD patients. Fan et al. (2019b) showed that miR-221-5p inhibition significantly promotes the osteogenic differentiation of BMSCs from MBD patients by targeting Smad3 and activating the PI3K/AKT/mTOR signaling pathway. Inhibition of these miRNAs in bone marrow might prevent MBD-induced bone loss. Ten-eleven translocation (TET) family is an important epigenetic modifier, which can demethylate DNA and play a key role in stem cell differentiation (Dawlaty et al., 2014; Su et al., 2019). MiR-144-3p inhibits osteogenic differentiation of BMSCs of patients with aplastic anemia (AA) by inhibiting TET2 (Li et al., 2020). Furthermore, miR-204 inhibits the osteogenic differentiation of BMSCs from AA by directly inhibiting RUNX2 (Zhao et al., 2014).

#### 2.3.3 The Regulation of miRNAs in Bone Regeneration and Bone Tissue Engineering

BMSCs infected with these miRNAs sponges may be used in regenerative medicine. Titanium surface modification can change the shape and activity of MSCs, promote the differentiation of these cells into osteoblast lineage and upregulate osteogenic genes. MiR-23a inhibits the osteogenic differentiation of BMSCs on the surface of titanium nanotubes by targeting CXCL12 (Zhuang et al., 2019). Furthermore, the micro-arc oxidation surface of titanium implant promotes osteogenic differentiation by activating ERK1/2-miR-1827-OSX, while the overexpression of miR-1827 significantly inhibits the osteogenic differentiation of BMSCs (Liu et al., 2020). MiR-181d-5p regulates the implants’ surface roughness-induced osteogenesis. Inhibition of miR-181d-5p enhances osteogenic differentiation of BMSCs by targeting MAPK1 (Liu et al., 2021). Furthermore, the addition of miR-23a and miR-1827 inhibitors in BMSCs with titanium may increase bone integration. MiR-138 inhibits osteogenic differentiation of BMSCs by targeting focal adhesion kinase (FAK) signaling, thus reducing the ectopic bone formation of BMSCs *in vivo* by the combination of hydroxyapatite/tricalcium phosphate (HA/TCP) scaffolds (Eskildsen et al., 2011). MiR-125b inhibits osteogenic differentiation of BMSCs by targeting BMPR1b. The application of demineralized bone matrix with BMSCs treated with miR-125b inhibitor could be used to repair bone defects *in vivo* (Wang et al., 2017). BMSC transfected with miR-124 combined with HA/TCP scaffolds were subcutaneously transplanted into nude mice, demonstrating the inhibitory effect of miR-124 on the formation of ectopic bone *in vivo* (Qadir et al., 2015). Thus, sponges of these inhibitory miRNAs on osteogenic differentiation may be used in bone regeneration and bone repair. The regulatory function and mechanism of miRNAs that inhibit osteogenesis in BMSCs are summarized in Figure 3 and Table 2.

## 3 LncRNAs and Osteogenic Differentiation of BMSCs

### 3.1 The Biogenesis and Function of lncRNAs

LncRNAs are a group of ncRNAs with a length >200 nucleotides. According to their gene structure and the position relationship with protein-coding genes, lncRNAs are categorized into five groups: (Gaihre et al., 2017): long intergenic ncRNAs, which are located between coding genes, (Li et al., 2018), intronic lncRNAs, which originate from the intronic region of coding genes, (Nicot et al., 2020), antisense lncRNAs, which share same sequences with coding mRNA on the non-coding strand genes, (Tessier et al., 2005), bidirectional RNA, which possess the same transcription start sites with coding genes, and (Younger and Chapman, 1989) sense RNAs, which overlap with coding mRNAs on the coding strand of genes (Ponting et al., 2009; McCabe and Rasmussen, 2021). Also, lncRNAs could be divided depending on their functions and regulatory mechanisms as decoy lncRNAs, guide lncRNAs, scaffold lncRNAs, stabilizing lncRNAs, and competitive endogenous lncRNAs (ceRNAs) (McCabe and Rasmussen, 2021). LncRNAs can derive from diverse sequences of genes, including their own sequences and other promoter sequences, as well as the enhancer sequences. The biogenesis of lncRNAs differs with cell type and cell stage (Jiang and Zhang, 2021). Sharing a similar biogenesis process to mRNAs, lncRNAs are transcribed by RNA polymerase II and then capped at the 5′ region, polyadenylated at the 3′ region, and spliced (Goff and Rinn, 2015). In addition, they are expressed in a specific spatial and temporal manner influencing their functions. Recent studies demonstrate the existence of a small open reading frame in lncRNAs, which indicates their potential in various cellular processes (Ji et al., 2015).

### 3.2 LncRNAs Promote the Osteogenic Differentiation of BMSCs

LncRNAs are now known to exert influence on diverse biological processes, such as cell cycle (Guiducci and Stojic, 2021), proliferation (Liu et al., 2021), metastasis (Hong et al., 2021), and differentiation (Li. et al., 2021), as well as several diseases (Li et al., 2021; Liu et al., 2021; Xin and Liu, 2021). Moreover, emerging evidence shows that lncRNAs participate in the osteogenic differentiation of BMSCs. During osteogenic differentiation, lncRNAs may play their biological functions via four major ways including serving as miRNAs sponges or precursors (Huang et al., 2015), modulating epigenetic modification (Huo et al., 2017), and mediating other regulatory mechanisms.

#### 3.2.1 The Regulation of lncRNAs in Physiological Conditions

**FIGURE 4:**
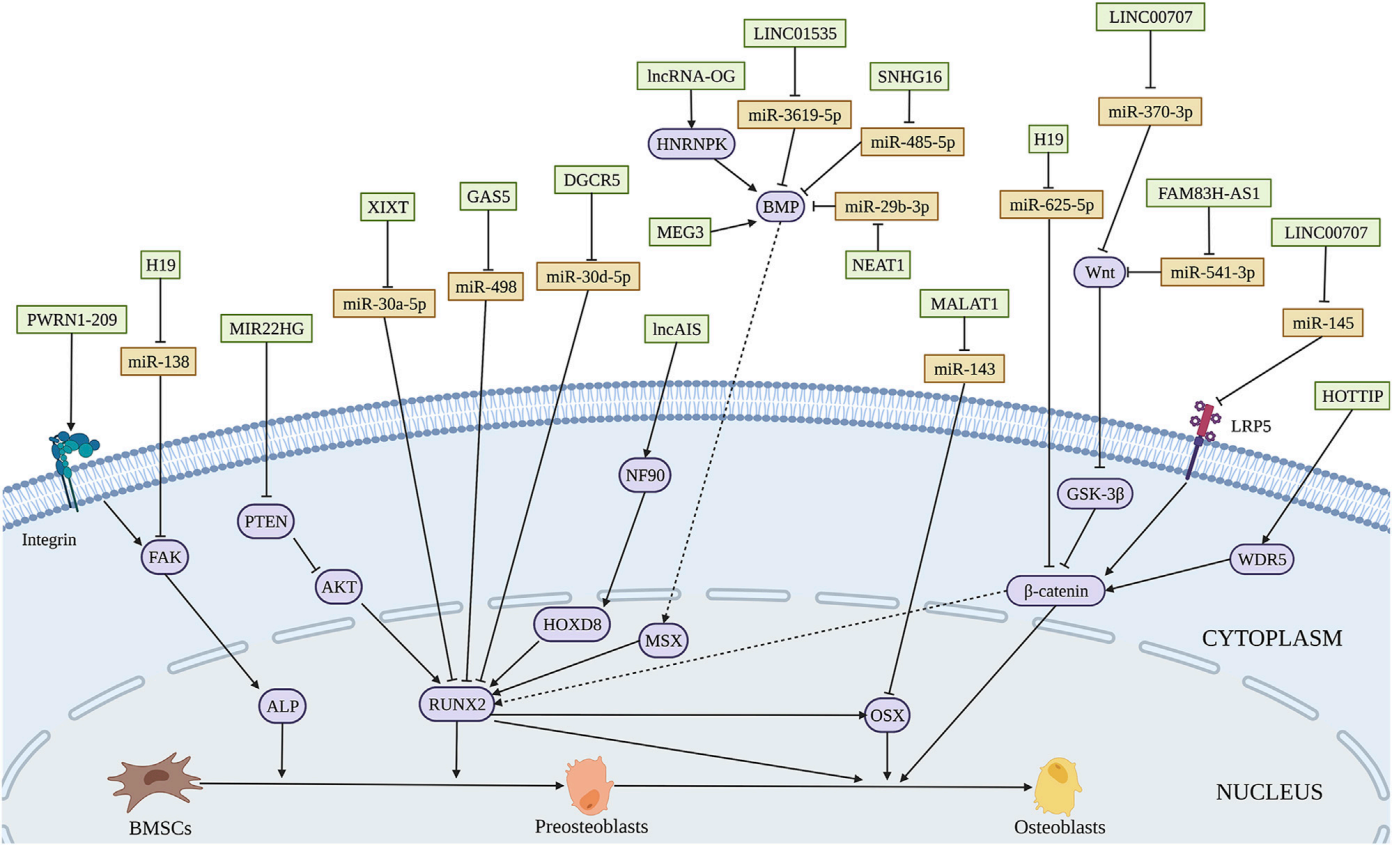
Illustration of the role and regulatory mechanism of lncRNAs-induced osteogenic differentiation of BMSCs. Various lncRNAs promote BMSCs osteogenesis through sponging miRNA (orange), activating epigenetic regulation, and mediating other regulatory mechanisms. (Created with BioRender.com).

Several lncRNAs are involved in promoting osteogenesis through direct interaction with miRNAs. H19 is one of the most studied lncRNA in osteogenic differentiation. The increased expression of H19 during fetal development indicates its highly conserved characteristic throughout evolution. H19 not only influences various biological processes such as RNA progression, and cellular proliferation but also implicates in multiple human disorders (Shermane Lim et al., 2021; Wang and Qi, 2021). Quercetin has been proved to affect osteogenesis and osteoclastgenesis by regulation of a number of mechanisms, including mediating the expression of osteoprotegerin, and MAPK signaling (Wong et al., 2020). Quercetin also plays a significant role in accelerating osteogenesis via interaction with H19 by sponging miR-625-5p, and ultimately activates Wnt/β-catenin pathway (Bian et al., 2021) (Figure 4 and Table 3). Bi et al. (2020) found that H19 expression is increased in a time-dependent manner during osteogenesis. Further studies elucidated that H19 promotes osteogenic differentiation *via* miR-140-5p/SATB2 axis in BMSCs. Besides, H19 binds to miR-138, an miRNA targeting the gene encoding FAK called PTK2, thus upregulates downstream FAK expression, playing an important role in mechanical tension-induced osteogenic differentiation of BMSCs (Wu et al., 2018). Cai et al. (2020) found that LINC00707 is increased during osteogenic differentiation. Further studies demonstrated that LINC00707 modulates LRP5 expression by sponging miR-145, which activates the Wnt/β-catenin pathway and promotes the osteogenic differentiation of BMSCs. LINC01535 contributes to the osteogenic process via acting as a sponge of miR-3619-5p to alter BMP2 expression (Zhao et al., 2020). Similarly, lncRNA NEAT1 binds with miR-29b-3p which targets BMP1 to accelerate the osteogenic process (Zhang et al., 2019).

**TABLE 3:** LncRNAs that promote osteogenic differentiation of BMSCs and underlying mechanisms.

LncRNA	Study model	Signaling pathway	Effect	References
SNHG14	BMSCs from the femoral head of patients with or without OP receiving THA	Targets miR-185–5p/WISP2 to activate Wnt/β-catenin signaling	Promotes expression of ALP, OCN, and OPN	Z H Liu et al. (2021)
MALAT1	BMSCs from SONFH tissues and femoral neck fracture tissues	Targets miR-214 to regulate ATF4	Promotes expression of RUNX2, ALP, and OCN	Xian-Zhe Huang et al. (2020)
	BMSCs from femoral head tissues during THA with or without OP	Targets miR-143 to regulate OSX	Promotes expression of ALP, OCN, OPN, and OSX	Gao et al. (2018)
LINC00707	BMSCs *in vitro*	Targets miR-145/LRP5 to activate Wnt/β-catenin signaling	Promotes expression of ALP, OCN, RUNX2, and OSX	Cai et al. (2020)
	BMSCs *in vitro* and ectopic bone formation model	Targets miR-370–3p/WNT2B to activate Wnt/β-catenin signaling	Promotes expression of ALP, RUNX2, and OCN	Jia et al. (2019)
PWRN1-209	BMSCs *in vitro*	Activates integrin-FAK-ALP signaling	Promotes expression of ALP and COL1A1	Mingyue Wang et al. (2020)
H19	BMSCs *in vitro*	Targets miR-625–5p to activate Wnt/β-catenin signaling	Promotes expression of BMP2 and RUNX2	Bian et al. (2021)
	BMSCs in vitro	Regulates miR-140–5p/SATB2 axis	Promotes expression of COL1A1, RUNX2, OCN, and OPN	Bi et al. (2020)
	BMSCs *in vitro*	Regulates miR-138/FAK axis	Promotes expression of OPN, RUNX2, and OCN	Jiajing Wu et al. (2018)
LINC01535	BMSCs *in vitro*	Targets miR-3619–5p to activate BMP signaling	Promotes expression of OCN, OSX, and RUNX2	Yiwen Zhao et al. (2020)
XIXT	BMSCs from femoral head tissues during THA with or without OP	Targets miR-30a-5p to regulate RUNX2	Promotes expression of ALP and RUNX2	H-L Zhang et al. (2019)
NEAT1	BMSCs from femoral head tissues during THA with or without OP	Targets miR-29b-3p to activate BMP signaling	Promotes expression of ALP, OCN, and OPN	Yingzi Zhang et al. (2019)
SNHG16	BMSCs from healthy donors and OP patients	Targets miR-485–5p to activate BMP signaling	Promotes expression of ALP, OCN, and OPN	Asila et al. (2021)
GAS5	BMSCs from healthy donors and OP patients	Targets miR-498 to regulate RUNX2	Promotes expression of RUNX2	Feng et al. (2019)
DGCR5	BMSCs from healthy premenopausal women and PMOP patients	Targets miR-30d-5p to regulate RUNX2	Promotes ALP activity	Zhi-Hao Wu et al. (2018)
XIST	BMSCs from femoral head tissues during THA with or without OP	Targets miR-9-5p to regulate ALP	Promotes expression of OCN and OPN	Zheng et al. (2020)
FAM83H-AS1	BMSCs *in vitro*	Targets miR-541–3p/WNT3A to activate Wnt/β-catenin signaling	Promotes expression of RUNX2, OCN, and OSX	Haojie Wu et al. (2020)
MEG3	BMSCs from pediatric AA patients and healthy donors	Activates BMP signaling pathway	——	Huanhuan Li et al. (2021)
	BMSCs *in vitro*	Activates BMP signaling pathway	Promotes expression of RUNX2, ALP, OSX, and OCN	Chen et al. (2018)
MIR22HG	BMSCs *in vitro*	Targets PTEN to activate AKT signaling	Promotes expression of RUNX2, ALP, and OCN	Chanyuan Jin et al. (2020)
ENST00000563492	BMSCs from patients with bone nonunion or normal fracture healing; bone formation in nude mice	Targets miR-205–5p to regulate CDH11 and VEGF.	Promotes expression of COL1A1, RUNX2, and OCN	Ouyang et al. (2020)
HOTTIP	BMSCs from blood, and in ectopic bone formation	Targets WDR5 to activate Wnt/β-catenin signaling	Promotes expression of RUNX2, OSX, ALP, and OCN	Ruiduan Liu et al. (2020)
lncRNA-OG	BMSCs *in vitro* and ectopic bone formation model	Targets hnRNPK to activate BMP signaling	Promotes expression of RUNX2, ALP, OSX, and OCN	Su’an Tang et al. (2019)
lncAIS	BMSCs from AIS patients and healthy donors; ectopic bone formation	Interacts NF90 to enhance the mRNA stability of HOXD8	Promotes expression of ALP, RUNX2, LPL, and PPAR	Qianyu Zhuang et al. (2019)

LncRNAs could influence osteogenic differentiation of BMSCs by epigenetic regulation. WD Repeat-Containing Protein 5 (WDR5) is a transcription factor binding with the promoter of β-catenin. Upregulation of lncRNA HOX transcript at the distal tip (HOTTIP) promotes ectopic bone formation *in vivo*. The interaction of HOTTIP and WDR5 facilitates WDR5 translocation into the nucleus and β-catenin transcription, thus increasing osteogenic differentiation (Liu et al., 2020). Tang et al. (2019) used customized microarrays to reveal a novel lncRNA, osteogenesis-associated lncRNA (lncRNA-OG), which was upregulated by almost 12-fold during BMSCs osteogenesis. LncRNA-OG overexpression induces osteogenic differentiation of BMSCs *in vitro* and ectopic bone formation in nude mice. Mechanically, lncRNA-OG regulates BMP signaling pathway through direct interaction with heterogeneous nuclear ribonucleoprotein K (hnRNPK). Moreover, hnRNPK is associated with lncRNA-OG transcriptional activity by involving in the H3K27 acetylation of the lncRNA-OG promoter.

#### 3.2.2 The Regulation of lncRNAs in Pathological Conditions

LncRNAs dysregulation is widely associated with bone-related diseases. The dysfunction of osteogenesis plays a key role in SONFH. A decline in lncRNA MALAT1 expression was found in SONFH tissues (Huang et al., 2020). ATF4, a vital regulator in bone formation, transactivates numerous osteogenic genes like RUNX2, BSP, OSX (Chan et al., 2021). MALAT1 influences ATF4 expression through sponging miR-214, ultimately increasing osteogenesis (Huang et al., 2020). What’s more, the expression of MALAT1 is significantly lower in BMSCs from discarded femoral head tissues under THA with OP than that without OP. MALAT1 could elevate an essential osteogenesis-related gene OSX expression through miR-143, affecting the osteogenic process and the development of OP (Gao et al., 2018). LncRNAs are related to the development and therapy of OP. LncRNA XIXT is downregulated, while miRNA-30a-5p is upregulated in the serum of OP patients. Mechanistically, lncRNA XIXT promotes osteogenesis by serving as a sponge of miR-30a-5p to upregulate RUNX2 (Zhang et al., 2019). Aberrant expression of small nucleolar RNA host gene 16 (SNHG16) had been reported in BMSCs from OP patients. And the promoting effect of SNHG16 on the osteogenic differentiation of BMSCs is modulated by SNHG16/miR-485-5p/BMP7 axis (Asila et al., 2021). Feng et al. (2019) demonstrated that lncRNA GAS5 is downregulated in BMSCs isolated from OP patients. Osteoblastic differentiation is promoted by the regulatory effect of GAS5 on miR-498, leading to increased RUNX2 expression and alleviating the development of OP. DEP domain-containing mTOR interacting protein (DEPTOR) is the endogenous inhibitor of mTOR, which is crucial to osteogenic differentiation and involved in OP. DEPTOR binds with the promoter of lncRNA maternally expressed 3 (nonprotein coding) (MEG3) to inhibit its transcription, consequently inactivating BMP4 signaling to restrain the osteogenic differentiation of BMSCs. Further study showed that downregulation of DEPTOR contributes to bone formation *in vivo* (Chen et al., 2018). Downregulated in BMSCs from osteoporosis patients, lncRNA X inactivate-specific transcript (XIST) promotes osteoblast differentiation and represses OP by regulating miR-9-5p and increasing the expression of its target ALP (Zheng et al., 2020). Sharing similar expression pattern in BMSCs from patients with PMOP, lncRNA DGCR5 upregulates RUNX2 to induce osteogenic differentiation, by sponging miR-30d-5p, thus, beneficial to delaying PMOP development (Wu et al., 2018). It has proved that osteomyelitis impedes the differentiation of BMSCs. A decline in the expression of lncRNA FAM83H-AS1 was identified in BMSCs during staphylococcal protein A-induced osteomyelitis. Mechanically, FAM83H-AS1 improves osteogenic differentiation of BMSCs by serving as a ceRNA of miR-541-3p, which brings augmentation in the expression of WNT3A, a critical member of the Wu et al. (2020)signaling pathway. Besides, AA, a common hematological disease, is characterized by inhibition of osteoblastic differentiation. Lower expression of MEG3 was detected in BMSCs of AA patients. DNA cytosine-5-methyltransferase 1 is correlated with the hypermethylation of the MEG3 promoter. MEG3 increases the transcriptional activity of BMP4 and positively affects osteoblastic differentiation of BMSCs (Li et al., 2021). Decreased osteogenic capability of BMSCs exhibits in adolescent idiopathic scoliosis (AIS) patients. Zhuang et al. (2019) reported downregulation of novel lncAIS in BMSCs from AIS patients. The interplay between lncAIS and NF90 promotes HOXD8 mRNA stability and eventually promotes the osteogenesis in normal BMSCs *in vitro* and *in vivo*.

#### 3.2.3 The Regulation of lncRNAs in Bone Regeneration and Bone Tissue Engineering

LncRNAs, which have a promoting effect on osteogenic differentiation, are overexpressed in BMSCs. The modified BMSCs with beneficial lncRNAs loaded on biomaterials maybe used to repair bone defect *in vivo*. A novel lncRNA Prader-willi region ncRNAs 1–209 (PWRN1-209) was proved to enhance osteoblast differentiation on microtopography titanium surfaces possibly through integrin/FAK/ALP signaling (Wang et al., 2020). Acting as a ceRNA for miR-370-3p, LINC00707 influences osteogenic differentiation via the Wnt/β-catenin pathway *in vitro*. And LINC00707 modified BMSCs loaded on HA/TCP promote ectopic bone formation in NOD/SCID mice (Jia et al., 2019). Various studies have revealed the indispensable role of the PTEN/AKT pathway in bone formation (Nielsen-Preiss et al., 2003). Targeting PTEN/AKT pathway, lncRNA MIR22HG serves as a positive regulator of osteogenic differentiation in BMSCs *in vitro* and Bio-Oss mediated ectopic bone formation *in vivo* (Jin et al., 2020). Reports demonstrated that the poor osteogenic potential of BMSCs typifies bone nonunion (Tawonsawatruk et al., 2014). LncRNA ENST00000563492 functions as a sponge of miR-205-5p to elevate Cadherin-11 (CDH11) and vascular endothelial growth factor (VEGF) expression, enhancing osteogenesis of BMSCs *in vitro* and bone formation by a combination of matrigel *in vivo*. ENST00000563492 was considered a new therapeutic target for bone nonunion (Ouyang et al., 2020). The osteogenesis promoting lncRNAs may be further evaluated.

### 3.3 LncRNAs That Inhibit Osteogenic Differentiation in BMSCs

**FIGURE 5:**
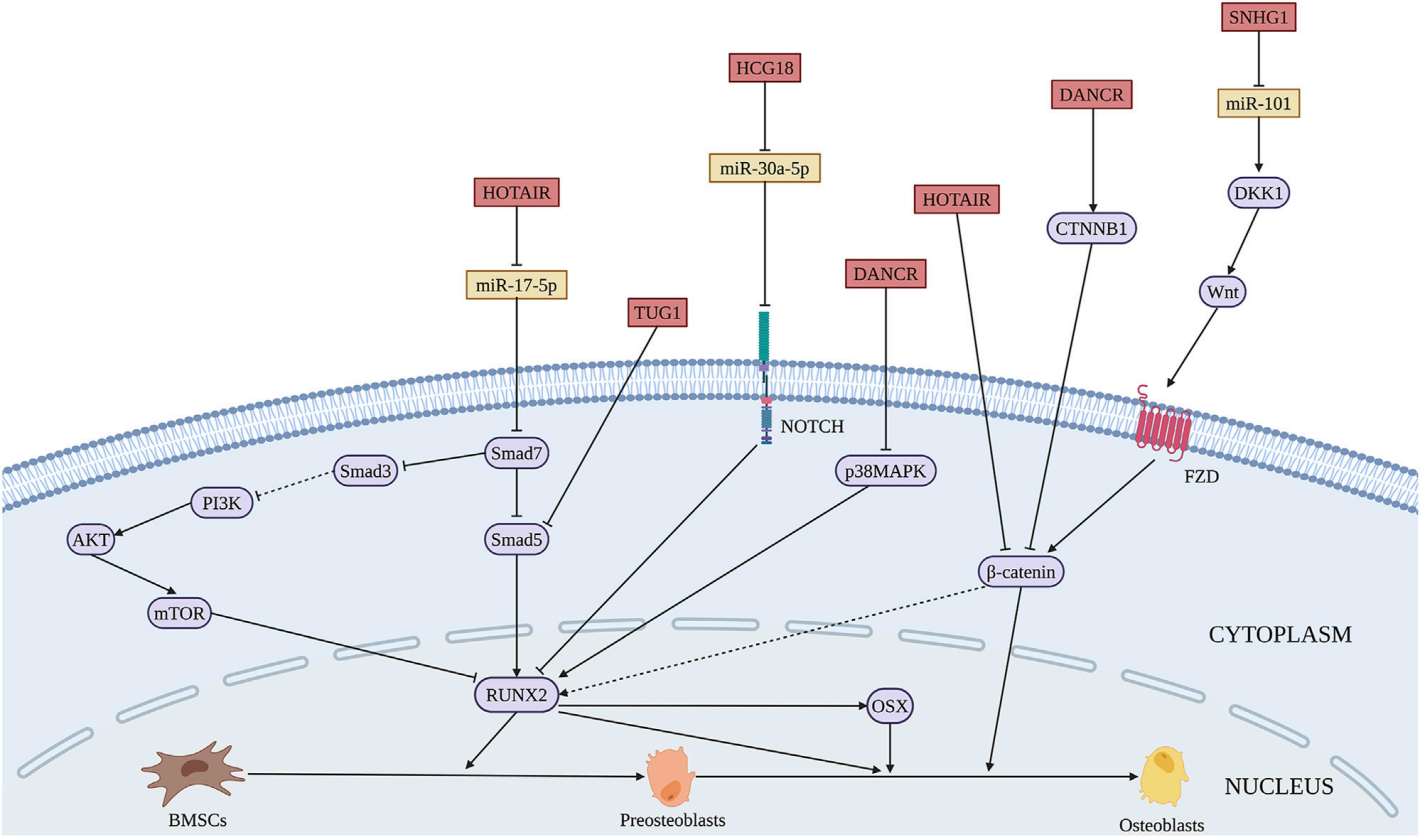
Illustration of the role and regulatory mechanism of lncRNAs-inhibited osteogenic differentiation of BMSCs. Some lncRNAs inhibit BMSCs osteogenesis through sponging pro-osteogenic miRNAs (orange). (Created with BioRender.com).

Likewise, multiple lncRNAs are involved in the suppression of osteogenic differentiation of BMSCs by sponging pro-osteogenic miRNAs. The expression of SNHG1 is decreased in a time-dependent manner during osteogenic differentiation. LncRNA SNHG1 inhibits osteogenesis via the miR-101/DKK1 axis and modulation of the Wnt/β-catenin signaling pathway by acting as a ceRNAs of miR-101 (Xiang et al., 2020) (Figure 5 and Table 4). LncRNA differentiation antagonizing non-protein coding RNA (DANCR) is downregulated during osteogenesis. DANCR inhibits osteogenesis of BMSCs as a sponge of miR-1301-3p which modulates prospero homeobox 1 (PROX1) expression (Weng et al., 2021). DANCR can also mediate cell proliferation and osteoblastic differentiation through the inactivation of the p38/MAPK pathway (Zhang et al., 2018). Inhibitory lnRNAs are related to bone diseases. Abnormal expression of lncRNA LOXL1 antisense RNA 1 (LOXL1-AS1) was found in peripheral blood from PMOP patients. LOXL1-AS1 suppresses osteogenic differentiation by mediating HMGA2 expression and subsequent C/EBPβ-mediated PPARγ expression by binding with miR-196a-5p in BMSCs from PMOP (Zhang et al., 2020). The expression of lncRNA MEG3 is upregulated in BMSCs of patients with PMOP. MEG3 regulates miR-133a-3p, accompanied by decreased SLC39A1 expression, to repress the osteogenic differentiation of BMSCs (Wang et al., 2017). The inhibitory effect of MEG3 on the osteogenesis of BMSCs is different from the previous reports (Chen et al., 2018; Li et al., 2021), which may be resulted from the BMSCs isolated from patients with different diseases. Increased in BMSCs from OP patients, lncRNA HCG18, which regulates miR-30a-5p/notch receptor 1 (NOTCH1) axis, suppresses osteogenic differentiation of BMSCs in OP patients and mice (Che et al., 2020). Wang et al. identified the aberrant expression of lncRNA DANCR, miR-320a, and CTNNB1 in BMSCs derived from OP patients. Furthermore, during the osteogenesis in BMSCs, DANCR and miR-320a regulate the Wnt/β-catenin signaling pathway through CTNNB1 inhibition, ultimately inhibiting the process (Wang et al., 2020). It has been suggested that the abnormal osteoblast differentiation of BMSCs is responsible for the pathogenesis of nontraumatic ONFH. In BMSCs of patients with nontraumatic ONFH, lncRNA HOX transcript antisense RNA (HOTAIR) expression was remarkably higher than normal. The sponging effect of HOTAIR on miR-17-5p leads to the decreased expression of Smad7, thus suppressing osteoblast differentiation of BMSCs in ONFH (Wei et al., 2017).

**TABLE 4:** LncRNAs that inhibit osteogenic differentiation of BMSCs and underlying mechanisms.

LncRNA	Study model	Signaling pathway	Effect	References
SNHG1	BMSCs *in vitro*	Targets miR-101/DKK1 to inactivate Wnt/β-catenin signaling	Inhibits expression of RUNX2, OCN, and OPN	Jie Xiang et al. (2020)
LOXL1-AS1	BMSCs *in vitro* and peripheral blood from PMOP patients or healthy donors	Regulates miR-196a-5p/HMGA2 axis	Inhibits expression of ALP, OPN, and RUNX2	Ling Zhang et al. (2020)
MEG3	BMSCs from healthy premenopause women and PMOP patients	Regulates miR-133a-3p/SLC39A1 axis	Inhibits expression of RUNX2, OCN, and OPN	Qiujun Wang et al. (2017)
DANCR	BMSCs *in vitro*	Regulates miR-1301–3p/PROX1 axis	Inhibits expression of ALP, RUNX2, OCN, and OSX	Weng et al. (2021)
	BMSCs *in vitro*	Inactivates p38/MAPK signaling pathway	Inhibits expression of OCN, COL1, and RUNX2	Jinlong Zhang et al. (2018)
	BMSCs from PMOP patients and healthy donors	Targets CTNNB1 to inactivate β-catenin signaling pathway	Inhibits expression of TCF-1, RUNX2, OPN, and OCN	Cheng-Gong Wang et al. (2020)
HOTAIR	BMSCs from non-traumatic ONFH and osteoarthritis patients	Targets miR-17–5p to inactivate Smad7	Inhibits expression of RUNX2, COLA1, and ALP	Wei et al. (2017)
	BMSCs from OP patients and healthy donors	Inactivates Wnt/β-catenin signaling pathway	Inhibits expression of ALP, RUNX2, and OCN	J-J Shen et al. (2019)
TUG1	BMSCs *in vitro*	——	Inhibits expression of RUNX2, and OGN	Weiwei Zhang et al. (2019)
ZBTB40-IT1	BMSCs *in vitro*	Inactivates Wnt signaling pathway	Inhibits expression of RUNX2, OSX, ALP, and COL1A1	Mei et al. (2019)
HCG18	BMSCs from femoral head tissues during THA with or without OP	Targets miR-30a-5p to activate NOTCH1 signaling	Inhibits expression of ALP, OCN, and OPN	Che et al. (2020)

Additionally, lncRNAs inhibit the osteogenic process via epigenetic regulation. LncRNA ZBTB40-IT1 exerts an adverse effect on osteogenic differentiation in the manner of modulating WNT4, a crucial gene of the Wnt signaling pathway, while ZBTB40 has the opposite function (Mei et al., 2019). HOTAIR is significantly upregulated in OP patients both in serum and BMSCs levels. It suppresses the differentiation of BMSCs into osteoblasts through the Wnt/β-catenin signaling pathway (Shen et al., 2019). Taurine Upregulated Gene 1 (TUG1), a notably increased lncRNA after irradiation, abolishes the Smad5 signaling using the reciprocal action with the 50–90 amino acid region of Smad5 and blocking the nuclear translocation of p-Smad5 that serves as a negative regulator of osteogenic differentiation (Zhang et al., 2019). Thus, silencing the expression of inhibitory lncRNAs may increase the application potential in bone regeneration.

## 4 CircRNAs and the Osteogenic Differentiation of BMSCs

### 4.1 The Biogenesis and Function of circRNAs

CircRNAs are a kind of covalently closed ncRNAs (Kristensen et al., 2019). Unlike linear RNAs, circRNAs are more stable due to the lack of 5′ to 3′ polarity and polyadenylated tail. CircRNAs were first discovered in eukaryotic cells and were found in almost all organisms. They are abundant and evolutionarily conservative in eukaryotic cells (Hsu and Coca-Prados, 1979). Since then, thousands of circRNAs have been found in animals ranging from *Drosophila melanogaster* to *Homo sapiens* (Huang et al., 2017). CircRNAs are mostly produced from exons and have a wide variety of species, such as exon circRNAs, exon-intron circRNAs, intron circRNAs, antisense circRNAs, intergenic circRNAs, and sensory-overlap circRNAs (Guarnerio et al., 2019). The production of circRNAs mainly depends on two mechanisms. RBPs bind to introns with long inverted repeats at two ends of linear RNA and promote the binding of the two ends of linear RNA together to allow circRNAs formation. Some RBPs have been found to promote the formation of some circRNAs including the splicing factor muscleblind (Ashwal-Fluss et al., 2014), Quaking (Conn et al., 2015), RNA-binding motif protein 20 (Khan et al., 2016), and the RBP FUS (Errichelli et al., 2017), Muscleblind (Ashwal-Fluss et al., 2014), and so on. Furthermore, the RNA pairing of the complementary sequences at two ends of linear RNAs leads to circRNA formation (Patop et al., 2019).

Functionally, circRNAs play an important role in regulating gene expression in various ways, such as modulating transcription, alternative splicing, RNA processing reactions, being translated into polypeptides, interacting with RBPs, and sequestrating of miRNAs or proteins (Kristensen et al., 2019). Several studies have revealed that circRNAs are involved in the physiological and pathological processes, such as OP (Shen et al., 2020), osteosarcoma (Liu et al., 2017), Alzheimer disease, diabetes mellitus, malignant tumors (Li et al., 2018), and osteoarthritis (Ouyang et al., 2017; Shen et al., 2019). Also, circRNAs are implicated in neuronal function, innate immune responses, cell proliferation, and pluripotency (Li et al., 2019; Shi et al., 2020; Li and Chen, 2021). CircRNAs participate in the osteogenic differentiation of several kinds of MSCs including BMSCs by sponging miRNAs (Gu et al., 2017; Ouyang et al., 2019; Peng et al., 2019).

### 4.2 CircRNAs That Promote Osteogenic Differentiation in BMSCs

**FIGURE 6:**
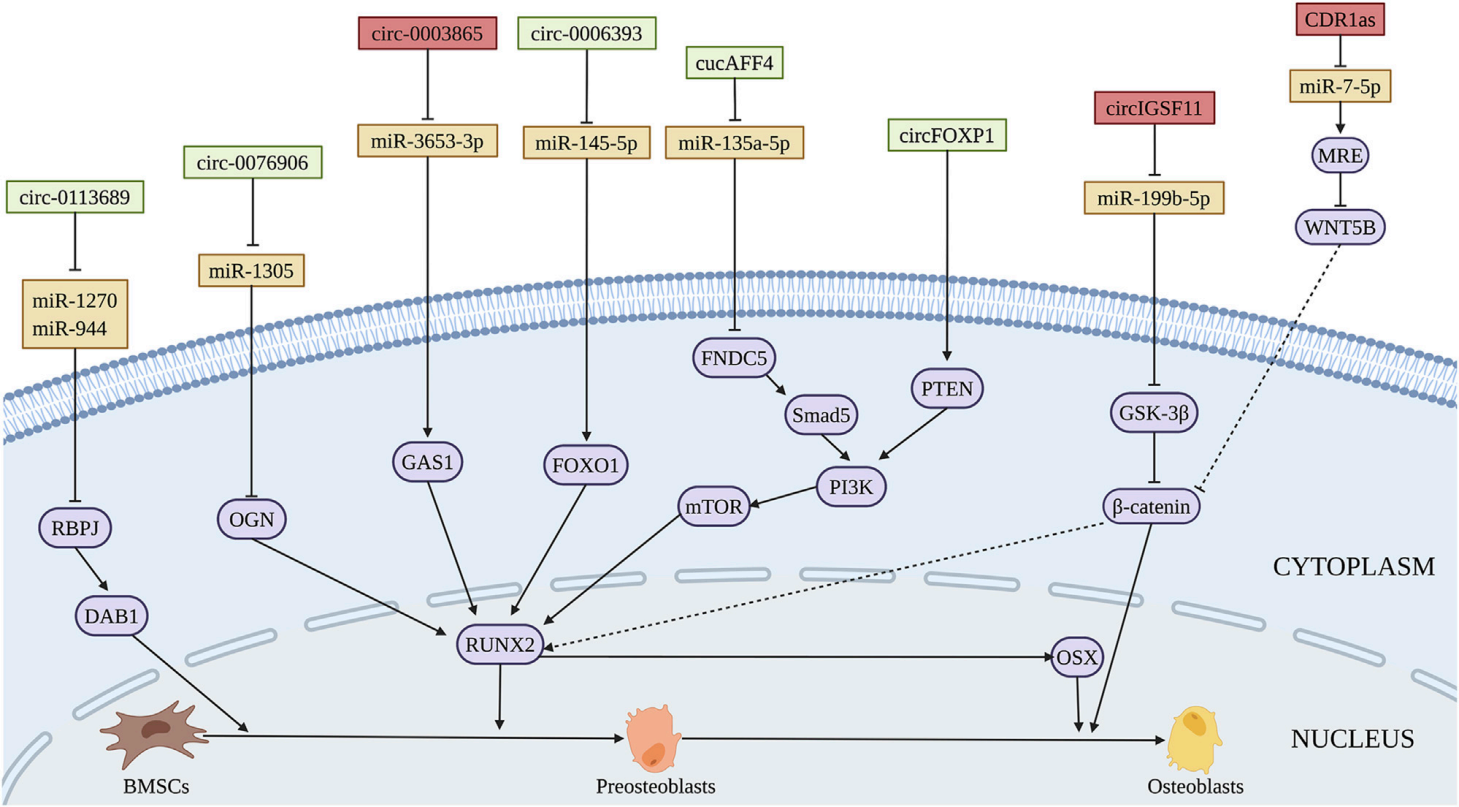
Illustration of the role and regulatory mechanism of circRNAs-promoted (green) and inhibited (red) osteogenic differentiation of BMSCs. Some circRNAs inhibit BMSCs osteogenesis through sponging pro-osteogenic miRNAs (orange). (Created with BioRender.com).

Circ_0113689 originated from gene DAB1 binds miR-1270 and miR-944 to enhance the osteogenic differentiation of BMSCs, finally exerted promoting role in chondrogenesis through NOTCH/RBPJ pathway (Chia et al., 2020) (Figure 6 and Table 5). During NOTCH/RBPJ signaling pathway, the Notch intracellular domain translocates to the nucleus and binds with RBPJ and co-activators, forming a complex that induces the transcription of downstream gene DAB1 (Luo et al., 2019). CircRNA AFF4 activates the expression of fibronectin type III domain-containing protein 5 (FNDC5)/Irisin through Smad1/5 pathway via sponging miR-135a-5 p, which induces the osteogenic differentiation of BMSCs *in vitro* and ectopic bone formation *in vivo* (Liu et al., 2021). Moreover, circ_AFF4 was reported to promote osteoblastic proliferation by acting as a miR-7223-5p sponge (Mi et al., 2019). During the bone-related diseases progression, the expressions of circ_0076906 is greatly decreased both in the bone tissue and serum of OP patients. Circ_0076906 promotes osteogenic differentiation of BMSCs through regulating to miR-1305 and its target osteoglycin (OGN), finally alleviates the OP progression (Wen et al., 2020). Circ_0006393 was decreased in the bone tissue of patients with glucocorticoid-induced OP. Further study demonstrated that circ_0006393 overexpression increases bone metabolism through miR-145-5p-FOXO1 pathway (Wang et al., 2019). CircFOXP1 regulates PTEN gene expression, thereby promoting the osteogenic differentiation of BMSCs through PI3K/AKT pathway, which may be used as the therapeutic targets in bone-related diseases such as ONFH (Xin et al., 2021). In addition, The expressions of circ_0000219 and circ_0005936 are significantly decreased in the bone marrow tissue of ONFH patients, which may be related to the proliferation and osteogenic capacity of BMSCs from ONFH patients (Xiang et al., 2020).

**TABLE 5:** CircRNAs that regulate the osteogenic differentiation in BMSCs and underlying mechanisms.

CircRNAs	Study model	Signaling pathway	Effect	References
circ-DAB1 (has_circ_0113689)	BMSCs *in vitro*	Binds miR-1270 and miR-944 to activate NOTCH/RBPJ pathway	Promotes expression of ALP, RUNX2, OSX, OCN, and COL1A1	Chia et al. (2020)
circ_0,076906	BMSCs *in vitro*	Targets miR-942–5p to increase the expression of RUNX2 and VEGF via the miR-1305/OGN pathway	Promotes expression of RUNX2 and OCN	Wen et al. (2020)
hsa_circ_0066523 (circFOXP1)	BMSCs *in vitro*	Promotes PTEN gene expression via the PI3K/AKT pathway	Promotes expression of RUNX2, OPN, and OCN	Xin et al. (2021)
circ_0000219, circ_0005936	BMSCs from healthy donors and patients with SONFH	——	Changes cellular functions and aberrantly expressed miRNAs and circRNAs in bone marrow stem cells in ONFH	Shuai Xiang et al. (2020)
circRNA CDR1as	BMSCs from patients with steroid-induced ONFH and femoral head fracture *in vitro*	Sponges miR-7-5p to promote CDR1as and WNT5B expression via the Wnt/β-catenin signaling	Inhibits expression of RUNX2, OSX, BMP2, ALP, and OCN	Gaoyang Chen et al. (2020)
CircIGSF11	BMSCs *in vitro*	Targets miR-199b-5p to promote osteogenesis *via* the GSK-3β/β-catenin signaling pathway	Inhibits expression of ALP, RUNX2, OCN, and OSX	Ouyang et al. (2019)
hsa-circ-0000885	BMSCs from OP patients	——	The expression of hsa-circ-0000885 was upregulated in peripheral blood mononuclear cells of OP patients	Zhao et al. (2021)
Circ_0,006,393	BMSCs from healthy donors and glucocorticoid-induced OP patients	increases bone metabolism through miR-145-5p-FOXO1 pathway	Promotes expression of RUNX2, OPG, BMP2, and OSX transcription factor	Xing-Bo Wang et al. (2019)
circ_0,003,865	BMSCs *in vitro*	circ_0,003,865 sponges miR-3653–3p to regulate GAS1 gene expression through NF-κB pathways	Inhibits expression of RUNX2, ALP, and OPN	Wang et al. (2021)
Circular RNA AFF4	BMSCs *in vitro*, facture model in nude mice	activates the expression of FNDC5/Irisin through Smad1/5 pathway *via* sponging miR-135a-5p	Promotes expression of ALP, BMP4, RUNX2 at both mRNA and protein levels	Chao Liu et al. (2021)

### 4.3 CircRNAs That Inhibit Osteogenic Differentiation in BMSCs

CircIGSF11 is downregulated during osteogenic differentiation of BMSCs. Silencing of circIGSF11 may promote osteogenesis through regulating miR-199b-5p of BMSCs (Ouyang et al., 2019). Chen et al. (2020) determined that circRNA CDR1as suppresses the expression of CDR1as and WNT5B via sponging miR-7-5p, which inhibits the osteogenic differentiation of BMSCs from patients with SONFH. Furthermore, circ_0003865 sponges miR-3653-3p to regulate growth arrest-specific gene 1 (GAS1) gene expression through NF-κB pathways, thereby inhibiting the osteogenic differentiation of BMSCs in the bone marrow tissue of OP patients (Wang et al., 2021). The expression of hsa-circ-0000885 was upregulated in peripheral blood mononuclear cells of OP patients. Circ-0000885 silencing has the potential to promote cell proliferation, osteogenic differentiation, and inhibit apoptosis of BMSCs (Zhao et al., 2021)**.** The regulatory function and mechanism of circRNAs on osteogenic differentiation of BMSCs are summarized in Figure 6 and Table 5.

## 5 PiRNAs and Osteogenic Differentiation of BMSCs

In addition, piRNAs also participate in the osteogenic differentiation of BMSCs. PiRNAs are a kind of linear ncRNAs with a length of 26–31 nucleotides, which are to perform their biological functions by binding with PIWI protein (PIWIL) proteins (Iwasaki et al., 2015). The piRNA biogenesis pathways are complex and conserved, including *de novo* piRNA production, the ping-pong cycle, and self-amplification mechanisms, resulting in mature piRNAs (Zhang et al., 2022). Some studies have suggested that piRNA plays an essential role in maintaining the functionality of stem cells, formatting, and differentiating germ cells and somatic cells (Vagin et al., 2006; Lin et al., 2020; Li et al., 2021). In the *Drosophila* germline, the binding of piRNA and repeat-associated small interfering RNA ensure genomic stability by silencing transposable elements and participate in the whole process of spermatogonial generation, development, and differentiation (Vagin et al., 2006). The change of chromatin state during cell differentiation creates a circumstance in which specific transposons can be expressed, the binding of piRNA and PIWIL SMEDWI-2 participates in the regulation of somatic differentiation by specifically silencing these transposons in different cell types (Li et al., 2021). Reports demonstrated that piRNAs are involved in the osteogenic differentiation of BMSCs. RNA sequencing confirmed that 8 piRNAs are upregulated and 46 piRNAs are downregulated in the early osteogenic differentiation of BMSCs, but it is not clear whether these piRNAs are involved in the osteogenic differentiation of BMSCs (Della Bella et al., 2020). According to these dysregulated piRNAs, Liu et al. further confirmed that the binding of piR-36741 and PIWIL4 protein suppresses methyltransferase like 3-mediated BMP2 m^6^A level and promotes BMP2 expression, thereby increasing the osteogenic differentiation in BMSCs (Liu et al., 2021). The functional regulation and mechanism of piRNAs on osteogenic differentiation in BMSCs should be revealed. And the potential in bone regeneration of piRNAs and whether piRNAs are involved in bone-related diseases should be further evaluated.

## 6 Exsosomal ncRNAs and Osteogenesis

Exosomes are a kind of extracellular vesicles with a diameter of 40–100 nm (Raposo and Stoorvogel, 2013). Exosomes exist in human body fluids such as saliva, blood, and breast milk (Admyre et al., 2007; Michael et al., 2010), and can be secreted by various cells including MSCs (Raposo and Stahl, 2019). Exosomes are encapsulated by lipid bilayers, which could protect their contents from degradation. According to the different source cells, the components of exosome contents include miRNAs, lncRNAs, proteins, lipids, amino acids, etc., (Kalluri and LeBleu, 2020). Exosomes deliver these small molecules to recipient cells, thus participating in bone regeneration and other processes (Huang et al., 2020).

**FIGURE 7:**
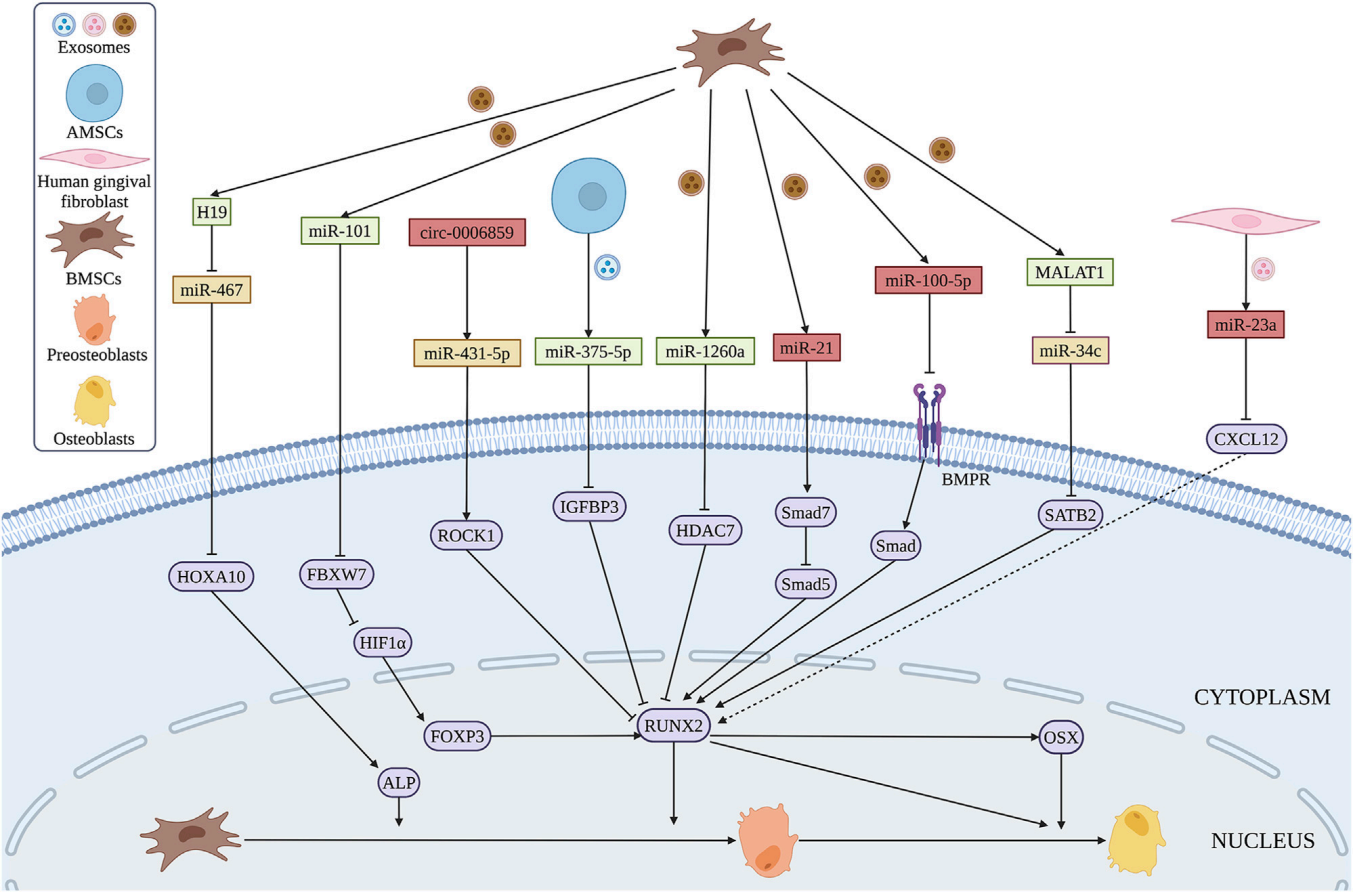
Exosomal ncRNAs derived from BMSCs (brown), AMSCs (blue) and human gingival fibroblasts (pink) can promote (green) or inhibit (red) osteogenic differentiation of BMSCs *via* various regulatory mechanisms. (Created with BioRender.com).

The expression of miR-199b, miR-218a, miR-148a, miR-135b, miR-203, miR-219, miR-299-5p, and miR-302b were significantly increased during the osteogenic differentiation of BMSCs, while the expression of miR-221, miR-155, miR-885-5p, miR-181a and miR-320c is decreased (Xu et al., 2014). Li et al. (2021) found that exosomal miR-101 derived from BMSCs promotes the osteogenic differentiation of BMSCs by targeting F-box and WD repeat domain containing 7 (FBXW7), as well as modulating FBXW7-mediated hypoxia-inducible factor-1α (HIF1α)/FOXP3 axis (Figure 7). In addition, overexpression of exosomal miR-375-5p derived from human adipose mesenchymal stem cells (AMSCs) promotes the osteogenic differentiation of BMSCs by targeting insulin-like growth factor binding protein 3 (IGFBP3) (Chen et al., 2019). Some exosomal miRNAs inhibit the osteogenic differentiation of BMSCs. Jiang et al. (2018) found an increased level of miR-21 in exosomes extracted from BMSCs in OP patients that inhibits the osteogenic differentiation of BMSCs by targeting Smad7. Furthermore, exosomal miR-23a secreted by human gingival fibroblasts inhibits the osteogenic capacity of BMSCs by targeting CXCL12 (Zhuang and Zhou, 2020). Exosomal miR-100-5p inhibits the osteogenic differentiation of BMSCs by targeting BMPR2 through BMPR2/Smad1/5/9 signaling pathway (Yang et al., 2021). Intriguingly, exosomal miR-1260a, which is also derived from BMSCs treated with Fe_3_O_4_ and a static magnetic field, promotes osteogenic differentiation of BMSCs by targeting, which provides the potential for bone regeneration of tissue engineering (Wu et al., 2021).

Similar to miRNAs, several lncRNAs from exosomes of BMSCs exert great functions in osteogenic differentiation. SATB2 was proved to promote osteogenic differentiation of BMSCs in patients with osteonecrosis (Yang et al., 2019). Exosomes containing MALAT1 promote osteogenic differentiation of BMSCs through the interaction between MALAT1 and miR-34c/SATB2 axis *in vitro* (Yang et al., 2019). Moreover, exosomal H19 derived from BMSCs reverses poor the osteogenic differentiation of BMSCs during obesity-induced fracture healing *via* miR-467/HOXA10 axis (Zhi et al., 2021).

CircRNAs are abundant in exosomes, and more than one thousand exosomal circRNAs have been identified in human serum (Fanale et al., 2018). The expression of circ_0006859 in exosomes is upregulated in OP patients. Exosomal circ_0006859 suppresses osteogenesis of BMSCs by sponging miR-431-5p and then elevated Rho-associated coiled-coil containing protein kinase 1 (ROCK1) expression (Zhi et al., 2021).

TsRNAs, which are classified into tRNA-derived stress-induced RNAs and tRNA-derived fragments are small fragments of RNAs generated from tRNAs by specific ribonucleases, such as dicer and angiogenin (Zong et al., 2021). Several reports showed that tsRNAs are involved in the physical and diseases processes (Kim et al., 2017; Zhu et al., 2019). Furthermore, tsRNAs are found to be dysregulated in the exomes during osteogenic differentiation of BMSCs. Yan et al. demonstrated that several tsRNAs including Ser-ACT, Ser-GCT, Sup-TTA, Phe-GAA, Ile-AAT, Lys-TTT, Leu-TAG, and Thr-CGT are significantly upregulated, while the expression of Gly-CCC, Gly-GCC, and His-GTG is downregulated in the exosomes during the osteogenic differentiation in BMSCs. However, the specific function of these tsRNAs should be further revealed. The regulatory function and mechanism, and potential application in bone regeneration of more ncRNAs should be further explored. Exosomal ncRNAs in the regulating of osteogenic differential in BMSCs are shown in Figure 7.

## 7 Challenges and Perspectives

BMSCs are an important source of stem cells in bone tissue engineering, which have a good application prospect in the fields of bone tissue engineering and bone regeneration. BMSCs also have great potential in the therapy of bone-related diseases such as OP and OFNH (Qi et al., 2017). The osteogenic differentiation of BMSCs is a complex physiological process. There is increasing evidence that this process is regulated by different epigenetic factors, including ncRNAs. This review focused on the function and regulatory mechanism of ncRNAs in the osteogenic differentiation of BMSCs. It has made great progress on the osteogenic differentiation of BMSCs regulated by ncRNAs. However, there are still much more to know about the functions and mechanisms of ncRNAs in regulating the osteogenic differentiation of BMSCs.

Firstly, more ncRNAs should be explored by sequencing. As we reviewed above, exosomes derived ncRNAs are not fully elucidated. Currently, only a few piRNAs and tsRNAs are identified during the osteogenic differentiation of BMSCs. And the function and regulation of these new piRNAs and tsRNAs are not clear. To our knowledge, no reports show that other ncRNAs such as rRNAs, snRNAs, snoRNAs are involved in influencing the osteogenic capability of BMSCs, which needs much more attention. Mostly, the regulatory mechanism of lncRNAs and circRNAs are involved in sponging miRNAs during the osteogenic processes of BMSCs. PiRNAs may also have the binding potential to lncRNAs and circRNAs. Thus, the regulatory network needs much deeper mining through bioinformatic analysis. Furthermore, the epigenetic regulation of lncRNAs such as methylation, and binding of transcription factors during the osteogenesis of BMSCs should be paid more attention.

Secondly, lncRNA and circRNA-encoded small peptides were identified by the computational and analytical methods used to forecast prospective ncRNAs encoding oligopeptides (Wu et al., 2020). These peptides have specific biological functions such as tumor development and inflammatory responses (Huang. et al., 2017; Niu et al., 2020; Wu et al., 2020; Gao et al., 2021). However, few reports show that lncRNA and circRNA-encoded peptides participate in the osteogenic differentiation of MSCs.

Finally, more efforts should be made to increase the clinical application for bone regeneration and bone-related diseases in the future. These ncRNAs influenced the osteogenic differentiation of BMSCs has the potential as targets by overexpression or inhibition. The efficient delivery system into BMSCs is rather important. These ncRNAs could be incorporated into exosomes and then delivered into the BMSCs. These modified BMSCs alone or in combination with biomaterials can be directly injected into bone defect sites.

## 8 Conclusion

In summary, research on the function of ncRNAs in the regulation of osteogenic differentiation of BMSCs has made great processes. The ncRNAs could be biomarkers of bone-related diseases. MiRNAs, circRNAs, and lncRNAs are the most extensively investigated ncRNAs for their regulatory role in the osteogenic differentiation of BMSCs and the underlying mechanisms. The osteogenic differentiation regulation potentials of piRNAs, tsRNAs, rRNAs, snRNAs, and snoRNAs are still unclear. The current understanding of the regulatory role of different miRNAs, circRNAs, and lncRNAs in osteogenic differentiation of BMSCs could be applied for bone tissue regeneration, such uses of exosomes or nanoparticles carrying osteo-stimulatory ncRNAs. However, the clinical applications of ncRNAs in bone tissue engineering are hardly reported. Therefore, future preclinical studies are mandatory to evaluate the efficacy and safety of ncRNAs-mediated BMSCs-based bone tissue engineering.

## Glossary

AA: aplastic anemia
AIS: adolescent idiopathic scoliosis
AKT: protein kinase B
ALP: alkaline phosphatase
AMSC: adipose mesenchymal stem cells
ARL6: adenosine diphosphate-ribosylation factor-like protein 6
ATF: activating transcription factor
BCL2: B-cell lymphoma 2
BMP: bone morphogenetic protein
BMPR: BMP receptor
BMSC: bone marrow derived mesenchymal stem cell
CDH11: cadherin-11
ceRNA: competitive endogenous lncRNAs
circRNA: circular RNA
CNNB1: Targets catenin beta 1
COL1: collagen type I
COL1A1: collagen type I α1
CTNNBIP1: catenin beta interacting protein 1
CXCR4: C-X-C chemokine receptor type 4
DANCR: differentiation antagonizing non-protein coding RNA
DDX17: dead-box helicase 17
DEPTOR: DEP domain-containing mTOR interacting protein
DHRS3: retinoic acid-inducible dehydrogenase reductase 3
DKK1: dickkopf 1
DLX: distal-less
EFNA3: ephrin-A3
ERK: extracellular signal-regulated kinase
EZH2: enhancer of zeste homolog 2
FAK: focal adhesion kinase
FBXW7: F-box and WD repeat domain containing 7
FGF: fibroblast growth factor
FNDC5: fibronectin type III domain-containing protein 5
FOX: forkhead box
FZD: frizzled
GAS1: growth arrest-specific gene 1
GLS: glutaminase
GSK-3β: glycogen synthetase kinase 3 beta
H3K27: trimethylation of lysine 27 on histone H3
HA/TCP: hydroxyapatite/tricalcium phosphate
HIF1α: hypoxia-inducible factor-1α
HMGA2: high-mobility group A2
hnRNPK: heterogeneous nuclear ribonucleoprotein K
HOTAIR: HOX transcript antisense RNA
HOTTIP: HOX transcript at the distal tip
HOX: homeobox
IGF1R: insulin growth factor 1 receptor
IGFBP3: insulin-like growth factor binding protein 3
IL-1β: interleukin-1β
KLF: kruppel like factor
K-RAS: kirsten rat sarcoma viral oncogene
lncRNA: long non-coding RNA
lncRNA-OG: osteogenesis-associated lncRNA
LOXL1-AS1: LOXL1 antisense RNA 1
LRP5: low-density lipoprotein receptor-related protein 5
MAPK: mitogen-activated protein kinase
MEF2C: myocyte enhancer factor 2 C
MEG3: maternally expressed 3 (nonprotein coding)
miRNA: microRNA
MRC2: type 2 mannose receptor C
MSC: mesenchymal stem cell
mTOR: mammalian target of rapamycin
MYD88: myeloid differentiation factor 88
ncRNA: non-coding RNA
NF-κB: nuclear factor kappa B
NOTCH1: notch recepetor 1
OCN: osteocalcin
OGN: osteoglycin
ONFH: osteonecrosis of the femoral head
OP: osteoporosis
OPN: osteopontin
OSX: osterix
PI3K: phosphoinositide 3-kinase
piRNA: PIWI-interacting RNA
PIWIL: PIWI protein
PMOP: postmenopausal osteoporosis
PPARγ: peroxisome proliferator-activated receptor γ
pre-miRNA: precursor miRNA
pri-miRNA: primary miRNA
PROX1: prospero homeobox 1
PTEN: phosphatase and tensin homolog deleted on chromosome ten
PWRN1-209: Prader-willi region ncRNAs 1–-209
RANKL: receptor or activator of NF-κB ligand
RAP1A: ras-related protein 1 AA
RBP: RNA-binding protein
ROCK1: Rho associated coiled-coil containing protein kinase 1
rRNA: ribosomal RNA
RUNX2: runt-related transcription factor 2
SATB2: special AT-rich binding protein homeobox 2
SDF-1: stromal cell-derived factor 1
SEMA3A: semaphorin 3 AA
SIRT1: sirtuin1
SLC39A1: solute carrier family 39 member 1
SMURF2: Smad ubiquitin regulatory factor 2
SNHG16: small nucleolar RNA host gene 16
snoRNA: small nucleolar RNA
snRNA: small nuclear RNA
SONFH: steroid-associated osteonecrosis of the femoral head
SOX2: sex determining region Y-box 2
SPRY1: Sprouty 1
STAT1: signal transducer and activator of transcription 1
TCF-1: T-cell factor 1
TET2: ten-eleven translocation 2
TGF-β: transforming growth factor-β
THA: total hip arthroplasty
TNF-α: tumor necrosis factor-α
tRNA: transfert RNA
tsRNA: tRNA-derived small RNA
TUG1: taurine upregulated gene 1
VEGF: vascular endothelial growth factor
WDR5: WD Repeat-Containing Protein 5
WISP2: WNT1 inducible signaling pathway protein 2
WNT3A: Wnt family member 3 AA
WWP1: WW domain-containing E3 ubiquitin protein ligase 1
XIST: X inactivate-specific transcript

## References

[B1] Abdelfattah A. M. Park C. Choi M. Y. (2014). Update on Non-canonical microRNAs. Biomol. Concepts 5 (4), 275–287. 10.1515/bmc-2014-0012 25372759 PMC4343302

[B2] Admyre C. Johansson S. M. Qazi K. R. Filén J.-J. Lahesmaa R. Norman M. (2007). Exosomes with Immune Modulatory Features Are Present in Human Breast Milk. J. Immunol. 179 (3), 1969–1978. 10.4049/jimmunol.179.3.1969 17641064

[B3] Akkouch A. Eliason S. Sweat M. E. Romero-Bustillos M. Zhu M. Qian F. (2019). Enhancement of MicroRNA-200c on Osteogenic Differentiation and Bone Regeneration by Targeting Sox2-Mediated Wnt Signaling and Klf4. Hum. Gene Ther. 30 (11), 1405–1418. 10.1089/hum.2019.019 31288577 PMC6854517

[B4] Arthur A. Gronthos S. (2020). Clinical Application of Bone Marrow Mesenchymal Stem/Stromal Cells to Repair Skeletal Tissue. Ijms 21 (24), 9759. 10.3390/ijms21249759 PMC7767389 33371306

[B5] Ashwal-Fluss R. Meyer M. Pamudurti N. R. Ivanov A. Bartok O. Hanan M. (2014). circRNA Biogenesis Competes with Pre-mRNA Splicing. Mol. Cell. 56 (1), 55–66. 10.1016/j.molcel.2014.08.019 25242144

[B6] Asila A. Yang X. Kaisaer Y. Ma L. (2021). SNHG16/miR‐485‐5p/BMP7 axis Modulates Osteogenic Differentiation of Human Bone Marrow‐derived Mesenchymal Stem Cells. J. Gene Med. 23 (3), e3296. 10.1002/jgm.3296 33179372

[B7] Bahamonde M. E. Lyons K. M. (2001). BMP3: to Be or Not to Be a BMP. J. Bone Jt. Surgery-American Volume 83 (Pt 1), S1–S56. 10.2106/00004623-200100001-00008 11263666

[B8] Bi H. Wang D. Liu X. Wang G. Wu X. (2020). Long Non-coding RNA H19 Promotes Osteogenic Differentiation of Human Bone Marrow-Derived Mesenchymal Stem Cells by Regulating microRNA-140-5p/SATB2 axis. J. Biosci. 45, 56. 10.1007/s12038-020-0024-y 32345782

[B9] Bian W. Xiao S. Yang L. Chen J. Deng S. (2021). Quercetin Promotes Bone Marrow Mesenchymal Stem Cell Proliferation and Osteogenic Differentiation through the H19/miR-625-5p axis to Activate the Wnt/β-Catenin Pathway. BMC Complement. Med. Ther. 21 (1), 243. 10.1186/s12906-021-03418-8 34592982 PMC8485455

[B10] Bruderer M. Richards R. G. Alini M. Stoddart M. (2014). Role and Regulation of RUNX2 in Osteogenesis. eCM 28, 269–286. 10.22203/ecm.v028a19 25340806

[B11] Cai W. L. Zeng W. Liu H. H. Zhu B. Y. Liu J. L. Liu Y. (2020). LncRNA LINC00707 Promotes Osteogenic Differentiation of hBMSCs through the Wnt/β-Catenin Pathway Activated by LINC00707/miR-145/LRP5 axis. Eur. Rev. Med. Pharmacol. Sci. 24 (1), 18–28. 10.26355/eurrev_202001_19891 31957814

[B12] Camp E. Pribadi C. Anderson P. J. Zannettino A. C. W. Gronthos S. (2018). miRNA-376c-3p Mediates TWIST-1 Inhibition of Bone Marrow-Derived Stromal Cell Osteogenesis and Can Reduce Aberrant Bone Formation of TWIST-1 Haploinsufficient Calvarial Cells. Stem Cells Dev. 27 (23), 1621–1633. 10.1089/scd.2018.0083 30229694

[B13] Cao Y. Lv Q. Lv C. (2015). MicroRNA-153 Suppresses the Osteogenic Differentiation of Human Mesenchymal Stem Cells by Targeting Bone Morphogenetic Protein Receptor Type II. Int. J. Mol. Med. 36 (3), 760–766. 10.3892/ijmm.2015.2275 26151470

[B14] Chan W. C. W. Tan Z. To M. K. T. Chan D. (2021). Regulation and Role of Transcription Factors in Osteogenesis. Ijms 22 (11), 5445. 10.3390/ijms22115445 34064134 PMC8196788

[B15] Chau D. L. Edelman S. V. Chandran M. (2003). Osteoporosis and Diabetes. Curr. Diab Rep. 3 (1), 37–42. 10.1007/s11892-003-0051-8 12643144

[B16] Che M. Gong W. Zhao Y. Liu M. (2020). Long Noncoding RNA HCG18 Inhibits the Differentiation of Human Bone Marrow-Derived Mesenchymal Stem Cells in Osteoporosis by Targeting miR-30a-5p/NOTCH1 axis. Mol. Med. 26 (1), 106. 10.1186/s10020-020-00219-6 33176682 PMC7656763

[B17] Chen G. Wang Q. Li Z. Yang Q. Liu Y. Du Z. (2020). Circular RNA CDR1as Promotes Adipogenic and Suppresses Osteogenic Differentiation of BMSCs in Steroid-Induced Osteonecrosis of the Femoral Head. Bone 133, 115258. 10.1016/j.bone.2020.115258 32018039

[B18] Chen L. Luo W. Wang Y. Song X. Li S. Wu J. (2021). Directional Homing of Glycosylation-Modified Bone Marrow Mesenchymal Stem Cells for Bone Defect Repair. J. Nanobiotechnol 19 (1), 228. 10.1186/s12951-021-00969-3 PMC8325817 34332597

[B19] Chen Q. Zhang X. Shi J. Yan M. Zhou T. (2021). Origins and Evolving Functionalities of tRNA-Derived Small RNAs. Trends Biochem. Sci. 46 (10), 790–804. 10.1016/j.tibs.2021.05.001 34053843 PMC8448906

[B20] Chen S. Jia L. Zhang S. Zheng Y. Zhou Y. (2018). DEPTOR Regulates Osteogenic Differentiation via Inhibiting MEG3-Mediated Activation of BMP4 Signaling and Is Involved in Osteoporosis. Stem Cell. Res. Ther. 9 (1), 185. 10.1186/s13287-018-0935-9 29973283 PMC6033203

[B21] Chen S. Tang Y. Liu Y. Zhang P. Lv L. Zhang X. (2019). Exosomes Derived from miR‐375‐overexpressing Human Adipose Mesenchymal Stem Cells Promote Bone Regeneration. Cell. Prolif. 52 (5), e12669. 10.1111/cpr.12669 31380594 PMC6797519

[B22] Chen S. Yang L. Jie Q. Lin Y.-S. Meng G.-L. Fan J.-Z. (2014). MicroRNA-125b Suppresses the Proliferation and Osteogenic Differentiation of Human Bone Marrow-Derived Mesenchymal Stem Cells. Mol. Med. Rep. 9 (5), 1820–1826. 10.3892/mmr.2014.2024 24604278

[B23] Chen Y. Yang Y.-R. Fan X.-L. Lin P. Yang H. Chen X.-Z. (2019). miR-206 Inhibits Osteogenic Differentiation of Bone Marrow Mesenchymal Stem Cells by Targetting Glutaminase. Biosci. Rep. 39 (3), BSR20181108. 10.1042/bsr20181108 30804229 PMC6900431

[B24] Chen Y. Yu H. Zhu D. Liu P. Yin J. Liu D. (2020). miR‐136‐3p Targets PTEN to Regulate Vascularization and Bone Formation and Ameliorates Alcohol‐induced Osteopenia. FASEB J. 34 (4), 5348–5362. 10.1096/fj.201902463RR 32072664

[B25] Cheng F. Yang M. M. Yang R. H. (2019). MiRNA-365a-3p Promotes the Progression of Osteoporosis by Inhibiting Osteogenic Differentiation via Targeting RUNX2. Eur. Rev. Med. Pharmacol. Sci. 23 (18), 7766–7774. 10.26355/eurrev_201909_18986 31599402

[B26] Chia W. Liu J. Huang Y.-G. Zhang C. (2020). A Circular RNA Derived from DAB1 Promotes Cell Proliferation and Osteogenic Differentiation of BMSCs via RBPJ/DAB1 axis. Cell. Death Dis. 11 (5), 372. 10.1038/s41419-020-2572-3 32415085 PMC7229165

[B27] Ciapetti G. Granchi D. Fotia C. Savarino L. Dallari D. Del Piccolo N. (2016). Effects of Hypoxia on Osteogenic Differentiation of Mesenchymal Stromal Cells Used as a Cell Therapy for Avascular Necrosis of the Femoral Head. Cytotherapy 18 (9), 1087–1099. 10.1016/j.jcyt.2016.06.005 27421741

[B28] Conn S. J. Pillman K. A. Toubia J. Conn V. M. Salmanidis M. Phillips C. A. (2015). The RNA Binding Protein Quaking Regulates Formation of circRNAs. Cell. 160 (6), 1125–1134. 10.1016/j.cell.2015.02.014 25768908

[B29] Dai Z. Jin Y. Zheng J. Liu K. Zhao J. Zhang S. (2019). MiR-217 Promotes Cell Proliferation and Osteogenic Differentiation of BMSCs by Targeting DKK1 in Steroid-Associated Osteonecrosis. Biomed. Pharmacother. 109, 1112–1119. 10.1016/j.biopha.2018.10.166 30551361

[B30] Dawlaty M. M. Breiling A. Le T. Barrasa M. I. Raddatz G. Gao Q. (2014). Loss of Tet Enzymes Compromises Proper Differentiation of Embryonic Stem Cells. Dev. Cell. 29 (1), 102–111. 10.1016/j.devcel.2014.03.003 24735881 PMC4035811

[B31] Della Bella E. Menzel U. Basoli V. Tourbier C. Alini M. Stoddart M. J. (2020). Differential Regulation of circRNA, miRNA, and piRNA during Early Osteogenic and Chondrogenic Differentiation of Human Mesenchymal Stromal Cells. Cells 9 (2), 398. 10.3390/cells9020398 PMC7072123 32050423

[B32] Delloye C. van Cauter M. Dufrane D. Francq B. G. Docquier P. L. Cornu O. (2014). Local Complications of Massive Bone Allografts: an Appraisal of Their Prevalence in 128 Patients. Acta Orthop. Belg 80 (2), 196–204. 25090792

[B33] Deng L. Hu G. Jin L. Wang C. Niu H. (2018). Involvement of microRNA-23b in TNF-α-Reduced BMSC Osteogenic Differentiation via Targeting Runx2. J. Bone Min. Metab. 36 (6), 648–660. 10.1007/s00774-017-0886-8 29234953

[B34] Ding Y. Wang L. Zhao Q. Wu Z. Kong L. (2019). MicroRNA-93 I-nhibits C-hondrocyte A-poptosis and I-nflammation in O-steoarthritis by T-argeting the TLR4/NF-κB S-ignaling P-athway. Int. J. Mol. Med. 43 (2), 779–790. 10.3892/ijmm.2018.4033 30569118 PMC6317687

[B35] Divisato G. Piscitelli S. Elia M. Cascone E. Parisi S. (2021). MicroRNAs and Stem-like Properties: The Complex Regulation Underlying Stemness Maintenance and Cancer Development. Biomolecules 11 (8), 1074. 10.3390/biom11081074 34439740 PMC8393604

[B36] Du K. Li Z. Fang X. Cao T. Xu Y. (2017). Ferulic Acid Promotes Osteogenesis of Bone Marrow-Derived Mesenchymal Stem Cells by Inhibiting microRNA-340 to Induce β-catenin Expression through Hypoxia. Eur. J. Cell. Biol. 96 (6), 496–503. 10.1016/j.ejcb.2017.07.002 28764862

[B37] Duan L. Zhao H. Xiong Y. Tang X. Yang Y. Hu Z. (2018). miR-16-2* Interferes with WNT5A to Regulate Osteogenesis of Mesenchymal Stem Cells. Cell. Physiol. Biochem. 51 (3), 1087–1102. 10.1159/000495489 30476907

[B38] Ebrahim A. S. Sabbagh H. Liddane A. Raufi A. Kandouz M. Al-Katib A. (2016). Hematologic Malignancies: Newer Strategies to Counter the BCL-2 Protein. J. Cancer Res. Clin. Oncol. 142 (9), 2013–2022. 10.1007/s00432-016-2144-1 27043233 PMC11819263

[B39] Eisa N. H. Sudharsan P. T. Herrero S. M. Herberg S. A. Volkman B. F. Aguilar-Pérez A. (2021). Age-associated Changes in microRNAs Affect the Differentiation Potential of Human Mesenchymal Stem Cells: Novel Role of miR-29b-1-5p Expression. Bone 153, 116154. 10.1016/j.bone.2021.116154 34403754 PMC8935397

[B40] El-Rashidy A. A. Roether J. A. Harhaus L. Kneser U. Boccaccini A. R. (2017). Regenerating Bone with Bioactive Glass Scaffolds: A Review of *In Vivo* Studies in Bone Defect Models. Acta Biomater. 62, 1–28. 10.1016/j.actbio.2017.08.030 28844964

[B41] Eppley B. L. Pietrzak W. S. Blanton M. W. (2005). Allograft and Alloplastic Bone Substitutes: a Review of Science and Technology for the Craniomaxillofacial Surgeon. J. Craniofac Surg. 16 (6), 981–989. 10.1097/01.scs.0000179662.38172.dd 16327544

[B42] Errichelli L. Dini Modigliani S. Laneve P. Colantoni A. Legnini I. Capauto D. (2017). FUS Affects Circular RNA Expression in Murine Embryonic Stem Cell-Derived Motor Neurons. Nat. Commun. 8, 14741. 10.1038/ncomms14741 28358055 PMC5379105

[B43] Eskildsen T. Taipaleenmäki H. Stenvang J. Abdallah B. M. Ditzel N. Nossent A. Y. (2011). MicroRNA-138 Regulates Osteogenic Differentiation of Human Stromal (Mesenchymal) Stem Cells *In Vivo* . Proc. Natl. Acad. Sci. U.S.A. 108 (15), 6139–6144. 10.1073/pnas.1016758108 21444814 PMC3076836

[B44] Fan F. Y. Deng R. Qiu L. Wen Q. Zeng Y. Gao L. (2019a). miR-203a-3p.1 is Involved in the Regulation of Osteogenic Differentiation by Directly Targeting Smad9 in MM-MSCs. Oncol. Lett. 18 (6), 6339–6346. 10.3892/ol.2019.10994 31788111 PMC6865574

[B45] Fan F. Y. Deng R. Lai S. H. Wen Q. Zeng Y. Gao L. (2019b). Inhibition of MicroRNA-221-5p Induces Osteogenic Differentiation by Directly Targeting Smad3 in Myeloma Bone Disease Mesenchymal Stem Cell. Oncol. Lett. 18 (6), 6536–6544. 10.3892/ol.2019.10992 31788114 PMC6865756

[B46] Fanale D. Taverna S. Russo A. Bazan V. (2018). Circular RNA in Exosomes. Adv. Exp. Med. Biol. 1087, 109–117. 10.1007/978-981-13-1426-1_9 30259361

[B47] Feng J. Wang J. X. Li C. H. (2019). LncRNA GAS5 Overexpression Alleviates the Development of Osteoporosis through Promoting Osteogenic Differentiation of MSCs via Targeting microRNA-498 to Regulate RUNX2. Eur. Rev. Med. Pharmacol. Sci. 23 (18), 7757–7765. 10.26355/eurrev_201909_18985 31599401

[B48] Feng L. Zhang J.-f. Shi L. Yang Z.-m. Wu T.-y. Wang H.-x. (2020). MicroRNA-378 Suppressed Osteogenesis of MSCs and Impaired Bone Formation via Inactivating Wnt/β-Catenin Signaling. Mol. Ther. - Nucleic Acids 21, 1017–1028. 10.1016/j.omtn.2020.07.018 32829178 PMC7452050

[B49] Fu L. Liu H. Lei W. (2020). MiR-596 Inhibits Osteoblastic Differentiation and Cell Proliferation by Targeting Smad3 in Steroid-Induced Osteonecrosis of Femoral Head. J. Orthop. Surg. Res. 15 (1), 173. 10.1186/s13018-020-01688-5 32410637 PMC7224111

[B50] Fu Y. C. Zhao S. R. Zhu B. H. Guo S. S. Wang X. X. (2019). MiRNA-27a-3p Promotes Osteogenic Differentiation of Human Mesenchymal Stem Cells through Targeting ATF3. Eur. Rev. Med. Pharmacol. Sci. 23 (3 Suppl. l), 73–80. 10.26355/eurrev_201908_18632 31389577

[B51] Fuller-Pace F. V. Ali S. (2008). The DEAD Box RNA Helicases P68 (Ddx5) and P72 (Ddx17): Novel Transcriptional Co-regulators. Biochem. Soc. Trans. 36 (Pt 4), 609–612. 10.1042/bst0360609 18631126

[B52] Gaihre B. Uswatta S. Jayasuriya A. (2017). Reconstruction of Craniomaxillofacial Bone Defects Using Tissue-Engineering Strategies with Injectable and Non-injectable Scaffolds. Jfb 8 (4), 49. 10.3390/jfb8040049 PMC5748556 29156629

[B53] Gao Y. Xiao F. Wang C. Wang C. Cui P. Zhang X. (2018). Long Noncoding RNA MALAT1 Promotes Osterix Expression to Regulate Osteogenic Differentiation by Targeting miRNA‐143 in Human Bone Marrow‐derived Mesenchymal Stem Cells. J. Cell. Biochem. 119 (8), 6986–6996. 10.1002/jcb.26907 29741283

[B54] Ge C. Xiao G. Jiang D. Franceschi R. T. (2007). Critical Role of the Extracellular Signal-Regulated Kinase-MAPK Pathway in Osteoblast Differentiation and Skeletal Development. J. Cell. Biol. 176 (5), 709–718. 10.1083/jcb.200610046 17325210 PMC2064027

[B55] Glinka A. Wu W. Delius H. Monaghan A. P. Blumenstock C. Niehrs C. (1998). Dickkopf-1 Is a Member of a New Family of Secreted Proteins and Functions in Head Induction. Nature 391 (6665), 357–362. 10.1038/34848 9450748

[B56] Goff L. A. Rinn J. L. (2015). Linking RNA Biology to lncRNAs. Genome Res. 25 (10), 1456–1465. 10.1101/gr.191122.115 26430155 PMC4579330

[B57] Gu X. Li M. Jin Y. Liu D. Wei F. (2017). Identification and Integrated Analysis of Differentially Expressed lncRNAs and circRNAs Reveal the Potential ceRNA Networks during PDLSC Osteogenic Differentiation. BMC Genet. 18 (1), 100. 10.1186/s12863-017-0569-4 29197342 PMC5712120

[B58] Guarnerio J. Zhang Y. Cheloni G. Panella R. Mae Katon J. Simpson M. (2019). Intragenic Antagonistic Roles of Protein and circRNA in Tumorigenesis. Cell. Res. 29 (8), 628–640. 10.1038/s41422-019-0192-1 31209250 PMC6796857

[B59] Guiducci G. Stojic L. (2021). Long Noncoding RNAs at the Crossroads of Cell Cycle and Genome Integrity. Trends Genet. 37 (6), 528–546. 10.1016/j.tig.2021.01.006 33685661

[B60] Guttman M. Russell P. Ingolia N. T. Weissman J. S. Lander E. S. (2013). Ribosome Profiling Provides Evidence that Large Noncoding RNAs Do Not Encode Proteins. Cell. 154 (1), 240–251. 10.1016/j.cell.2013.06.009 23810193 PMC3756563

[B61] Hao C. Yang S. Xu W. Shen J. K. Ye S. Liu X. (2016). MiR-708 Promotes Steroid-Induced Osteonecrosis of Femoral Head, Suppresses Osteogenic Differentiation by Targeting SMAD3. Sci. Rep. 6, 22599. 10.1038/srep22599 26932538 PMC4773864

[B62] He C. Liu M. Ding Q. Yang F. Xu T. (2021). Upregulated miR-9-5p Inhibits Osteogenic Differentiation of Bone Marrow Mesenchymal Stem Cells under High Glucose Treatment. J. Bone Min. Metab. 40 (2), 208–219. 10.1007/s00774-021-01280-9 34750680

[B63] Hong J. Guo F. Lu S.-Y. Shen C. Ma D. Zhang X. (2021). F. Nucleatum Targets lncRNA ENO1-IT1 to Promote Glycolysis and Oncogenesis in Colorectal Cancer. Gut 70 (11), 2123–2137. 10.1136/gutjnl-2020-322780 33318144

[B64] Hong L. Sharp T. Khorsand B. Fischer C. Eliason S. Salem A. (2016). MicroRNA-200c Represses IL-6, IL-8, and CCL-5 Expression and Enhances Osteogenic Differentiation. PLoS One 11 (8), e0160915. 10.1371/journal.pone.0160915 27529418 PMC4987006

[B65] Hsu M.-T. Coca-Prados M. (1979). Electron Microscopic Evidence for the Circular Form of RNA in the Cytoplasm of Eukaryotic Cells. Nature 280 (5720), 339–340. 10.1038/280339a0 460409

[B66] Hu M. Zhu X. Yuan H. Li H. Liao H. Chen S. (2021). The Function and Mechanism of the miR‐210‐3p/KRAS axis in Bone Marrow‐derived Mesenchymal Stem Cell from Patients with Osteoporosis. J. Tissue Eng. Regen. Med. 15 (8), 699–711. 10.1002/term.3215 33982450

[B67] Hu Z. Zhang L. Wang H. Wang Y. Tan Y. Dang L. (2020). Targeted Silencing of miRNA-132-3p Expression Rescues Disuse Osteopenia by Promoting Mesenchymal Stem Cell Osteogenic Differentiation and Osteogenesis in Mice. Stem Cell. Res. Ther. 11 (1), 58. 10.1186/s13287-020-1581-6 32054528 PMC7020585

[B68] Huang C.-C. Kang M. Lu Y. Shirazi S. Diaz J. I. Cooper L. F. (2020). Functionally Engineered Extracellular Vesicles Improve Bone Regeneration. Acta Biomater. 109, 182–194. 10.1016/j.actbio.2020.04.017 32305445 PMC8040700

[B69] Huang J.-Z. Chen M. Chen D. Gao X.-C. Zhu S. Huang H. (2017). A Peptide Encoded by a Putative lncRNA HOXB-AS3 Suppresses Colon Cancer Growth. Mol. Cell. 68 (1), 171–184. 10.1016/j.molcel.2017.09.015 28985503

[B70] Huang J. Chen L. (2017). IL-1β Inhibits Osteogenesis of Human Bone Marrow-Derived Mesenchymal Stem Cells by Activating FoxD3/microRNA-496 to Repress Wnt Signaling. Genesis 55 (7), e23040. 10.1002/dvg.23040 28509407

[B71] Huang J. Meng Y. Liu Y. Chen Y. Yang H. Chen D. (2016). MicroRNA-320a Regulates the Osteogenic Differentiation of Human Bone Marrow-Derived Mesenchymal Stem Cells by Targeting HOXA10. Cell. Physiol. Biochem. 38 (1), 40–48. 10.1159/000438607 26741129

[B72] Huang S. Yang B. Chen B. J. Bliim N. Ueberham U. Arendt T. (2017). The Emerging Role of Circular RNAs in Transcriptome Regulation. Genomics 109 (5-6), 401–407. 10.1016/j.ygeno.2017.06.005 28655641

[B73] Huang X.-Z. Huang J. Li W.-Z. Wang J.-J. Song D.-Y. Ni J.-D. (2020). LncRNA-MALAT1 Promotes Osteogenic Differentiation through Regulating ATF4 by Sponging miR-214: Implication of Steroid-Induced Avascular Necrosis of the Femoral Head. Steroids 154, 108533. 10.1016/j.steroids.2019.108533 31678133

[B74] Huang Y. Xu Y. Feng S. He P. Sheng B. Ni J. (2021). miR-19b Enhances Osteogenic Differentiation of Mesenchymal Stem Cells and Promotes Fracture Healing through the WWP1/Smurf2-Mediated KLF5/β-Catenin Signaling Pathway. Exp. Mol. Med. 53 (5), 973–985. 10.1038/s12276-021-00631-w 34035464 PMC8178348

[B75] Huang Y. Zheng Y. Jia L. Li W. (2015). Long Noncoding RNA H19 Promotes Osteoblast Differentiation via TGF-β1/Smad3/HDAC Signaling Pathway by Deriving miR-675. STEM CELLS 33 (12), 3481–3492. 10.1002/stem.2225 26417995

[B76] Huang Z. Cai Z. Qian J. Wang J. Hu N. (2020). Effect of Micro RNA-335-5p Regulating Bone Morphogenetic Protein 2 on Osteogenic Differentiation of Human Bone Marrow Mesenchymal Stem Cells. Zhongguo Xiu Fu Chong Jian Wai Ke Za Zhi 34 (6), 781–786. 10.7507/1002-1892.201910097 32538572 PMC8171532

[B77] Huntzinger E. Izaurralde E. (2011). Gene Silencing by microRNAs: Contributions of Translational Repression and mRNA Decay. Nat. Rev. Genet. 12 (2), 99–110. 10.1038/nrg2936 21245828

[B78] Huo S. Zhou Y. He X. Wan M. Du W. Xu X. (2017). Insight into the Role of Long Non-coding RNAs during Osteogenesis in Mesenchymal Stem Cells. Cscr 13 (1), 52–59. 10.2174/1574888x12666171115124112 29141554

[B79] Iwasaki Y. W. Siomi M. C. Siomi H. (2015). PIWI-interacting RNA: Its Biogenesis and Functions. Annu. Rev. Biochem. 84, 405–433. 10.1146/annurev-biochem-060614-034258 25747396

[B80] Ji Z. Song R. Regev A. Struhl K. (2015). Many lncRNAs, 5'UTRs, and Pseudogenes Are Translated and Some Are Likely to Express Functional Proteins. Elife 4, e08890. 10.7554/eLife.08890 26687005 PMC4739776

[B81] Jia B. Wang Z. Sun X. Chen J. Zhao J. Qiu X. (2019). Long Noncoding RNA LINC00707 Sponges miR-370-3p to Promote Osteogenesis of Human Bone Marrow-Derived Mesenchymal Stem Cells through Upregulating WNT2B. Stem Cell. Res. Ther. 10 (1), 67. 10.1186/s13287-019-1161-9 30795799 PMC6387535

[B82] Jiang K. Teng G. D. Chen Y. Q. (2020). MicroRNA‐23 Suppresses Osteogenic Differentiation of Human Bone Marrow Mesenchymal Stem Cells by Targeting the MEF2C‐mediated MAPK Signaling Pathway. J. Gene Med. 22 (10), e3216. 10.1002/jgm.3216 32410261

[B83] Jiang L. B. Tian L. Zhang C. G. (2018). Bone Marrow Stem Cells-Derived Exosomes Extracted from Osteoporosis Patients Inhibit Osteogenesis via microRNA-21/SMAD7. Eur. Rev. Med. Pharmacol. Sci. 22 (19), 6221–6229. 10.26355/eurrev_201810_16028 30338786

[B84] Jiang Y. Zhang P. Zhang X. Lv L. Zhou Y. (2021). Advances in Mesenchymal Stem Cell Transplantation for the Treatment of Osteoporosis. Cell. Prolif. 54 (1), e12956. 10.1111/cpr.12956 33210341 PMC7791182

[B85] Jiang Z.-F. Zhang L. (2021). LncRNA: A Potential Research Direction in Intestinal Barrier Function. Dig. Dis. Sci. 66 (5), 1400–1408. 10.1007/s10620-020-06417-w 32591966

[B86] Jin C. Jia L. Tang Z. Zheng Y. (2020). Long Non-coding RNA MIR22HG Promotes Osteogenic Differentiation of Bone Marrow Mesenchymal Stem Cells via PTEN/AKT Pathway. Cell. Death Dis. 11 (7), 601. 10.1038/s41419-020-02813-2 32732881 PMC7393093

[B87] Jin Y. Hong F. Bao Q. Xu Q. Duan R. Zhu Z. (2020). MicroRNA-145 Suppresses Osteogenic Differentiation of Human Jaw Bone Marrow Mesenchymal Stem Cells Partially via Targeting Semaphorin 3A. Connect. Tissue Res. 61 (6), 577–585. 10.1080/03008207.2019.1643334 31305177

[B88] Kalluri R. LeBleu V. S. (2020). The Biology , Function , and Biomedical Applications of Exosomes. Science 367 (6478), eaau6977. 10.1126/science.aau6977 32029601 PMC7717626

[B89] Karijolich J. Yu Y.-T. (2010). Spliceosomal snRNA Modifications and Their Function. RNA Biol. 7 (2), 192–204. 10.4161/rna.7.2.11207 20215871 PMC4154345

[B90] Khan M. A. F. Reckman Y. J. Aufiero S. van den Hoogenhof M. M. G. van der Made I. Beqqali A. (2016). RBM20 Regulates Circular RNA Production from the Titin Gene. Circ. Res. 119 (9), 996–1003. 10.1161/circresaha.116.309568 27531932

[B91] Kim H. K. Fuchs G. Wang S. Wei W. Zhang Y. Park H. (2017). A Transfer-RNA-Derived Small RNA Regulates Ribosome Biogenesis. Nature 552 (7683), 57–62. 10.1038/nature25005 29186115 PMC6066594

[B92] Kim K. M. Park S. J. Jung S.-H. Kim E. J. Jogeswar G. Ajita J. (2012). miR-182 Is a Negative Regulator of Osteoblast Proliferation, Differentiation, and Skeletogenesis through Targeting FoxO1. J. Bone Min. Res. 27 (8), 1669–1679. 10.1002/jbmr.1604 22431396

[B93] Kitamura Y. I. Kitamura T. Kruse J.-P. Raum J. C. Stein R. Gu W. (2005). FoxO1 Protects against Pancreatic β Cell Failure through NeuroD and MafA Induction. Cell. Metab. 2 (3), 153–163. 10.1016/j.cmet.2005.08.004 16154098

[B94] Kong R. Gao J. Ji L. Zhao D. (2020). MicroRNA-126 Promotes Proliferation, Migration, Invasion and Endothelial Differentiation while Inhibits Apoptosis and Osteogenic Differentiation of Bone Marrow-Derived Mesenchymal Stem Cells. Cell. Cycle 19 (17), 2119–2138. 10.1080/15384101.2020.1788258 32787491 PMC7513857

[B95] Kristensen L. S. Andersen M. S. Stagsted L. V. W. Ebbesen K. K. Hansen T. B. Kjems J. (2019). The Biogenesis, Biology and Characterization of Circular RNAs. Nat. Rev. Genet. 20 (11), 675–691. 10.1038/s41576-019-0158-7 31395983

[B96] Lee R. C. Feinbaum R. L. Ambros V. (1993). The *C. elegans* Heterochronic Gene Lin-4 Encodes Small RNAs with Antisense Complementarity to Lin-14. Cell. 75 (5), 843–854. 10.1016/0092-8674(93)90529-y 8252621

[B97] Lei N. B. Liang X. Wang P. Liu Q. Wang W. G. (2019). Teriparatide Alleviates Osteoporosis by Promoting Osteogenic Differentiation of hMSCs via miR-375/RUNX2 axis. Eur. Rev. Med. Pharmacol. Sci. 23 (24), 11043–11050. 10.26355/eurrev_201912_19812 31858576

[B98] Li D. Taylor D. H. van Wolfswinkel J. C. (2021). PIWI-mediated Control of Tissue-specific Transposons Is Essential for Somatic Cell Differentiation. Cell. Rep. 37 (1), 109776. 10.1016/j.celrep.2021.109776 34610311 PMC8532177

[B99] Li H. Xu J.-D. Fang X.-H. Zhu J.-N. Yang J. Pan R. (2020). Circular RNA circRNA_000203 Aggravates Cardiac Hypertrophy via Suppressing miR-26b-5p and miR-140-3p Binding to Gata4. Cardiovasc Res. 116 (7), 1323–1334. 10.1093/cvr/cvz215 31397837 PMC7243276

[B100] Li H. Xu X. Wang D. Zhang Y. Chen J. Li B. (2021). Hypermethylation-mediated Downregulation of Long Non-coding RNA MEG3 Inhibits Osteogenic Differentiation of Bone Marrow Mesenchymal Stem Cells and Promotes Pediatric Aplastic Anemia. Int. Immunopharmacol. 93, 107292. 10.1016/j.intimp.2020.107292 33529912

[B101] Li I. Chen Y. G. (2021). Emerging Roles of Circular RNAs in Innate Immunity. Curr. Opin. Immunol. 68, 107–115. 10.1016/j.coi.2020.10.010 33176221 PMC7925352

[B102] Li J. J. Ebied M. Xu J. Zreiqat H. (2018). Current Approaches to Bone Tissue Engineering: The Interface between Biology and Engineering. Adv. Healthc. Mater 7 (6), e1701061. ARTN 1701061. 10.1002/adhm.201701061 29280321

[B103] Li J. Shi Q. Wang Q. Tan X. Pang K. Liu X. (2019). Profiling Circular RNA in Methamphetamine-Treated Primary Cortical Neurons Identified Novel circRNAs Related to Methamphetamine Addiction. Neurosci. Lett. 701, 146–153. 10.1016/j.neulet.2019.02.032 30797870

[B104] Li J. Wu X. Shi Y. Zhao H. (2021). FGD5-AS1 Facilitates the Osteogenic Differentiation of Human Bone Marrow-Derived Mesenchymal Stem Cells via Targeting the miR-506-3p/BMP7 axis. J. Orthop. Surg. Res. 16 (1), 665. 10.1186/s13018-021-02694-x 34772438 PMC8588622

[B105] Li N. Liu L. Liu Y. Luo S. Song Y. Fang B. (2020). miR-144-3p Suppresses Osteogenic Differentiation of BMSCs from Patients with Aplastic Anemia through Repression of TET2. Mol. Ther. - Nucleic Acids 19, 619–626. 10.1016/j.omtn.2019.12.017 31945725 PMC6965517

[B106] Li R. Ruan Q. Yin F. Zhao K. (2021). MiR-23b-3p Promotes Postmenopausal Osteoporosis by Targeting MRC2 and Regulating the Wnt/β-Catenin Signaling Pathway. J. Pharmacol. Sci. 145 (1), 69–78. 10.1016/j.jphs.2020.11.004 33357782

[B107] Li T. Li H. Li T. Fan J. Zhao R. C. Weng X. (2014). MicroRNA Expression Profile of Dexamethasone-Induced Human Bone Marrow-Derived Mesenchymal Stem Cells during Osteogenic Differentiation. J. Cell. Biochem. 115 (10), 1683–1691. 10.1002/jcb.24831 24802236

[B108] Li X. Yang L. Chen L.-L. (2018). The Biogenesis, Functions, and Challenges of Circular RNAs. Mol. Cell. 71 (3), 428–442. 10.1016/j.molcel.2018.06.034 30057200

[B109] Li Y. Jiang J. Liu W. Wang H. Zhao L. Liu S. (2018). microRNA-378 Promotes Autophagy and Inhibits Apoptosis in Skeletal Muscle. Proc. Natl. Acad. Sci. U.S.A. 115 (46), E10849–e10858. 10.1073/pnas.1803377115 30373812 PMC6243236

[B110] Li Y. Wang J. Ma Y. Du W. Feng K. Wang S. (2021). miR‐101‐loaded Exosomes Secreted by Bone Marrow Mesenchymal Stem Cells Requires the FBXW7/HIF1α/FOXP3 axis, Facilitating Osteogenic Differentiation. J. Cell. Physiol. 236 (6), 4258–4272. 10.1002/jcp.30027 33438204

[B111] Lin K.-Y. Wang W.-D. Lin C.-H. Rastegari E. Su Y.-H. Chang Y.-T. (2020). Piwi Reduction in the Aged Niche Eliminates Germline Stem Cells via Toll-GSK3 Signaling. Nat. Commun. 11 (1), 3147. 10.1038/s41467-020-16858-6 32561720 PMC7305233

[B112] Lin S. Gregory R. I. (2015). MicroRNA Biogenesis Pathways in Cancer. Nat. Rev. Cancer 15 (6), 321–333. 10.1038/nrc3932 25998712 PMC4859809

[B113] Lin Y. P. Liao L. M. Liu Q. H. Ni Y. Zhong Y. Yu S. (2021). MiRNA-128-3p Induces Osteogenic Differentiation of Bone Marrow Mesenchymal Stem Cells via Activating the Wnt3a Signaling. Eur. Rev. Med. Pharmacol. Sci. 25 (3), 1225–1232. 10.26355/eurrev_202102_24826 33629292

[B114] Lin Z. He H. Wang M. Liang J. (2019). MicroRNA‐130a Controls Bone Marrow Mesenchymal Stem Cell Differentiation towards the Osteoblastic and Adipogenic Fate. Cell. Prolif. 52 (6), e12688. 10.1111/cpr.12688 31557368 PMC6869834

[B115] Liu B. Cao J. Wang X. Guo C. Liu Y. Wang T. (2021a). Deciphering the tRNA-Derived Small RNAs: Origin, Development, and Future. Cell. Death Dis. 13 (1), 24. 10.1038/s41419-021-04472-3 34934044 PMC8692627

[B116] Liu B. Gan W. Jin Z. Wang M. Cui G. Zhang H. (2021b). The Role of miR-34c-5p in Osteogenic Differentiation of Bone Marrow Mesenchymal Stem Cells. Ijsc 14 (3), 286–297. 10.15283/ijsc20188 PMC8429940 33906980

[B117] Liu C. Liu A.-S. Zhong D. Wang C.-G. Yu M. Zhang H.-W. (2021). Circular RNA AFF4 Modulates Osteogenic Differentiation in BM-MSCs by Activating SMAD1/5 Pathway through miR-135a-5p/FNDC5/Irisin axis. Cell. Death Dis. 12 (7), 631. 10.1038/s41419-021-03877-4 34145212 PMC8213698

[B118] Liu H. Zhong L. Yuan T. Chen S. Zhou Y. An L. (2018). MicroRNA-155 Inhibits the Osteogenic Differentiation of Mesenchymal Stem Cells Induced by BMP9 via Downregulation of BMP Signaling Pathway. Int. J. Mol. Med. 41 (6), 3379–3393. 10.3892/ijmm.2018.3526 29512689 PMC5881775

[B119] Liu J. Chen M. Ma L. Dang X. Du G. (2021). piRNA-36741 Regulates BMP2-Mediated Osteoblast Differentiation via METTL3 Controlled m6A Modification. Aging 13 (19), 23361–23375. 10.18632/aging.203630 34645714 PMC8544320

[B120] Liu L. Zeng D. Chen Y. Zhou J. Liao Y. Shi B. (2020). Microarc Oxidation Surface of Titanium Implants Promote Osteogenic Differentiation by Activating ERK1/2-miR-1827-Osterix. Vitro Cell.Dev.Biol.-Animal 56 (4), 296–306. 10.1007/s11626-020-00444-7 32270391

[B121] Liu N. Kataoka M. Wang Y. Pu L. Dong X. Fu X. (2021). LncRNA LncHrt Preserves Cardiac Metabolic Homeostasis and Heart Function by Modulating the LKB1-AMPK Signaling Pathway. Basic Res. Cardiol. 116 (1), 48. 10.1007/s00395-021-00887-3 34379189 PMC8357683

[B122] Liu R. Li Z. Song E. Hu P. Yang Q. Hu Y. (2020). LncRNA HOTTIP Enhances Human Osteogenic BMSCs Differentiation via Interaction with WDR5 and Activation of Wnt/β-Catenin Signalling Pathway. Biochem. Biophysical Res. Commun. 524 (4), 1037–1043. 10.1016/j.bbrc.2020.02.034 32067741

[B123] Liu S. Yang X. Zhong X. Li L. Zhang X. (2021). Involvement of miR-337 in High Glucose-Suppressed Osteogenic Differentiation in Bone Marrow Mesenchymal Stem Cells via Negative Regulation of Rap1A. Vitro Cell.Dev.Biol.-Animal 57 (3), 350–358. 10.1007/s11626-021-00553-x 33748908

[B124] Liu W. Zhang J. Zou C. Xie X. Wang Y. Wang B. (2017). Microarray Expression Profile and Functional Analysis of Circular RNAs in Osteosarcoma. Cell. Physiol. Biochem. 43 (3), 969–985. 10.1159/000481650 28957794

[B125] Liu Y. Wang Y. Cheng X. Zheng Y. Lyu M. Di P. (2021). MiR-181d-5p Regulates Implant Surface Roughness-Induced Osteogenic Differentiation of Bone Marrow Stem Cells. Mater. Sci. Eng. C 121, 111801. 10.1016/j.msec.2020.111801 33579448

[B126] Liu Z. H. Qi D. D. Li X. Zhang S. Q. Zhao Y. Fu L. X. (2021). LncRNA SNHG14 Promotes Osteogenic Differentiation of Human Bone Marrow-Derived Mesenchymal Stem Cells via Regulating miR-185-5p/WISP2 axis. J. Biol. Regul. Homeost. Agents 35 (2), 605–615. 10.23812/20-391-a 33928771

[B127] Long C. Cen S. Zhong Z. Zhou C. Zhong G. (2021). FOXO3 Is Targeted by miR-223-3p and Promotes Osteogenic Differentiation of Bone Marrow Mesenchymal Stem Cells by Enhancing Autophagy. Hum. Cell. 34 (1), 14–27. 10.1007/s13577-020-00421-y 32920731 PMC7788031

[B128] Long H. Sun B. Cheng L. Zhao S. Zhu Y. Zhao R. (2017). miR-139-5p Represses BMSC Osteogenesis via Targeting Wnt/β-Catenin Signaling Pathway. DNA Cell. Biol. 36 (8), 715–724. 10.1089/dna.2017.3657 28622009

[B129] Long H. Zhu Y. Lin Z. Wan J. Cheng L. Zeng M. (2019). miR-381 Modulates Human Bone Mesenchymal Stromal Cells (BMSCs) Osteogenesis via Suppressing Wnt Signaling Pathway during Atrophic Nonunion Development. Cell. Death Dis. 10 (7), 470. 10.1038/s41419-019-1693-z 31209205 PMC6572824

[B130] Lu T. X. Rothenberg M. E. (2018). MicroRNA. J. Allergy Clin. Immunol. 141 (4), 1202–1207. 10.1016/j.jaci.2017.08.034 29074454 PMC5889965

[B131] Lu X. Deng M. He H. Zeng D. Zhang W. (2013). [miR-125b Regulates Osteogenic Differentiation of Human Bone Marrow Mesenchymal Stem Cells by Targeting Smad4]. Zhong Nan Da Xue Xue Bao Yi Xue Ban. 38 (4), 341–346. 10.3969/j.issn.1672-7347.2013.04.002 23645233

[B132] Luo B. Yang J. F. Wang Y. H. Qu G. B. Hao P. D. Zeng Z. J. (2019). MicroRNA-579-3p Promotes the Progression of Osteoporosis by Inhibiting Osteogenic Differentiation of Mesenchymal Stem Cells through Regulating Sirt1. Eur. Rev. Med. Pharmacol. Sci. 23 (16), 6791–6799. 10.26355/eurrev_201908_18717 31486477

[B133] Luo Z. Shang X. Zhang H. Wang G. Massey P. A. Barton S. R. (2019). Notch Signaling in Osteogenesis, Osteoclastogenesis, and Angiogenesis. Am. J. Pathology 189 (8), 1495–1500. 10.1016/j.ajpath.2019.05.005 PMC6699068 31345466

[B134] Lv H. Yang H. Wang Y. (2020). Effects of miR-103 by Negatively Regulating SATB2 on Proliferation and Osteogenic Differentiation of Human Bone Marrow Mesenchymal Stem Cells. PLoS One 15 (5), e0232695. 10.1371/journal.pone.0232695 32379794 PMC7205233

[B135] Lv R. Pan X. Song L. Sun Q. Guo C. Zou S. (2019). MicroRNA-200a-3p Accelerates the Progression of Osteoporosis by Targeting Glutaminase to Inhibit Osteogenic Differentiation of Bone Marrow Mesenchymal Stem Cells. Biomed. Pharmacother. 116, 108960. 10.1016/j.biopha.2019.108960 31112871

[B136] Ma K. Song G. An X. Fan A. Tan W. Tang B. (2014). miRNAs Promote Generation of Porcine-Induced Pluripotent Stem Cells. Mol. Cell. Biochem. 389 (1-2), 209–218. 10.1007/s11010-013-1942-x 24464032

[B137] Macfarlane L.-A. R. Murphy P. (2010). MicroRNA: Biogenesis, Function and Role in Cancer. Cg 11 (7), 537–561. 10.2174/138920210793175895 PMC3048316 21532838

[B138] Mazziotta C. Lanzillotti C. Iaquinta M. R. Taraballi F. Torreggiani E. Rotondo J. C. (2021). MicroRNAs Modulate Signaling Pathways in Osteogenic Differentiation of Mesenchymal Stem Cells. Ijms 22 (5), 2362. 10.3390/ijms22052362 33673409 PMC7956574

[B139] McCabe E. M. Rasmussen T. P. (2021). lncRNA Involvement in Cancer Stem Cell Function and Epithelial-Mesenchymal Transitions. Seminars Cancer Biol. 75, 38–48. 10.1016/j.semcancer.2020.12.012 33346133

[B140] Mei B. Wang Y. Ye W. Huang H. Zhou Q. Chen Y. (2019). LncRNA ZBTB40-IT1 Modulated by Osteoporosis GWAS Risk SNPs Suppresses Osteogenesis. Hum. Genet. 138 (2), 151–166. 10.1007/s00439-019-01969-y 30661131

[B141] Mi B. Xiong Y. Chen L. Yan C. Endo Y. Liu Y. (2019). CircRNA AFF4 Promotes Osteoblast Cells Proliferation and Inhibits Apoptosis via the Mir-7223-5p/PIK3R1 axis. Aging 11 (24), 11988–12001. 10.18632/aging.102524 31848327 PMC6949079

[B142] Michael A. Bajracharya S. Yuen P. Zhou H. Star R. Illei G. (2010). Exosomes from Human Saliva as a Source of microRNA Biomarkers. Oral Dis. 16 (1), 34–38. 10.1111/j.1601-0825.2009.01604.x 19627513 PMC2844919

[B143] Mollazadeh S. Fazly Bazzaz B. S. Neshati V. de Vries A. A. F. Naderi-Meshkin H. Mojarad M. (2019). Overexpression of MicroRNA-148b-3p Stimulates Osteogenesis of Human Bone Marrow-Derived Mesenchymal Stem Cells: the Role of MicroRNA-148b-3p in Osteogenesis. BMC Med. Genet. 20 (1), 117. 10.1186/s12881-019-0854-3 31262253 PMC6604430

[B144] Morgado A. L. Rodrigues C. M. P. Solá S. (2016). MicroRNA-145 Regulates Neural Stem Cell Differentiation through the Sox2-Lin28/let-7 Signaling Pathway. Stem Cells 34 (5), 1386–1395. 10.1002/stem.2309 26849971

[B145] Narayanan A. Srinaath N. Rohini M. Selvamurugan N. (2019). Regulation of Runx2 by MicroRNAs in Osteoblast Differentiation. Life Sci. 232, 116676. 10.1016/j.lfs.2019.116676 31340165

[B146] Ng F. Boucher S. Koh S. Sastry K. S. R. Chase L. Lakshmipathy U. (2008). PDGF, TGF-β, and FGF Signaling Is Important for Differentiation and Growth of Mesenchymal Stem Cells (MSCs): Transcriptional Profiling Can Identify Markers and Signaling Pathways Important in Differentiation of MSCs into Adipogenic, Chondrogenic, and Osteogenic Lineages. Blood 112 (2), 295–307. 10.1182/blood-2007-07-103697 18332228

[B147] Nicot R. Schlund M. Touzet-Roumazeille S. Ferri J. Raoul G. (2020). Unicortical Calvarial Autologous Bone Graft Harvest. Plast. Reconstr. Surg. Glob. Open 8 (11), e3241. 10.1097/gox.0000000000003241 33299707 PMC7722572

[B148] Nielsen-Preiss S. M. Silva S. R. Gillette J. M. (2003). Role of PTEN and Akt in the Regulation of Growth and Apoptosis in Human Osteoblastic Cells. J. Cell. Biochem. 90 (5), 964–975. 10.1002/jcb.10709 14624456

[B149] Niu L. Lou F. Sun Y. Sun L. Cai X. Liu Z. (2020). A Micropeptide Encoded by lncRNA MIR155HG Suppresses Autoimmune Inflammation via Modulating Antigen Presentation. Sci. Adv. 6 (21), eaaz2059. 10.1126/sciadv.aaz2059 32671205 PMC7314557

[B150] Oskowitz A. Z. Lu J. Penfornis P. Ylostalo J. McBride J. Flemington E. K. (2008). Human Multipotent Stromal Cells from Bone Marrow and microRNA: Regulation of Differentiation and Leukemia Inhibitory Factor Expression. Proc. Natl. Acad. Sci. U.S.A. 105 (47), 18372–18377. 10.1073/pnas.0809807105 19011087 PMC2587615

[B151] Ouyang Q. Wu J. Jiang Z. Zhao J. Wang R. Lou A. (2017). Microarray Expression Profile of Circular RNAs in Peripheral Blood Mononuclear Cells from Rheumatoid Arthritis Patients. Cell. Physiol. Biochem. 42 (2), 651–659. 10.1159/000477883 28618429

[B152] Ouyang Z. Tan T. Zhang X. Wan J. Zhou Y. Jiang G. (2019). CircRNA Hsa_circ_0074834 Promotes the Osteogenesis-Angiogenesis Coupling Process in Bone Mesenchymal Stem Cells (BMSCs) by Acting as a ceRNA for miR-942-5p. Cell. Death Dis. 10 (12), 932. 10.1038/s41419-019-2161-5 31804461 PMC6895238

[B153] Ouyang Z. Tan T. Zhang X. Wan J. Zhou Y. Jiang G. (2020). LncRNA ENST00000563492 Promoting the Osteogenesis-Angiogenesis Coupling Process in Bone Mesenchymal Stem Cells (BMSCs) by Functions as a ceRNA for miR-205-5p. Cell. Death Dis. 11 (6), 486. 10.1038/s41419-020-2689-4 32587236 PMC7316863

[B154] Patop I. L. Wüst S. Kadener S. (2019). Past, Present, and Future of Circ RNA S. Embo J. 38 (16), e100836. 10.15252/embj.2018100836 31343080 PMC6694216

[B155] Peng F. Gong W. Li S. Yin B. Zhao C. Liu W. (2021). circRNA_010383 Acts as a Sponge for miR-135a, and its Downregulated Expression Contributes to Renal Fibrosis in Diabetic Nephropathy. Diabetes 70 (2), 603–615. 10.2337/db20-0203 33472945

[B156] Peng S. Cao L. He S. Zhong Y. Ma H. Zhang Y. (20182018). An Overview of Long Noncoding RNAs Involved in Bone Regeneration from Mesenchymal Stem Cells. Stem Cells Int. 2018, 1–11. 10.1155/2018/8273648 PMC5829309 29535782

[B157] Peng W. Zhu S. Chen J. Wang J. Rong Q. Chen S. (2019). Hsa_circRNA_33287 Promotes the Osteogenic Differentiation of Maxillary Sinus Membrane Stem Cells via miR-214-3p/Runx3. Biomed. Pharmacother. 109, 1709–1717. 10.1016/j.biopha.2018.10.159 30551425

[B158] Ponting C. P. Oliver P. L. Reik W. (2009). Evolution and Functions of Long Noncoding RNAs. Cell. 136 (4), 629–641. 10.1016/j.cell.2009.02.006 19239885

[B159] Qadir A. S. Um S. Lee H. Baek K. Seo B. M. Lee G. (2015). miR-124 Negatively Regulates Osteogenic Differentiation and *In Vivo* Bone Formation of Mesenchymal Stem Cells. J. Cell. Biochem. 116 (5), 730–742. 10.1002/jcb.25026 25424317

[B160] Qi M. Zhang L. Ma Y. Shuai Y. Li L. Luo K. (2017). Autophagy Maintains the Function of Bone Marrow Mesenchymal Stem Cells to Prevent Estrogen Deficiency-Induced Osteoporosis. Theranostics 7 (18), 4498–4516. 10.7150/thno.17949 29158841 PMC5695145

[B161] Qiao L. Liu D. Li C. G. Wang Y. J. (2018). MiR-203 Is Essential for the Shift from Osteogenic Differentiation to Adipogenic Differentiation of Mesenchymal Stem Cells in Postmenopausal Osteoporosis. Eur. Rev. Med. Pharmacol. Sci. 22 (18), 5804–5814. 10.26355/eurrev_201809_15906 30280759

[B162] Qiu Y. Chen Y. Zeng T. Guo W. Zhou W. Yang X. (2016). EGCG Ameliorates the Hypoxia-Induced Apoptosis and Osteogenic Differentiation Reduction of Mesenchymal Stem Cells via Upregulating miR-210. Mol. Biol. Rep. 43 (3), 183–193. 10.1007/s11033-015-3936-0 26780211

[B163] Raposo G. Stahl P. D. (2019). Extracellular Vesicles: a New Communication Paradigm? Nat. Rev. Mol. Cell. Biol. 20 (9), 509–510. 10.1038/s41580-019-0158-7 31324871

[B164] Raposo G. Stoorvogel W. (2013). Extracellular Vesicles: Exosomes, Microvesicles, and Friends. J. Cell. Biol. 200 (4), 373–383. 10.1083/jcb.201211138 23420871 PMC3575529

[B165] Ren G. Sun J. Li M. M. Zhang Y. D. Li R. H. Li Y. M. (2018). MicroRNA-23a-5p Regulates Osteogenic Differentiation of Human Bone Marrow-Derived Mesenchymal Stem Cells by Targeting Mitogen-Activated Protein Kinase-13. Mol. Med. Rep. 17 (3), 4554–4560. 10.3892/mmr.2018.8452 29344643

[B166] Seenprachawong K. Tawornsawutruk T. Nantasenamat C. Nuchnoi P. Hongeng S. Supokawej A. (2018). miR-130a and miR-27b Enhance Osteogenesis in Human Bone Marrow Mesenchymal Stem Cells via Specific Down-Regulation of Peroxisome Proliferator-Activated Receptor γ. Front. Genet. 9, 543. 10.3389/fgene.2018.00543 30487813 PMC6246628

[B167] Sha Y. Lv Y. Xu Z. Yang L. Hao X. Afandi R. (2017). MGF E Peptide Pretreatment Improves the Proliferation and Osteogenic Differentiation of BMSCs via MEK-Erk1/2 and PI3K-Akt Pathway under Severe Hypoxia. Life Sci. 189, 52–62. 10.1016/j.lfs.2017.09.017 28927682

[B168] Shen J. J. Zhang C. H. Chen Z. W. Wang Z. X. Yang D. C. Zhang F. L. (2019). LncRNA HOTAIR Inhibited Osteogenic Differentiation of BMSCs by Regulating Wnt/β-Catenin Pathway. Eur. Rev. Med. Pharmacol. Sci. 23 (17), 7232–7246. 10.26355/eurrev_201909_18826 31539110

[B169] Shen S. Wu Y. Chen J. Xie Z. Huang K. Wang G. (2019). CircSERPINE2 Protects against Osteoarthritis by Targeting miR-1271 and ETS-Related Gene. Ann. Rheum. Dis. 78 (6), 826–836. 10.1136/annrheumdis-2018-214786 30923232 PMC6579553

[B170] Shen W. Sun B. Zhou C. Ming W. Zhang S. Wu X. (2020). CircFOXP1/FOXP1 Promotes Osteogenic Differentiation in Adipose‐derived Mesenchymal Stem Cells and Bone Regeneration in Osteoporosis via miR‐33a‐5p. J. Cell. Mol. Med. 24 (21), 12513–12524. 10.1111/jcmm.15792 32996692 PMC7687013

[B171] Shermane Lim Y. W. Xiang X. Garg M. Le M. T. Li-Ann Wong A. Wang L. (2021). The Double-Edged Sword of H19 lncRNA: Insights into Cancer Therapy. Cancer Lett. 500, 253–262. 10.1016/j.canlet.2020.11.006 33221454

[B172] Shi Y. Fang N. Li Y. Guo Z. Jiang W. He Y. (2020). Circular RNA LPAR3 Sponges microRNA‐198 to Facilitate Esophageal Cancer Migration, Invasion, and Metastasis. Cancer Sci. 111 (8), 2824–2836. 10.1111/cas.14511 32495982 PMC7419039

[B173] Simic P. Zainabadi K. Bell E. Sykes D. B. Saez B. Lotinun S. (2013). SIRT1 Regulates Differentiation of Mesenchymal Stem Cells by Deacetylating β‐catenin. EMBO Mol. Med. 5 (3), 430–440. 10.1002/emmm.201201606 23364955 PMC3598082

[B174] Sinha K. M. Zhou X. (2013). Genetic and Molecular Control of Osterix in Skeletal Formation. J. Cell. Biochem. 114 (5), 975–984. 10.1002/jcb.24439 23225263 PMC3725781

[B175] Skillington J. Choy L. Derynck R. (2002). Bone Morphogenetic Protein and Retinoic Acid Signaling Cooperate to Induce Osteoblast Differentiation of Preadipocytes. J. Cell. Biol. 159 (1), 135–146. 10.1083/jcb.200204060 12379805 PMC2173483

[B176] Sloan K. E. Warda A. S. Sharma S. Entian K.-D. Lafontaine D. L. J. Bohnsack M. T. (2017). Tuning the Ribosome: The Influence of rRNA Modification on Eukaryotic Ribosome Biogenesis and Function. RNA Biol. 14 (9), 1138–1152. 10.1080/15476286.2016.1259781 27911188 PMC5699541

[B177] Sohn H.-S. Oh J.-K. (2019). Review of Bone Graft and Bone Substitutes with an Emphasis on Fracture Surgeries. Biomater. Res. 23, 9. 10.1186/s40824-019-0157-y 30915231 PMC6417250

[B178] Song G. Shen Y. Ruan Z. Li X. Chen Y. Yuan W. (2016). LncRNA-uc.167 Influences Cell Proliferation, Apoptosis and Differentiation of P19 Cells by Regulating Mef2c. Gene 590 (1), 97–108. 10.1016/j.gene.2016.06.006 27268728

[B179] Steinhardt Y. Aslan H. Regev E. Zilberman Y. Kallai I. Gazit D. (2008). Maxillofacial-derived Stem Cells Regenerate Critical Mandibular Bone Defect. Tissue Eng. Part A 14 (11), 1763–1773. 10.1089/ten.tea.2008.0007 18636943

[B180] Su P.-H. Hsu Y.-W. Huang R.-L. Chen L.-Y. Chao T.-K. Liao C.-C. (2019). TET1 Promotes 5hmC-dependent Stemness, and Inhibits a 5hmC-independent Epithelial-Mesenchymal Transition, in Cervical Precancerous Lesions. Cancer Lett. 450, 53–62. 10.1016/j.canlet.2019.01.033 30771438

[B181] Sun J. Wang Y. Li Y. Zhao G. (2014). Downregulation of PPARγ by miR-548d-5p Suppresses the Adipogenic Differentiation of Human Bone Marrow Mesenchymal Stem Cells and Enhances Their Osteogenic Potential. J. Transl. Med. 12, 168. 10.1186/1479-5876-12-168 24929254 PMC4068155

[B182] Sun Z. Zhang Q. Yuan W. Li X. Chen C. Guo Y. (2020a). MiR-103a-3p Promotes Tumour Glycolysis in Colorectal Cancer via hippo/YAP1/HIF1A axis. J. Exp. Clin. Cancer Res. 39 (1), 250. 10.1186/s13046-020-01705-9 33218358 PMC7678148

[B183] Sun Z. Wu F. Yang Y. Liu F. Mo F. Chen J. (2020b). MiR-144-3p Inhibits BMSC Proliferation and Osteogenic Differentiation via Targeting FZD4 in Steroid-Associated Osteonecrosis. Cpd 25 (45), 4806–4812. 10.2174/1381612825666190930094019 31566128

[B184] Tang J. Yu H. Wang Y. Duan G. Wang B. Li W. (2021). miR-27a Promotes Osteogenic Differentiation in Glucocorticoid-Treated Human Bone Marrow Mesenchymal Stem Cells by Targeting PI3K. J. Mol. Histol. 52 (2), 279–288. 10.1007/s10735-020-09947-9 33532936

[B185] Tang J. Z. Lin X. Zhong J. Y. Xu F. Wu F. Liao X. B. (2019). miR-124 Regulates the Osteogenic Differentiation of Bone Marrow-derived Mesenchymal Stem Cells by Targeting Sp7. Mol. Med. Rep. 19 (5), 3807–3814. 10.3892/mmr.2019.10054 30896834

[B186] Tang S. a. Xie Z. Wang P. Li J. Wang S. Liu W. (2019). LncRNA-OG Promotes the Osteogenic Differentiation of Bone Marrow-Derived Mesenchymal Stem Cells under the Regulation of hnRNPK. Stem Cells 37 (2), 270–283. 10.1002/stem.2937 30372559 PMC7379496

[B187] Tang Z. Sahu S. N. Khadeer M. A. Bai G. Franklin R. B. Gupta A. (2006). Overexpression of the ZIP1 Zinc Transporter Induces an Osteogenic Phenotype in Mesenchymal Stem Cells. Bone 38 (2), 181–198. 10.1016/j.bone.2005.08.010 16203195

[B188] Tawonsawatruk T. Kelly M. Simpson H. (2014). Evaluation of Native Mesenchymal Stem Cells from Bone Marrow and Local Tissue in an Atrophic Nonunion Model. Tissue Eng. Part C. Methods 20 (6), 524–532. 10.1089/ten.TEC.2013.0465 24147916

[B189] Tella S. H. Gallagher J. C. (2014). Prevention and Treatment of Postmenopausal Osteoporosis. J. Steroid Biochem. Mol. Biol. 142, 155–170. 10.1016/j.jsbmb.2013.09.008 24176761 PMC4187361

[B190] Tessier P. Kawamoto H. Posnick J. Raulo Y. Tulasne J. F. Wolfe S. A. (2005). Complications of Harvesting Autogenous Bone Grafts: a Group Experience of 20,000 Cases. Plastic Reconstr. Surg. 116 (5 Suppl. l), 72s–73s. 10.1097/01.prs.0000173841.59063.7e 16217446

[B191] Vagin V. V. Sigova A. Li C. Seitz H. Gvozdev V. Zamore P. D. (2006). A Distinct Small RNA Pathway Silences Selfish Genetic Elements in the Germline. Science 313 (5785), 320–324. 10.1126/science.1129333 16809489

[B192] Wang C.-G. Hu Y.-H. Su S.-L. Zhong D. (2020). LncRNA DANCR and miR-320a Suppressed Osteogenic Differentiation in Osteoporosis by Directly Inhibiting the Wnt/β-Catenin Signaling Pathway. Exp. Mol. Med. 52 (8), 1310–1325. 10.1038/s12276-020-0475-0 32778797 PMC8080634

[B193] Wang H. Xie Z. Hou T. Li Z. Huang K. Gong J. (2017). MiR-125b Regulates the Osteogenic Differentiation of Human Mesenchymal Stem Cells by Targeting BMPR1b. Cell. Physiol. Biochem. 41 (2), 530–542. 10.1159/000457013 28214897

[B194] Wang H. Meng Y. Cui Q. Qin F. Yang H. Chen Y. (2016). MiR-101 Targets the EZH2/Wnt/β-Catenin the Pathway to Promote the Osteogenic Differentiation of Human Bone Marrow-Derived Mesenchymal Stem Cells. Sci. Rep. 6, 36988. 10.1038/srep36988 27845386 PMC5109541

[B195] Wang H. Tan Z. Hu H. Liu H. Wu T. Zheng C. (2019). microRNA-21 Promotes Breast Cancer Proliferation and Metastasis by Targeting LZTFL1. BMC Cancer 19 (1), 738. 10.1186/s12885-019-5951-3 31351450 PMC6661096

[B196] Wang J. L. Wei X. Wang A. G. Bai Y. Wu X. J. (2020). KCNQ1OT1 Regulates Osteogenic Differentiation of hBMSC by miR-320a/Smad5 axis. Eur. Rev. Med. Pharmacol. Sci. 24 (6), 2843–2854. 10.26355/eurrev_202003_20648 32271402

[B197] Wang L. Qi L. (2021). The Role and Mechanism of Long Non-coding RNA H19 in Stem Cell Osteogenic Differentiation. Mol. Med. 27 (1), 86. 10.1186/s10020-021-00350-y 34384352 PMC8359617

[B198] Wang M. Dai T. Meng Q. Wang W. Li S. (2022). Regulatory Effects of miR-28 on Osteogenic Differentiation of Human Bone Marrow Mesenchymal Stem Cells. Bioengineered 13 (1), 684–696. 10.1080/21655979.2021.2012618 34978269 PMC8805925

[B199] Wang M. Ge X. Zheng Y. Wang C. Zhang Y. Lin Y. (2020). Microarray Analysis Reveals that lncRNA PWRN1 ‐209 Promotes Human Bone Marrow Mesenchymal Stem Cell Osteogenic Differentiation on Microtopography Titanium Surface *In Vitro* . J. Biomed. Mater Res. 108 (7), 2889–2902. 10.1002/jbm.b.34620 32447825

[B200] Wang N. Zhou Z. Wu T. Liu W. Yin P. Pan C. (2016). TNF-α-induced NF-Κb Activation Upregulates microRNA-150-3p and Inhibits Osteogenesis of Mesenchymal Stem Cells by Targeting β-catenin. Open Biol. 6 (3), 150258. 10.1098/rsob.150258 26935950 PMC4821240

[B201] Wang Q. Wang C. H. Meng Y. (2019). microRNA-1297 Promotes the Progression of Osteoporosis through Regulation of Osteogenesis of Bone Marrow Mesenchymal Stem Cells by Targeting WNT5A. Eur. Rev. Med. Pharmacol. Sci. 23 (11), 4541–4550. 10.26355/eurrev_201906_18029 31210281

[B202] Wang Q. Cai J. Cai X.-h. Chen L. (2013). miR-346 Regulates Osteogenic Differentiation of Human Bone Marrow-Derived Mesenchymal Stem Cells by Targeting the Wnt/β-Catenin Pathway. PLoS One 8 (9), e72266. 10.1371/journal.pone.0072266 24023731 PMC3762871

[B203] Wang Q. Li Y. Zhang Y. Ma L. Lin L. Meng J. (2017). LncRNA MEG3 Inhibited Osteogenic Differentiation of Bone Marrow Mesenchymal Stem Cells from Postmenopausal Osteoporosis by Targeting miR-133a-3p. Biomed. Pharmacother. 89, 1178–1186. 10.1016/j.biopha.2017.02.090 28320084

[B204] Wang X. B. Li P. B. Guo S. F. Yang Q. S. Chen Z. X. Wang D. (2019). circRNA_0006393 Promotes Osteogenesis In glucocorticoid-induced O-steoporosis by S-ponging miR-145-5p and U-pregulating FOXO1. Mol. Med. Rep. 20 (3), 2851–2858. 10.3892/mmr.2019.10497 31322188

[B205] Wang X. Chen T. Deng Z. Gao W. Liang T. Qiu X. (2021). Melatonin Promotes Bone Marrow Mesenchymal Stem Cell Osteogenic Differentiation and Prevents Osteoporosis Development through Modulating Circ_0003865 that Sponges miR-3653-3p. Stem Cell. Res. Ther. 12 (1), 150. 10.1186/s13287-021-02224-w 33632317 PMC7908669

[B206] Wei B. Wei W. Zhao B. Guo X. Liu S. (2017). Long Non-coding RNA HOTAIR Inhibits miR-17-5p to Regulate Osteogenic Differentiation and Proliferation in Non-traumatic Osteonecrosis of Femoral Head. PLoS One 12 (2), e0169097. 10.1371/journal.pone.0169097 28207735 PMC5312925

[B207] Wen J. Guan Z. Yu B. Guo J. Shi Y. Hu L. (2020). Circular RNA Hsa_circ_0076906 Competes with OGN for miR-1305 Biding Site to Alleviate the Progression of Osteoporosis. Int. J. Biochem. Cell. Biol. 122, 105719. 10.1016/j.biocel.2020.105719 32087327

[B208] Weng W. Di S. Xing S. Sun Z. Shen Z. Dou X. (2021). Long Non-coding RNA DANCR Modulates Osteogenic Differentiation by Regulating the miR-1301-3p/PROX1 axis. Mol. Cell. Biochem. 476 (6), 2503–2512. 10.1007/s11010-021-04074-9 33629241

[B209] Wong S. K. Chin K.-Y. Ima-Nirwana S. (2020). Quercetin as an Agent for Protecting the Bone: A Review of the Current Evidence. Ijms 21 (17), 6448. 10.3390/ijms21176448 PMC7503351 32899435

[B210] Wu D. Chang X. Tian J. Kang L. Wu Y. Liu J. (2021). Bone Mesenchymal Stem Cells Stimulation by Magnetic Nanoparticles and a Static Magnetic Field: Release of Exosomal miR-1260a Improves Osteogenesis and Angiogenesis. J. Nanobiotechnol 19 (1), 209. 10.1186/s12951-021-00958-6 PMC8278669 34256779

[B211] Wu F. Huang W. Yang Y. Liu F. Chen J. Wang G. (2021). miR‐155‐5p Regulates Mesenchymal Stem Cell Osteogenesis and Proliferation by Targeting GSK3B in Steroid‐associated Osteonecrosis. Cell. Biol. Int. 45 (1), 83–91. 10.1002/cbin.11470 32991030

[B212] Wu H. Cao F. Zhou W. Wang G. Liu G. Xia T. (2020). Long Noncoding RNA FAM83H-AS1 Modulates SpA-Inhibited Osteogenic Differentiation in Human Bone Mesenchymal Stem Cells. Mol. Cell. Biol. 40 (5), e00362–19. 10.1128/mcb.00362-19 31871129 PMC7020645

[B213] Wu J. Zhao J. Sun L. Pan Y. Wang H. Zhang W.-B. (2018). Long Non-coding RNA H19 Mediates Mechanical Tension-Induced Osteogenesis of Bone Marrow Mesenchymal Stem Cells via FAK by Sponging miR-138. Bone 108, 62–70. 10.1016/j.bone.2017.12.013 29253550

[B214] Wu L. Song J. Xue J. Xiao T. Wei Q. Zhang Z. (2020). MircoRNA-143-3p Regulating ARL6 Is Involved in the Cadmium-Induced Inhibition of Osteogenic Differentiation in Human Bone Marrow Mesenchymal Stem Cells. Toxicol. Lett. 331, 159–166. 10.1016/j.toxlet.2020.06.001 32522577

[B215] Wu P. Mo Y. Peng M. Tang T. Zhong Y. Deng X. (2020). Emerging Role of Tumor-Related Functional Peptides Encoded by lncRNA and circRNA. Mol. Cancer 19 (1), 22. 10.1186/s12943-020-1147-3 32019587 PMC6998289

[B216] Wu Z.-h. Huang K.-h. Liu K. Wang G.-t. Sun Q. (2018). DGCR5 Induces Osteogenic Differentiation by Up-Regulating Runx2 through miR-30d-5p. Biochem. Biophysical Res. Commun. 505 (2), 426–431. 10.1016/j.bbrc.2018.09.033 30266402

[B217] Gao X. Xia X. Li F. Zhang M. Zhou H. Wu X. Circular RNA-Encoded Oncogenic E-Cadherin Variant Promotes Glioblastoma Tumorigenicity through Activation of EGFR-STAT3 Signalling. Nat. Cell. Biol., 23(3), 278, 291.+ (2021) 10.1038/s41556-021-00639-4 33664496

[B218] Xia P. Gu R. Zhang W. Shao L. Li F. Wu C. (2019). MicroRNA‐200c Promotes Osteogenic Differentiation of Human Bone Mesenchymal Stem Cells through Activating the AKT/β‐Catenin Signaling Pathway via Downregulating Myd88. J. Cell. Physiology 234 (12), 22675–22686. 10.1002/jcp.28834 31152447

[B219] Xiang J. Fu H. Q. Xu Z. Fan W. J. Liu F. Chen B. (2020). lncRNA SNHG1 Attenuates Osteogenic Differentiation via the miR-101/DKK1 axis in Bone Marrow Mesenchymal Stem Cells. Mol. Med. Rep. 22 (5), 3715–3722. 10.3892/mmr.2020.11489 32901867 PMC7533455

[B220] Xiang S. Li Z. Weng X. (2020). Changed Cellular Functions and Aberrantly Expressed miRNAs and circRNAs in Bone Marrow Stem cells in Osteonecrosis of the Femoral Head. Int. J. Mol. Med. 45 (3), 805–815. 10.3892/ijmm.2020.4455 31922208 PMC7015133

[B221] Xie Y. Hu J. Z. Shi Z. Y. (2018). MiR-181d Promotes Steroid-Induced Osteonecrosis of the Femoral Head by Targeting SMAD3 to Inhibit Osteogenic Differentiation of hBMSCs. Eur. Rev. Med. Pharmacol. Sci. 22 (13), 4053–4062. 10.26355/eurrev_201807_15393 30024590

[B222] Xin C. Liu J. (2021). Long Non-coding RNAs in Parkinson's Disease. Neurochem. Res. 46 (5), 1031–1042. 10.1007/s11064-021-03230-3 33544326

[B223] Xin W. Yuan S. Wang B. Qian Q. Chen Y. (2021). Hsa_circ_0066523 Promotes the Proliferation and Osteogenic Differentiation of Bone Mesenchymal Stem Cells by Repressing PTEN. Bone & Jt. Res. 10 (8), 526–535. 10.1302/2046-3758.108.Bjr-2020-0127.R2 PMC8414438 34402627

[B224] Xing Y.-H. Chen L.-L. (2018). Processing and Roles of snoRNA-Ended Long Noncoding RNAs. Crit. Rev. Biochem. Mol. Biol. 53 (6), 596–606. 10.1080/10409238.2018.1508411 30252509

[B225] Xu G. Ding Z. Shi H.-f. (2019). The Mechanism of miR-889 Regulates Osteogenesis in Human Bone Marrow Mesenchymal Stem Cells. J. Orthop. Surg. Res. 14 (1), 366. 10.1186/s13018-019-1399-z 31727100 PMC6854696

[B226] Xu H. Wang J. Ye X. Ming J. Li S. J. Wang X. (2018). Effect of MiR-99a-5p on the Differential Ability of Human Bone Marrow Mesenchymal Stem Cells. Zhongguo Shi Yan Xue Ye Xue Za Zhi 26 (2), 563–568. 10.7534/j.issn.1009-2137.2018.02.043 29665933

[B227] Xu J.-F. Yang G.-h. Pan X.-H. Zhang S.-J. Zhao C. Qiu B.-S. (2014). Altered microRNA Expression Profile in Exosomes during Osteogenic Differentiation of Human Bone Marrow-Derived Mesenchymal Stem Cells. PLoS One 9 (12), e114627. 10.1371/journal.pone.0114627 25503309 PMC4263734

[B228] Xu J. Yang X. Zhou Q. Zhuang J. Han S. (2020). Biological Significance of piRNA in Liver Cancer: a Review. Biomarkers 25 (6), 436–440. 10.1080/1354750x.2020.1794041 32662667

[B229] Xu S. Cecilia Santini G. De Veirman K. Vande Broek I. Leleu X. De Becker A. (2013). Upregulation of miR-135b Is Involved in the Impaired Osteogenic Differentiation of Mesenchymal Stem Cells Derived from Multiple Myeloma Patients. PLoS One 8 (11), e79752. 10.1371/journal.pone.0079752 24223191 PMC3819242

[B230] Yang C. Liu X. Zhao K. Zhu Y. Hu B. Zhou Y. (2019). miRNA-21 Promotes Osteogenesis via the PTEN/PI3K/Akt/HIF-1α Pathway and Enhances Bone Regeneration in Critical Size Defects. Stem Cell. Res. Ther. 10 (1), 65. 10.1186/s13287-019-1168-2 30795815 PMC6387542

[B231] Yang N. Wang G. Hu C. Shi Y. Liao L. Shi S. (2013). Tumor Necrosis Factor α Suppresses the Mesenchymal Stem Cell Osteogenesis Promoter miR-21 in Estrogen Deficiency-Induced Osteoporosis. J. Bone Min. Res. 28 (3), 559–573. 10.1002/jbmr.1798 23074166

[B232] Yang Q. Jia L. Li X. Guo R. Huang Y. Zheng Y. (2018). Long Noncoding RNAs: New Players in the Osteogenic Differentiation of Bone Marrow- and Adipose-Derived Mesenchymal Stem Cells. Stem Cell. Rev Rep 14 (3), 297–308. 10.1007/s12015-018-9801-5 29464508

[B233] Yang Q. Zhou Y. Wang T. Cai P. Fu W. Wang J. (2021). MiRNA‐1271‐5p Regulates Osteogenic Differentiation of Human Bone Marrow‐derived Mesenchymal Stem Cells by Targeting Forkhead Box O1 (FOXO1). Cell. Biol. Int. 45 (7), 1468–1476. 10.1002/cbin.11585 33675274

[B234] Yang W. Zhu W. Yang Y. Guo M. Qian H. Jiang W. (2021). Exosomal miR-100-5p Inhibits Osteogenesis of hBMSCs and Angiogenesis of HUVECs by Suppressing the BMPR2/Smad1/5/9 Signalling Pathway. Stem Cell. Res. Ther. 12 (1), 390. 10.1186/s13287-021-02438-y 34256859 PMC8278698

[B235] Yang X. Yang J. Lei P. Wen T. (2019). LncRNA MALAT1 Shuttled by Bone Marrow-Derived Mesenchymal Stem Cells-Secreted Exosomes Alleviates Osteoporosis through Mediating microRNA-34c/SATB2 axis. Aging 11 (20), 8777–8791. 10.18632/aging.102264 31659145 PMC6834402

[B236] You L. Pan L. Chen L. Gu W. Chen J. (2016). MiR-27a Is Essential for the Shift from Osteogenic Differentiation to Adipogenic Differentiation of Mesenchymal Stem Cells in Postmenopausal Osteoporosis. Cell. Physiol. Biochem. 39 (1), 253–265. 10.1159/000445621 27337099

[B237] Younger E. M. Chapman M. W. (1989). Morbidity at Bone Graft Donor Sites. J. Orthop. Trauma 3 (3), 192–195. 10.1097/00005131-198909000-00002 2809818

[B238] Yu L. Xu Y. Qu H. Yu Y. Li W. Zhao Y. (2019). Decrease of MiR‐31 Induced by TNF‐α Inhibitor Activates SATB2/RUNX2 Pathway and Promotes Osteogenic Differentiation in Ethanol‐induced Osteonecrosis. J. Cell. Physiology 234 (4), 4314–4326. 10.1002/jcp.27210 30132874

[B239] Zang J. Lu D. Xu A. (2020). The Interaction of circRNAs and RNA Binding Proteins: An Important Part of circRNA Maintenance and Function. J. Neurosci. Res. 98 (1), 87–97. 10.1002/jnr.24356 30575990

[B240] Zealy R. W. Wrenn S. P. Davila S. Min K.-W. Yoon J.-H. (2017). microRNA-Binding Proteins: Specificity and Function. WIREs RNA 8 (5), e1414. 10.1002/wrna.1414 28130820

[B241] Zeng H.-B. Dong L.-Q. Huang Y.-L. Xu C. Zhao X.-H. Wu L.-G. (2021). USF2 Reduces BMP3 Expression via Transcriptional Activation of miR-34a, Thus Promoting Osteogenic Differentiation of BMSCs. J. Bone Min. Metab. 39 (6), 997–1008. 10.1007/s00774-021-01254-x 34350522

[B242] Zha J.-p. Wang X.-q. Di J. (2020). MiR-920 Promotes Osteogenic Differentiation of Human Bone Mesenchymal Stem Cells by Targeting HOXA7. J. Orthop. Surg. Res. 15 (1), 254. 10.1186/s13018-020-01775-7 32650806 PMC7350748

[B243] Zhang G. P. Zhang J. Zhu C. H. Lin L. Wang J. Zhang H. J. (2017). Micro RNA ‐98 Regulates Osteogenic Differentiation of Human Bone Mesenchymal Stromal Cells by Targeting BMP 2. J. Cell. Mol. Med. 21 (2), 254–264. 10.1111/jcmm.12961 27860183 PMC5264139

[B244] Zhang H. L. Du X. Y. Dong Q. R. (2019). LncRNA XIXT Promotes Osteogenic Differentiation of Bone Mesenchymal Stem Cells and Alleviates Osteoporosis Progression by Targeting miRNA-30a-5p. Eur. Rev. Med. Pharmacol. Sci. 23 (20), 8721–8729. 10.26355/eurrev_201910_19266 31696458

[B245] Zhang J. Chen S. Liu K. (2022). Structural Insights into piRNA Biogenesis. Biochimica Biophysica Acta (BBA) - Gene Regul. Mech. 1865 (2), 194799. 10.1016/j.bbagrm.2022.194799 35182819

[B246] Zhang J. Tao Z. Wang Y. (2018). Long Non-coding RNA DANCR R-egulates the P-roliferation and O-steogenic D-ifferentiation of H-uman B-one-D-erived M-arrow M-esenchymal S-tem C-ells via the p38ï¿½MAPK P-athway. Int. J. Mol. Med. 41 (1), 213–219. 10.3892/ijmm.2017.3215 29115577 PMC5746326

[B247] Zhang L. Liu Y. Feng B. Liu L.-G. Zhou Y.-C. Tang H. (2021). MiR-138-5p Knockdown Promotes Osteogenic Differentiation through FOXC1 Up-Regulation in Human Bone Mesenchymal Stem Cells. Biochem. Cell. Biol. 99 (3), 296–303. 10.1139/bcb-2020-0163 33058690

[B248] Zhang S. Liu Y. Zheng Z. Zeng X. Liu D. Wang C. (2018). MicroRNA-223 Suppresses Osteoblast Differentiation by Inhibiting DHRS3. Cell. Physiol. Biochem. 47 (2), 667–679. 10.1159/000490021 29794437

[B249] Zhang W. Chen L. Wu J. Li J. Zhang X. Xiang Y. (2019). Long Noncoding RNA TUG1 Inhibits Osteogenesis of Bone Marrow Mesenchymal Stem Cells via Smad5 after Irradiation. Theranostics 9 (8), 2198–2208. 10.7150/thno.30798 31149038 PMC6531293

[B250] Zhang Y. Chen B. Li D. Zhou X. Chen Z. (2019). LncRNA NEAT1/miR-29b-3p/BMP1 axis Promotes Osteogenic Differentiation in Human Bone Marrow-Derived Mesenchymal Stem Cells. Pathology - Res. Pract. 215 (3), 525–531. 10.1016/j.prp.2018.12.034 30638953

[B251] Zhang Y. Jiang Y. Luo Y. Zeng Y. (2020). Interference of miR-212 and miR-384 Promotes Osteogenic Differentiation via Targeting RUNX2 in Osteoporosis. Exp. Mol. Pathology 113, 104366. 10.1016/j.yexmp.2019.104366 31891679

[B252] Zhang L. Xie H. Li S. (2020). LncRNA LOXL1-AS1 Controls Osteogenic and Adipocytic Differentiation of Bone Marrow Mesenchymal Stem Cells in Postmenopausal Osteoporosis through Regulating the miR-196a-5p/Hmga2 axis. J. Bone Min. Metab. 38 (6), 794–805. 10.1007/s00774-020-01123-z 32651705

[B253] Zhang Y. Liu Y. Wu M. Wang H. Wu L. Xu B. (2020a). MicroRNA-664a-5p Promotes Osteogenic Differentiation of Human Bone Marrow-Derived Mesenchymal Stem Cells by Directly Downregulating HMGA2. Biochem. Biophysical Res. Commun. 521 (1), 9–14. 10.1016/j.bbrc.2019.09.122 31630797

[B254] Zhang Y. Zhou L. Zhang Z. Ren F. Chen L. Lan Z. (2020b). miR-10a-5p I-nhibits O-steogenic D-ifferentiation of B-one M-arrow-derived M-esenchymal S-tem C-ells. Mol. Med. Rep. 22 (1), 135–144. 10.3892/mmr.2020.11110 32377690 PMC7248527

[B255] Zhang Y. Sun Y. Liu J. Han Y. Yan J. (2020). MicroRNA-346-5p Regulates Differentiation of Bone Marrow-Derived Mesenchymal Stem Cells by Inhibiting Transmembrane Protein 9. BioMed Res. Int. 2020, 1–7. 10.1155/2020/8822232 PMC7704134 33299881

[B256] Zhang Y. Sun Y. Zhang Y. (2015). MiR-133 Is Involved in Estrogen Deficiency-Induced Osteoporosis through Modulating Osteogenic Differentiation of Mesenchymal Stem Cells. Med. Sci. Monit. 21, 1527–1534. 10.12659/msm.894323 26013661 PMC4459570

[B257] Zhang Y. Wang X. (2020). Targeting the Wnt/β-Catenin Signaling Pathway in Cancer. J. Hematol. Oncol. 13 (1), 165. 10.1186/s13045-020-00990-3 33276800 PMC7716495

[B258] Zhang Y. Wei Q.-S. Ding W.-B. Zhang L.-L. Wang H.-C. Zhu Y.-J. (2017). Increased microRNA-93-5p Inhibits Osteogenic Differentiation by Targeting Bone Morphogenetic Protein-2. PLoS One 12 (8), e0182678. 10.1371/journal.pone.0182678 28797104 PMC5552299

[B259] Zhang Z. Jiang W. Hu M. Gao R. Zhou X. (2021). MiR-486-3p Promotes Osteogenic Differentiation of BMSC by Targeting CTNNBIP1 and Activating the Wnt/β-Catenin Pathway. Biochem. Biophysical Res. Commun. 566, 59–66. 10.1016/j.bbrc.2021.05.098 34118593

[B260] Zhao J. Wang C. Song Y. Fang B. (2014). Arsenic Trioxide and microRNA-204 Display Contrary Effects on Regulating Adipogenic and Osteogenic Differentiation of Mesenchymal Stem Cells in Aplastic Anemia. Acta Biochim. Biophys. Sin. (Shanghai) 46 (10), 885–893. 10.1093/abbs/gmu082 25187411

[B261] Zhao L. Zhao J. Yu J.-J. Zhang C. (2020). Irregular Bone Defect Repair Using Tissue-Engineered Periosteum in a Rabbit Model. Tissue Eng. Regen. Med. 17 (5), 717–727. 10.1007/s13770-020-00282-4 32914288 PMC7524931

[B262] Zhao R. Li Y. Lin Z. Wan J. Xu C. Zeng Y. (2016). miR-199b-5p Modulates BMSC Osteogenesis via Suppressing GSK-3β/β-Catenin Signaling Pathway. Biochem. Biophysical Res. Commun. 477 (4), 749–754. 10.1016/j.bbrc.2016.06.130 27363340

[B263] Zhao Y. Chen Y. Hu X. Zhang N. Wang F. (2020). lncRNA LINC01535 Upregulates BMP2 Expression Levels to Promote Osteogenic Differentiation via Sponging miR-3619-5p. Mol. Med. Rep. 22 (6), 5428–5435. 10.3892/mmr.2020.11635 33174047

[B264] Zhao Y. S. Lin P. Tu Y. C. An T. Wu Y. P. Li X. F. (2021). Lentivirus Mediated siRNA Hsa-Circ-0000885 Transfection of BMSCs and Osteoclast Co-culture System on Cell Differentiation, Proliferation and Apoptosis. Zhongguo Gu Shang 34 (10), 978–984. 10.12200/j.issn.1003-0034.2021.10.017 34726029

[B265] Zhao Y. Wang Z. Wu G. Wei M. Liu Q. Zhou J. (2016). Improving the Osteogenesis of Human Bone Marrow Mesenchymal Stem Cell Sheets by microRNA-21-Loaded Chitosan/hyaluronic Acid Nanoparticles via Reverse Transfection. Ijn 11, 2091–2105. 10.2147/ijn.S104851 27274237 PMC4876805

[B266] Zheng C. Bai C. Sun Q. Zhang F. Yu Q. Zhao X. (2020). Long Noncoding RNA XIST Regulates Osteogenic Differentiation of Human Bone Marrow Mesenchymal Stem Cells by Targeting miR-9-5p. Mech. Dev. 162, 103612. 10.1016/j.mod.2020.103612 32389806

[B267] Zhi F. Ding Y. Wang R. Yang Y. Luo K. Hua F. (2021). Exosomal Hsa_circ_0006859 Is a Potential Biomarker for Postmenopausal Osteoporosis and Enhances Adipogenic versus Osteogenic Differentiation in Human Bone Marrow Mesenchymal Stem Cells by Sponging miR-431-5p. Stem Cell. Res. Ther. 12 (1), 157. 10.1186/s13287-021-02214-y 33648601 PMC7923524

[B268] Zhou P. Wu G. Zhang P. Xu R. Ge J. Fu Y. (2016). SATB2-Nanog axis Links Age-Related Intrinsic Changes of Mesenchymal Stem Cells from Craniofacial Bone. Aging 8 (9), 2006–2022. 10.18632/aging.101041 27632702 PMC5076449

[B269] Zhou X. Moussa F. M. Mankoci S. Ustriyana P. Zhang N. Abdelmagid S. (2016). Orthosilicic Acid, Si(OH)4, Stimulates Osteoblast Differentiation *In Vitro* by Upregulating miR-146a to Antagonize NF-Κb Activation. Acta Biomater. 39, 192–202. 10.1016/j.actbio.2016.05.007 27163405

[B270] Zhu J. Fu H. Wu Y. Zheng X. (2013). Function of lncRNAs and Approaches to lncRNA-Protein Interactions. Sci. China Life Sci. 56 (10), 876–885. 10.1007/s11427-013-4553-6 24091684

[B271] Zhu L. Li J. Gong Y. Wu Q. Tan S. Sun D. (2019). Exosomal tRNA-Derived Small RNA as a Promising Biomarker for Cancer Diagnosis. Mol. Cancer 18 (1), 74. ARTN 74. 10.1186/s12943-019-1000-8 30940133 PMC6444574

[B272] Zhuang Q. Ye B. Hui S. Du Y. Zhao R. C. Li J. (2019). Long Noncoding RNA lncAIS Downregulation in Mesenchymal Stem Cells Is Implicated in the Pathogenesis of Adolescent Idiopathic Scoliosis. Cell. Death Differ. 26 (9), 1700–1715. 10.1038/s41418-018-0240-2 30464226 PMC6748078

[B273] Zhuang X.-m. Zhou B. (2020). Exosome Secreted by Human Gingival Fibroblasts in Radiation Therapy Inhibits Osteogenic Differentiation of Bone Mesenchymal Stem Cells by Transferring miR-23a. Biomed. Pharmacother. 131, 110672. 10.1016/j.biopha.2020.110672 32889404

[B274] Zhuang X.-M. Zhou B. Yuan K.-F. (2019). Role of P53 Mediated miR-23a/CXCL12 Pathway in Osteogenic Differentiation of Bone Mesenchymal Stem Cells on Nanostructured Titanium Surfaces. Biomed. Pharmacother. 112, 108649. 10.1016/j.biopha.2019.108649 30784930

[B275] Zong T. Yang Y. Zhao H. Li L. Liu M. Fu X. (2021). tsRNAs: Novel Small Molecules from Cell Function and Regulatory Mechanism to Therapeutic Targets. Cell. Prolif. 54 (3), e12977. 10.1111/cpr.12977 33507586 PMC7941233

